# Healthy lifestyle, metabolomic signature, and risk of late-onset schizophrenia: evidence from the prospective cohort

**DOI:** 10.1038/s41537-026-00752-z

**Published:** 2026-04-14

**Authors:** Xinru Guo, Ge Yu, Songyu Wu, Tingyi Jia, Zhouyang Sun, Changgui Kou, Wei Bai

**Affiliations:** 1 Department of Epidemiology and Biostatistics, School of Public Health, Jilin University, Changchun, China; 2 Department of Social Medicine and Health Management, School of Public Health, Jilin University, Changchun, China

**Keywords:** Schizophrenia, Biomarkers

## Abstract

A healthy lifestyle is associated with a reduced risk of schizophrenia, but the underlying metabolic mechanisms remain unclear. The aim of this study was to identify a metabolomic signature of a healthy lifestyle, to assess its mediation between lifestyle and schizophrenia risk, and to evaluate its potential causal link to schizophrenia. This study included 170,783 participants from the UK Biobank with comprehensive data on lifestyle, metabolomics, and relevant covariates. An elastic net regression model was employed to construct a metabolomic signature reflecting a healthy lifestyle. Associations between this signature and schizophrenia risk were examined using Cox proportional hazards models. Mediation analysis was conducted to assess the mediating role of the metabolomic signature in the association between healthy lifestyle and schizophrenia onset, while Mendelian randomization (MR) analysis was performed to explore potential causal effects. Individuals with a healthy lifestyle had a 58% lower risk of schizophrenia compared to those with an unhealthy lifestyle (HR 0.42; 95% CI, 0.29–0.61). The metabolomic signature, comprising 113 metabolites, was strongly correlated with the healthy lifestyle (*r* = 0.36, *P* < 0.001) and associated with reduced schizophrenia risk (HR 0.62 per SD increase; 95% CI, 0.49–0.79). This signature accounted for 15.59% of the association between healthy lifestyle and schizophrenia risk, and MR analysis suggested a possible causal relationship. Our study revealed a potential link between healthy lifestyle, metabolomic signature, and late-onset schizophrenia, highlighting the potential role of lifestyle-related metabolic alterations in schizophrenia development.

## Main

Schizophrenia affects approximately 1% of the global population and is a severe mental illness associated with a substantially reduced life expectancy compared to the general population^1,2^. Schizophrenia with onset after 40 years of age is generally considered late-onset^3^. While high heritability estimates suggest a strong genetic component, the onset of schizophrenia is also influenced by a range of environmental factors^4^. Increasing research attention has been directed toward the cumulative impact of these environmental risk factors, as individuals with schizophrenia often experience lower socioeconomic status, social isolation, and related challenges, which may contribute to unhealthy lifestyle behaviors^5–8^.

Converging evidence indicates that interventions targeting physical activity, smoking, diet, and sleep play a critical role in the prevention of mental disorders^9^. Consistent findings from prospective observational studies and Mendelian randomization (MR) analyses suggest that smoking is a risk factor for the onset of schizophrenia^10–12^. Similarly, evidence from prospective cohort studies supports physical activity as a protective factor, while alcohol consumption has been identified as a potential risk factor^13,14^. In addition, social isolation has emerged as an important environmental contributor to schizophrenia risk^15^. Non-sedentary behavior, a newly recognized component of a healthy lifestyle, has been extensively studied in relation to various health outcomes, particularly cognitive function and dementia^16,17^. Compared to evaluating individual lifestyle factors in isolation, analyzing them in combination may provide a more comprehensive reflection of daily behavioral patterns and help reveal potential synergistic effects^18^. However, existing research on healthy lifestyle factors in the context of schizophrenia has largely focused on their impact on comorbidities—such as cardiovascular disease and type 2 diabetes—or on premature mortality after disease onset^2,19,20^. In contrast, studies investigating the combined influence of healthy lifestyle behaviors on the risk of developing schizophrenia remain limited.

High-throughput metabolomics provides a comprehensive and dynamic view of biological processes, enabling assessment of the integrated effects of genetic predisposition, lifestyle factors, and environmental exposures^21^. An increasing body of research has highlighted the value of metabolomic signatures in elucidating the biological mechanisms underlying the associations between lifestyle behaviors and various disease outcomes, including chronic conditions such as type 2 diabetes, cardiovascular disease, cancer, and dementia^18,22–24^. However, the associations among metabolomic signatures, healthy lifestyle patterns, and the risk of schizophrenia remain largely unexplored.

The UK Biobank is a large, population-based prospective cohort comprising more than 500,000 participants, providing a robust resource for investigating late-onset schizophrenia. In this study, we leveraged lifestyle and metabolomic data from the UK Biobank to identify metabolomic signatures associated with a healthy lifestyle and to evaluate their relationship with schizophrenia risk.

## Methods

### Study population

**Fig. 1:**
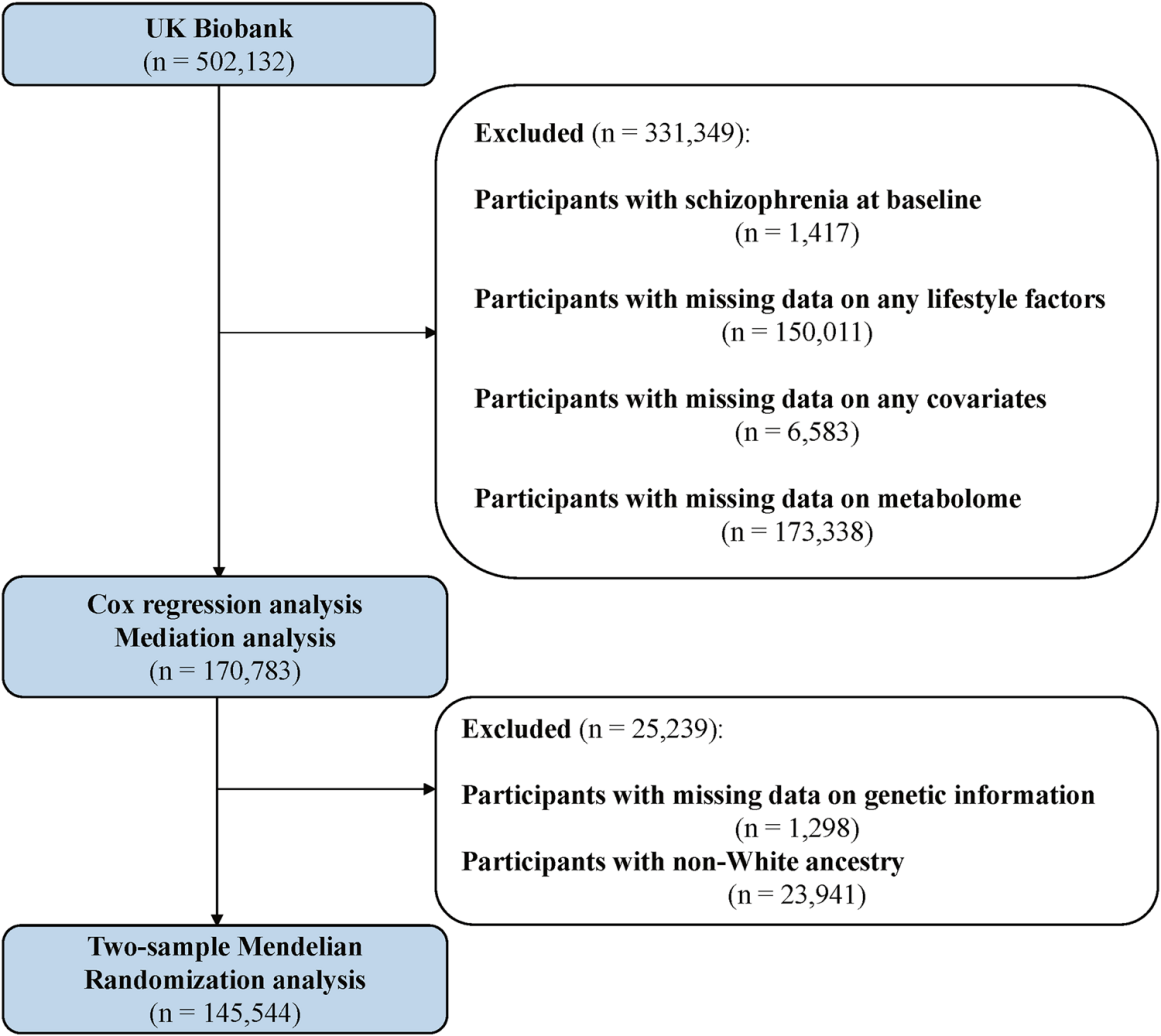
Flowchart of the UK Biobank participants included and excluded in study. The diagram shows the inclusion and exclusion process of participants, along with the final study population.

The UK Biobank is a large, prospective cohort study that recruited over 500,000 participants aged 37 to 73 years between 2006 and 2010 from 22 assessment centers across England, Scotland, and Wales. Detailed study protocols and participant characteristics have been described elsewhere^25^. Lifestyle factors were assessed at baseline using standardized questionnaires capturing participants’ habitual behaviors prior to enrollment (e.g., smoking, alcohol consumption, physical activity, diet, sleep duration, sedentary behavior, and social contact). Blood samples were collected during the same visit, and metabolomic biomarkers were measured from these samples. The primary analysis included 170,783 individuals who were free of schizophrenia at baseline and had complete data on healthy lifestyle scores, covariates, and metabolomic profiles. Genomic analyses were conducted in a subset of 145,544 participants, as illustrated in Fig. 1.

### Construction of the healthy lifestyle score

In this study, an overall healthy lifestyle score was constructed based on seven modifiable behavioral factors, including four traditional factors (smoking, alcohol consumption, physical activity, and diet) and three emerging factors (sleep duration, sedentary behavior, and social contact). This composite score has been widely utilized in prior research^26–28^. All lifestyle information was self-reported via touchscreen questionnaires at baseline, and detailed assessments of the lifestyle factors are summarized in Supplementary Table 1. The healthy lifestyle score in this study was calculated by summing seven binary lifestyle factors (0 = unhealthy behavior, 1 = healthy behavior), resulting in a total score ranging from 0 to 7, with higher scores indicating a healthier lifestyle. Never smoking was considered a healthy behavior. Moderate alcohol consumption, defined as no more than one standard drink per day (14 g of ethanol) for women and no more than two for men (28 g of ethanol), was regarded as another indicator of a healthy lifestyle^29^. Regular physical activity was characterized by weekly participation in at least 150 min of moderate or 75 min of vigorous exercise, or their equivalent^26^. Healthy diet was defined as the adequate consumption of at least four out of seven dietary components, including increased intake of fruits, vegetables, whole grains, and fish, as well as reduced intake of refined grains and both processed and unprocessed meats (Supplementary Table 2)^30^. A sleep duration of 7–8 h per day was defined as healthy, and television watching time of less than 4 h per day was considered a healthy level of sedentary behavior. The social contact index was constructed based on the number of household members, the frequency of visits with friends or family, and participation in leisure or social activities. A healthy status was defined as having frequent social interactions^31^. To investigate the associations of lifestyle with health outcomes, participants were divided into 3 groups based on their lifestyle scores: those scoring 0–2 were classified as having the unhealthy lifestyle, those scoring 3–5 as moderately healthy, and those scoring 6–7 as having the healthy lifestyle^28^.

### Assessment of schizophrenia and covariates

The assessment of schizophrenia was based on data from primary care records, hospital inpatient admissions, death registry data, and self-reported diagnoses within the UK Biobank. Diagnoses were identified using codes F20–F29 from ICD-10 (late-onset schizophrenia defined as onset at ≥40 years). Follow-up time was calculated from the date of attendance at the assessment center to the earliest occurrence of a schizophrenia event, death, loss to follow-up, or December 19, 2022, whichever came first. The covariates included in this study were age, sex, ethnicity, education level, Townsend deprivation index, body mass index (BMI), and medication history (including antidiabetic, antihypertensive, and lipid-lowering medications). The top 10 genetic principal components (PCs 1–10) and genotyping array (UK BiLEVE array and UK Biobank Axiom array) were included in the genetic analyses.

### Measurement of metabolic biomarkers and genotyping process

Approximately 280,000 EDTA-treated blood samples from UK Biobank participants underwent nuclear magnetic resonance (NMR)-based metabolomic profiling using the Nightingale Health platform, resulting in quantification of 251 metabolic biomarkers—170 measured in absolute concentrations and 81 reported as composite ratio indices. Further details are provided in Supplementary Table 3. Analytical procedures and quality control protocols have been described in previous studies^32^. Prior to analysis, all 251 metabolites were natural log–transformed and standardized to Z-scores. Pearson correlation coefficients were calculated to assess intercorrelations among the metabolites.

Genotyping in the UK Biobank was performed using the UK BiLEVE or UK Biobank Axiom arrays, with imputation based on the 97 merged UK10K and 1000 Genomes phase 3 panels^33^. Single nucleotide polymorphisms (SNPs) were excluded if they had an INFO score below 0.3, a minor allele frequency less than 0.5%, or showed significant departure from Hardy–Weinberg equilibrium (*P* < 1 × 10⁻⁶).

### Statistical analysis

Baseline characteristics of the study participants were summarized as percentages for categorical variables and compared between groups using the chi-square test. Firstly, elastic net regression was employed to regress the healthy lifestyle score on 251 standardized metabolic biomarkers to construct a metabolomic signature reflecting the healthy lifestyle. Elastic net regression is a regularized regression method that combines Lasso and Ridge penalties, offering advantages in handling multicollinearity and reducing overfitting^34^. In this study, we selected the optimal lambda parameter using a 10-fold cross-validation approach, choosing the largest value of lambda for which the mean squared error was within one standard deviation of the minimum. The metabolomic signature was calculated as a weighted sum of metabolites with non-zero coefficients, where the weights corresponded to their respective coefficients. Metabolite concentrations were standardized prior to model fitting, and the signature score for each participant was calculated by multiplying the standardized metabolite values by their corresponding elastic-net coefficients and summing across all selected metabolites. This signature was then classified into three categories: low (quintile 1), intermediate (quintiles 2–4), and high (quintile 5). This categorization was used to contrast extreme exposure levels while maintaining statistical stability within the intermediate range. Principal component analysis (PCA) was performed on the selected metabolites to facilitate biological interpretation of the metabolomic signature.

Secondly, Cox proportional hazards models were used to examine the associations of the healthy lifestyle score and metabolomic signature with the risk of schizophrenia, reporting hazard ratios (HRs) and 95% confidence intervals (CIs). We constructed three sequential models for analysis. Model 1 was adjusted for age and sex. Model 2 was additionally adjusted for ethnicity, Townsend deprivation index, education level, BMI, as well as a range of medication use, including antidiabetic, antihypertensive, and lipid-lowering drugs. Model 3 additionally incorporated both the healthy lifestyle score and the metabolomic signature into a mutually adjusted model to examine the independence of their associations. All analyses were conducted using both categorical and continuous forms of these variables. The proportional hazards assumption was assessed by testing the significance of Schoenfeld residuals. To further evaluate potential dose–response relationships, the metabolomic signature was also analyzed as a continuous variable in Cox regression models. In addition, Kaplan–Meier curves were constructed to illustrate the cumulative incidence of schizophrenia across categories of the metabolomic signature, and differences between groups were assessed using the log-rank test.

Thirdly, we explored the contribution of distinct metabolic pathways to these associations and examined the mediating effects of individual metabolites comprising the metabolomic signatures on the relationship between lifestyle factors and the risk of schizophrenia. We conducted causal mediation analysis using the CMAverse package in R^35^. A randomization-based framework was employed to estimate the natural direct and indirect effects of the exposure on a time-to-event outcome through the mediator. A linear regression model was used for the mediator, and a Cox proportional hazards model was specified for the outcome. The proportion mediated was calculated to quantify the extent to which the mediator accounted for the total effect.

Fourthly, a Genome-Wide Association Study (GWAS) was performed to identify novel associations between chromosomal loci and the metabolomic signature, as well as to detect significant SNPs. Associations between genotypes and the metabolomic signature were assessed using linear regression under an additive genetic model. All models were adjusted for potential confounders, including age, sex, the healthy lifestyle score, PCs 1–10, genotyping array, and assessment center. Based on summary statistics from the schizophrenia GWAS published by Pedersen EM et al. in 2023^36^—which included 48,523 individuals of European ancestry from Denmark—we conducted two-sample MR analysis to investigate the potential causal relationship between the metabolomic signature and schizophrenia risk, adhering to the three core instrumental variable assumptions described previously^37^. Genetic instruments were selected using a genome-wide significance threshold (*P* < 5 × 10⁻⁸). To ensure the independence of selected variants, linkage disequilibrium (LD) clumping was performed using an r² threshold of 0.001 and a physical distance window of 10,000 kb. The inverse-variance weighted (IVW) method served as the primary analytic approach, while the weighted median and MR-Egger methods were employed as complementary sensitivity analyses. Instrument strength was assessed using the F-statistic (mean *F* = 29.24), confirming that the selected SNPs provided sufficiently strong instruments. Heterogeneity was evaluated using Cochran’s Q statistic, and horizontal pleiotropy was examined via the MR-Egger intercept. Additionally, leave-one-out analyses were conducted to assess whether the MR estimates were driven by any single SNP. Harmonization procedures were applied to align effect alleles between exposure and outcome GWAS datasets, and palindromic SNPs with ambiguous strand orientation were excluded.

Subgroup analyses were conducted by stratifying participants according to age (<50 years, 50–60 years, or ≥60 years) and sex (female or male). Additional stratified analyses were conducted across key sociodemographic and clinical variables, and according to schizophrenia polygenic risk score (PRS). Additional sensitivity analyses included using alternative outcome definitions—specifically, F20 only; F20 and F25 combined; or F20–F29 excluding F23—excluding participants who developed schizophrenia within the first 2 or 5 years of follow-up, and excluding participants with baseline all-cause dementia (F00, F02, F03, or G30) or organic psychosis (F06 or F07). Analyses were further adjusted for history of cancer and cardiovascular disease. Missing lifestyle variables were imputed using multiple imputation by chained equations implemented with the mice package in R (five imputations, ten iterations), and competing-risk analyses were conducted using the Fine–Gray model treating death as a competing event. As another sensitivity analysis, the metabolomic signature was additionally categorized into quintiles (Q1–Q5) and tested for linear trend.

All statistical analyses were performed using R software (version 4.2.0). A two-sided *P* value < 0.05 was considered indicative of statistical significance.

### Ethics approval and consent to participate

The data used in this study were obtained from the UK Biobank (application number 452018), with ethical approval (REC No. 21/NW/0157) and informed consent from participants. The study was conducted in accordance with the Declaration of Helsinki.

## Results

### Characteristics of the participants

During a mean follow-up period of 13.4 years (standard deviation, SD: 2), 400 participants developed schizophrenia. At baseline, participants’ ages in the overall cohort ranged from 38 to 73 years. Among the 400 incident schizophrenia cases, baseline age ranged from 40 to 69 years (median 58 years, interquartile range 50–64, mean ± SD 56.8 ± 8.6) (Supplementary Table 4). Table 1 summarizes the baseline characteristics of the study participants. This study included 170,783 participants, of whom 72,974 (42.73%) were aged 60 years and older. Among the total population, 85,891 (50.29%) were men. Patients with schizophrenia were more likely to be non-White, have a higher Townsend deprivation index, have no history of attending college, and have a higher prevalence of prior use of antidiabetic, antihypertensive, and lipid-lowering medications. Participants who developed schizophrenia showed a higher prevalence of unhealthy lifestyle patterns compared with those without schizophrenia and were more likely to exhibit unfavorable profiles in physical activity, smoking, sleep duration, social contact, and sedentary behavior. Furthermore, we observed that the characteristics identified at baseline were largely consistent with that observed in the first repeat assessment (Supplementary Table 5).

**Table 1:** Baseline characteristics of participants stratified by incident schizophrenia in the UK Biobank.

Characteristics^a^	Total	Non-schizophrenia	Schizophrenia	*P* value
Age				
<50 years	40,696 (23.83)	40,596 (23.83)	100 (25.00)	0.106
50–60 years	57,113 (33.44)	56,999 (33.45)	114 (28.50)	
≥60 years	72,974 (42.73)	72,788 (42.72)	186 (46.50)	
Male	85,891 (50.29)	85,681 (50.29)	210 (52.50)	0.404
White	163,714 (95.86)	163,356 (95.88)	358 (89.50)	<0.001
Townsend deprivation index (above median)	80,374 (47.06)	80,121 (47.02)	253 (63.25)	<0.001
College	60,748 (35.57)	60,631 (35.59)	117 (29.25)	0.010
Body mass index (≥18.5 & ≤ 24.9, kg/m^2^)	117,276 (68.67)	116,988 (68.66)	288 (72.00)	0.166
Glucose lowering drug use	5491 (3.22)	5467 (3.21)	24 (6.00)	0.003
Blood pressure medication use	34,354 (20.12)	34,242 (20.10)	112 (28.00)	<0.001
Lipid lowering medication use	29,569 (17.31)	29,470 (17.30)	99 (24.75)	<0.001
Healthy lifestyle scores				
Healthy	45,478 (26.63)	45,411 (26.65)	67 (16.75)	<0.001
Moderately healthy	11,3607 (66.52)	11,3334 (66.52)	273 (68.25)	
Unhealthy	11,698 (6.85)	11,638 (6.83)	60 (15.00)	
Lifestyle components				
Drinking				
Excess	86,024 (50.37)	85,803 (50.36)	221 (55.25)	0.057
Moderate	84,759 (49.63)	84,580 (49.64)	179 (44.75)	
Diet				
Unideal	93,258 (54.61)	93,022 (54.60)	236 (59.00)	0.086
Ideal	77,525 (45.39)	77,361 (45.40)	164 (41.00)	
Physical activity				
Lacking	32,230 (18.87)	32,127 (18.86)	103 (25.75)	<0.001
Regular	138,553 (81.13)	138,256 (81.14)	297 (74.25)	
Smoking				
Previous/Current	77,719 (45.51)	77,499 (45.49)	220 (55.00)	<0.001
Never	93,064 (54.49)	92,884 (54.51)	180 (45.00)	
Sleep duration				
<7 h/day or >8 h/day	52,796 (30.91)	52,624 (30.89)	172 (43.00)	<0.001
7–8 h/day	117,987 (69.09)	117,759 (69.11)	228 (57.00)	
Social contact				
Infrequent	22,748 (13.32)	22,658 (13.30)	90 (22.50)	<0.001
Frequent	148,035 (86.68)	147,725 (86.70)	310 (77.50)	
Sedentary behavior				
High	46,758 (27.38)	46,606 (27.35)	152 (38.00)	<0.001
Low	124,025 (72.62)	123,777 (72.65)	248 (62.00)	

^a^Categorical variables are presented as number (percentage) and compared using the chi-square test.

### Identification of the healthy lifestyle-related metabolomic signature

**Fig. 2:**
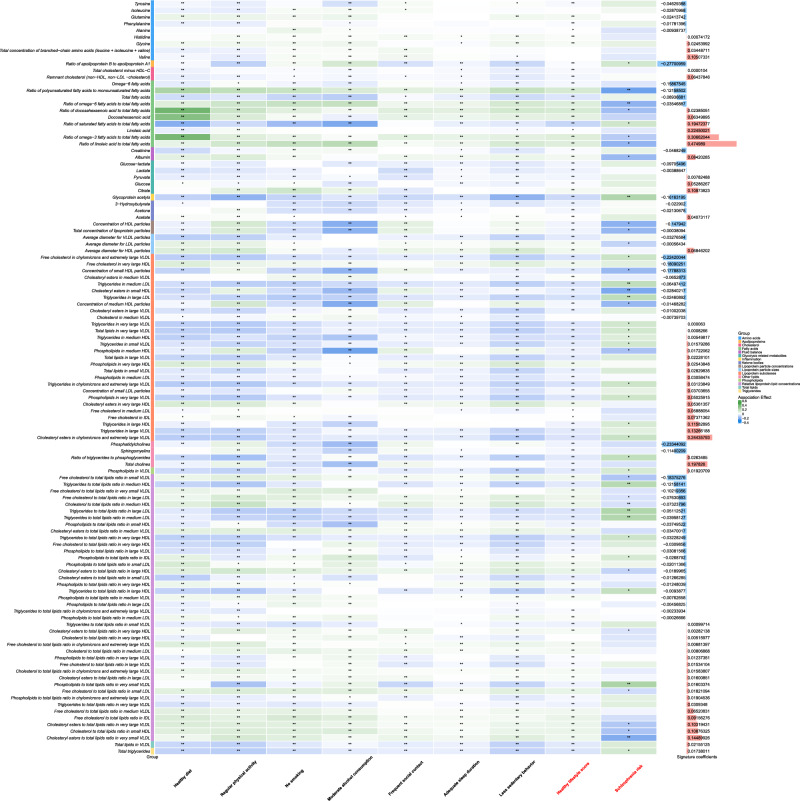
Associations of the 113 metabolites constituting the metabolomic signature with the healthy lifestyle score, each healthy lifestyle component, and schizophrenia risk. Displayed from left to right are the associations of each metabolite with individual healthy lifestyle components, the overall healthy lifestyle score, and subsequent schizophrenia risk, as well as the coefficients (weights) of each metabolite within the metabolomic signature. Red indicates positive coefficients, whereas blue indicates negative coefficients. For associations with the healthy lifestyle score and its components, beta coefficients represent the standard deviation (SD) changes in metabolite levels per 1-point increase in the lifestyle score. For associations with schizophrenia risk, beta coefficients represent the natural logarithm of the hazard ratio (ln[HR]) per 1-SD increase in metabolite levels. Asterisks denote levels of statistical significance: * *P* < 0.05 and ** Bonferroni-corrected *P* < 0.05. C cholesterol, HDL high-density lipoprotein, IDL intermediate-density lipoprotein, LDL low-density lipoprotein, VLDL very-low-density lipoprotein.

Among the 251 metabolites, the majority were found to be significantly associated with the baseline healthy lifestyle score, and the results from the first repeat assessment showed high consistency with the baseline findings (Supplementary Table 6). The correlation matrix revealed a high degree of intercorrelation among the 251 metabolites (Supplementary Fig. 1). Using elastic net regression on the 251 metabolites measured at baseline, a total of 113 metabolites were selected to construct the overall metabolomic signature of the healthy lifestyle. The metabolites included in this signature were primarily related to lipoprotein lipid concentrations (35.40%), lipoprotein subclasses (24.78%), fatty acids (8.85%), amino acids (7.96%), glycolysis-related metabolites (4.42%), and other lipids (3.54%), covering a total of 16 metabolite categories (Supplementary Fig. 2A). Furthermore, the metabolomic signature showed a significant positive correlation with the healthy lifestyle score (baseline: *r* = 0.36, *P* < 0.001; repeat assessment: *r* = 0.34, *P* < 0.001; Supplementary Fig. 2B, C). Notably, the largest positive contributors to the metabolomic signature were primarily ratio of linoleic acid (LA) to total fatty acids, ratio of omega-3 fatty acids to total fatty acids, cholesteryl esters in chylomicrons, extremely large very-low-density lipoprotein (VLDL), LA, and total cholines. In contrast, the main negative contributors included ratio of apolipoprotein B to apolipoprotein A1 (ApoB/ApoA1), phosphatidylcholines, free cholesterol in chylomicrons and extremely large VLDL, free cholesterol to total lipids ratio in small VLDL and free cholesterol in very large high-density lipoprotein (HDL) (Fig. 2). The PCA of the 113 selected metabolites identified PC1 as capturing the largest proportion of variance (30.6%). The top 20 metabolites contributing most strongly to PC1 are shown in Supplementary Table 7. These metabolites were primarily associated with triglycerides and total lipid content in VLDL subclasses, as well as relative lipoprotein lipid composition and fatty acid profiles (e.g., polyunsaturated to monounsaturated fatty acid ratio [PUFAs/MUFAs] and omega-6 fatty acids).

### Associations of the healthy lifestyle score with schizophrenia risk

Our analysis showed that higher healthy lifestyle scores were linked to a lower risk of schizophrenia, with the association demonstrating a dose–response pattern (Supplementary Fig. 3A). In the fully adjusted models of the main analysis, adherence to a healthy lifestyle was associated with a reduced risk of schizophrenia. Per 1-point increase in the healthy lifestyle score, the HR for schizophrenia was 0.81 (95% CI: 0.75–0.87) (Table 2). Compared with participants with an unhealthy lifestyle, those with a moderately healthy lifestyle and a healthy lifestyle had a reduced risk of schizophrenia, with HRs of 0.58 (95% CI: 0.43–0.77) and 0.42 (95% CI: 0.29–0.61), respectively. These associations were further supported by the Kaplan–Meier survival curves, which showed a lower cumulative incidence of schizophrenia with increasingly healthier lifestyles (Supplementary Fig. 4A). Specifically, we found that frequent social contact, adequate sleep duration, no smoking, less sedentary behavior, and regular physical activity were each inversely associated with the risk of schizophrenia, with HRs (95% CIs) of 0.62 (0.49–0.79), 0.68 (0.56–0.84), 0.75 (0.61–0.91), 0.75 (0.61–0.93), and 0.78 (0.62–0.98), respectively. However, the results indicated that, according to our current definitions, moderate alcohol consumption and a healthy diet may not be associated with a lower risk of schizophrenia (Supplementary Table 8).

**Table 2:** Associations between the healthy lifestyle score, the metabolomic signature, and the risk of incident schizophrenia.

Variable	Age- and sex-adjusted model		Multivariable-adjusted model^a^		Multivariable-adjusted + mutual adjustment ^b^	
	HR (95% CI)	*P* value	HR (95% CI)	*P* value	HR (95% CI)	*P* value
Healthy Lifestyle Score						
Unhealthy	Ref.		Ref.		Ref.	
Moderately healthy	0.45 (0.34, 0.60)	<0.001	0.52 (0.39, 0.69)	<0.001	0.58 (0.43, 0.77)	<0.001
Healthy	0.27 (0.19, 0.39)	<0.001	0.35 (0.25, 0.51)	<0.001	0.42 (0.29, 0.61)	<0.001
Per 1-point increase (continuous)	0.74 (0.69, 0.79)	<0.001	0.77 (0.72, 0.83)	<0.001	0.81 (0.75, 0.87)	<0.001
Metabolomic signature						
Low	Ref.		Ref.		Ref.	
Intermediate	0.54 (0.43, 0.67)	<0.001	0.59 (0.47, 0.74)	<0.001	0.64 (0.51, 0.81)	<0.001
High	0.38 (0.28, 0.53)	<0.001	0.41 (0.29, 0.58)	<0.001	0.48 (0.33, 0.69)	<0.001
Per 1-SD increase (continuous)	0.49 (0.40, 0.59)	<0.001	0.52 (0.41, 0.64)	<0.001	0.62 (0.49, 0.79)	<0.001

*HR* hazard ratio, *CI* confidence interval.

^a^Adjusted for age, sex, ethnicity, Townsend deprivation index, education level, body mass index (BMI), and a range of medication use, including antidiabetic, antihypertensive, and lipid-lowering drugs.

^b^Further adjusted for both the healthy lifestyle score and the metabolomic signature simultaneously in the multivariable model to assess their independent associations.

### Associations of the metabolites, metabolomic signature, with schizophrenia risk

Several metabolites contributing to the metabolomic signature were significantly associated with the risk of schizophrenia. These included triglycerides to total lipids ratio in large low-density lipoprotein (LDL), triglycerides to total lipids ratio in medium LDL, PUFAs/MUFAs, glycoprotein acetyls (GlycA), cholesteryl esters in small HDL, and phospholipids to total lipids ratio in very small VLDL, with corresponding HRs (95% CIs) of 1.25 (1.13–1.37), 1.24 (1.13–1.36), 0.79 (0.71–0.88), 1.24 (1.12–1.38), 0.81 (0.73–0.89), and 1.26 (1.13–1.40) respectively (Supplementary Table 9). Furthermore, after full adjustment, the metabolomic signature was inversely associated with the risk of incident schizophrenia, with an HR of 0.62 (95% CI: 0.49–0.79) per 1-SD increase (Table 2). In the stratified analysis, participants with moderate and high metabolomic signatures had lower risks of schizophrenia, with HRs (95% CIs) of 0.64 (0.51, 0.81) and 0.48 (0.33, 0.69), respectively, compared with those in the low metabolomic signature group. Our findings suggest that a favorable metabolic profile is associated with a reduced risk of schizophrenia, with evidence of a dose–response relationship (Supplementary Fig. 3B). Kaplan–Meier analysis also showed that participants with favorable metabolomic signatures had a lower risk of developing schizophrenia compared with those with unfavorable metabolomic signatures (Supplementary Fig. 4B). The 10-year cumulative incidence of schizophrenia was 0.28% in the low metabolomic signature group, 0.15% in the intermediate group, and 0.10% in the high group, corresponding to an absolute risk difference of 0.18% between the extreme groups.

**Fig. 3:**
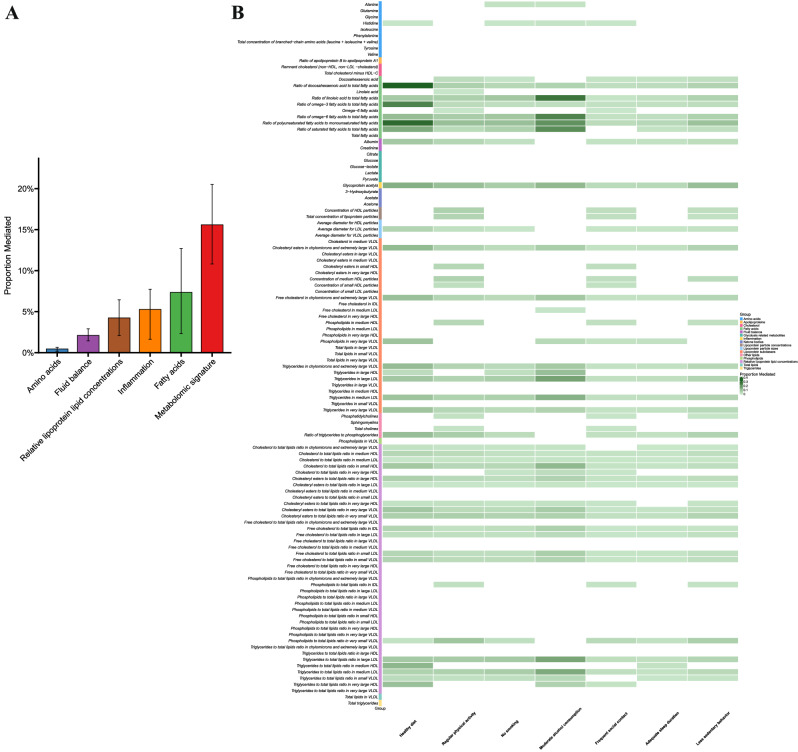
Proportion mediated by metabolites or metabolomic signature in the association between lifestyle and schizophrenia risk. **A** Proportion mediated by the metabolomic signature or metabolic pathways in the association between the lifestyle score and schizophrenia risk. **B** Proportion mediated by individual metabolites constituting the metabolomic signature in the association between specific lifestyle factors and schizophrenia risk. In (**B**) areas of the heatmap corresponding to metabolites with non-significant mediation proportions are left blank. C cholesterol, HDL high-density lipoprotein, IDL intermediate-density lipoprotein, LDL low-density lipoprotein, VLDL very-low-density lipoprotein.

Our analysis indicated that 15.59% of the beneficial effect of a healthy lifestyle on schizophrenia risk was mediated through the metabolomic signature (Fig. 3A). This mediation effect was primarily attributed to three metabolic pathways: fatty acids, inflammation, and relative lipoprotein lipid concentrations, with mediation proportions (95% CI) of 7.35% (2.33–12.70%), 5.27% (1.63–7.73%), and 4.23% (2.08–6.43%), respectively.

Upon examining the mediation proportion of each individual metabolite, we found that GlycA, several lipoprotein subclasses, several fatty acids and several relative lipoprotein lipid concentrations mediated the associations between all individual lifestyle factors and the risk of schizophrenia (Fig. 3B). Notably, the reduced risk of schizophrenia associated with a healthy dietary pattern may be significantly mediated by ratio of docosahexaenoic acid (DHA) to total fatty acids, PUFAs/MUFAs, and ratio of omega-3 fatty acids to total fatty acids. Similarly, the inverse association between moderate alcohol consumption and schizophrenia risk may be significantly mediated by ratio of LA to total fatty acids, ratio of omega-6 fatty acids to total fatty acids, ratio of saturated fatty acids (SFAs) to total fatty acids, and PUFAs/MUFAs.

Our GWAS identified 34 independent lead SNPs associated with the metabolomic signature (Supplementary Table 10). Using these SNPs as instruments, two-sample MR analysis indicated a potential causal effect of the genetically predicted metabolomic signature on schizophrenia risk, with the IVW estimate showing an odds ratio (OR) of 0.88 (95% CI: 0.80–0.98). Sensitivity analyses, including MR-Egger and weighted median methods, produced broadly consistent results. There was no strong evidence for heterogeneity (Cochran’s *Q* = 30.68, *P* = 0.583) or horizontal pleiotropy (Egger intercept = –0.0019, *P* = 0.274). These findings support a role of the genetically predicted metabolomic signature in influencing schizophrenia risk, but do not directly establish causality for lifestyle-modifiable exposures (Supplementary Table 11). Sensitivity analysis using leave-one-out analysis suggested that no single SNP drove the observed MR association (Supplementary Fig. 5).

### Subgroup analyses and sensitivity analyses

The sensitivity analyses yielded consistent results across different age (<50 years, 50–60 years or ≥60 years) and sex (female or male) subgroups (Supplementary Table 12). In addition to age and sex, sensitivity analyses were conducted for subgroups based on baseline variables showing significant differences (ethnicity, socioeconomic status, education, and medication use) and schizophrenia PRS (above vs below median). As shown in Supplementary Table 13, the association between the metabolomic signature and incident schizophrenia was generally consistent across all subgroups, with no significant evidence of effect modification (all *P* for interaction >0.05). For the healthy lifestyle score, most subgroups also showed no significant interaction; however, a significant interaction was observed for Townsend deprivation index (*P* for interaction <0.001), indicating that the protective association of a healthy lifestyle may vary by socioeconomic status.

In the sensitivity analyses, the associations between the metabolomic signature and the risk of schizophrenia remained robust after excluding participants who developed schizophrenia within the first 2 or 5 years of follow-up (Supplementary Table 14), excluding those with baseline all-cause dementia or organic psychosis (Supplementary Table 15), additionally adjusting for history of cancer and cardiovascular disease (Supplementary Table 16), imputing missing lifestyle data (Supplementary Table 17), and performing competing-risk analyses using the Fine–Gray model treating death as a competing event (Supplementary Table 18). The findings were also consistent when alternative definitions of schizophrenia outcomes were applied (Supplementary Table 19). Similar associations were observed when the metabolomic signature was analyzed in quintiles (Q1–Q5), with a significant trend across categories (Supplementary Table 20). In sensitivity analyses, the mediation results remained generally consistent (Supplementary Table 21), with total effects, natural direct and indirect effects, and proportion of effect mediated showing similar magnitude and direction. These findings support the robustness of the observed mediation by the metabolomic signature.

## Discussion

Using UK Biobank NMR metabolomics data, we identified 113 metabolites associated with healthy lifestyle and derived a metabolomic signature associated with reduced schizophrenia risk. Mediation analyses suggested that this signature partly accounted for the association between lifestyle and disease risk. MR analyses further provided genetic support for a potential causal relationship between the metabolomic signature and schizophrenia.

Previous studies have reported associations between various metabolic biomarkers and individual lifestyle factors, including smoking, alcohol consumption, diet, physical activity, sleep, sedentary behavior, and social isolation. Smoking has been linked to decreased fatty acid unsaturation, increased VLDL particle size and concentration, elevated triglyceride levels across multiple lipoprotein subclasses, reduced cholesterol content in HDL, and lower cholesterol levels in intermediate-density lipoprotein (IDL)^38^. Alcohol consumption has been associated with significant alterations in lipoprotein subclasses and fatty acid profiles^39,40^. Dietary patterns have been shown to commonly influence fatty acid and amino acid metabolism^41–43^. In particular, a healthy diet has been associated with increased fatty acid unsaturation and elevated proportions and concentrations of PUFAs, including omega-3 (notably DHA) and omega-6 (notably LA)^44^. Physical activity was associated with a favorable metabolic profile, characterized by lower VLDL and LDL levels, higher concentrations of large HDL particles, and reduced GlycA levels, whereas sedentary behavior demonstrated the opposite pattern^45^. Lipid and fatty acid metabolites, including total cholines and LA, have been strongly linked to healthy sleep^33^. Additionally, Hao Xiang et al. developed a metabolomic signature for social isolation, comprising 28 metabolites related to inflammation, amino acids, ketone bodies, and fatty acids^46^.

The neuroprotective effects of PUFAs and their metabolites have been extensively studied, revealing their ability to inhibit the production of pro-inflammatory cytokines^47^. Dietary patterns rich in omega-3 fatty acids were believed to modulate neuroinflammation and may contribute to the development of schizophrenia^48–50^. Notably, omega-3 fatty acids have been shown not only to improve schizophrenia symptoms but also to exert a beneficial regulatory effect on serum triglyceride levels^49^. Schizophrenia is consistently associated with alterations in the plasma lipidome, and improvements in lipid profiles have been closely linked to the disorder’s pathophysiology^51^. Adjunctive statin therapy has demonstrated potential in alleviating both negative and positive symptoms in affected individuals^52–54^. A critical insight into schizophrenia pathophysiology is the presence of inflammation in patients^55^. Clinical trials have confirmed that lifestyle interventions can significantly improve lipid profiles and reduce inflammation—a finding well documented in other conditions such as type 2 diabetes, hypertension, and coronary heart disease^56–58^.

In our study, we simultaneously examined associations between multiple lifestyle factors and NMR-based metabolites, identifying 113 metabolic biomarkers linked to seven healthy lifestyle behaviors. Principal component analysis revealed that PC1 captured a major axis of metabolic variation, with the strongest contributions from triglyceride-rich lipoproteins, particularly VLDL subclasses, their lipid composition, and fatty acid profiles (e.g., PUFA/MUFA ratio and omega-6 fatty acids). This pattern suggests that the dominant metabolic variation observed may reflect alterations in systemic lipid transport and fatty acid metabolism, consistent with prior evidence^18^. Abnormal lipid metabolism has been increasingly implicated in schizophrenia pathophysiology through its potential effects on neuronal function, oxidative stress, and neuroinflammatory processes^59–61^. In particular, dysregulated VLDL metabolism may contribute to systemic inflammatory activation and cognitive dysfunction, potentially influencing neuroimmune signaling pathways^62^. PUFAs, which are highly enriched in neuronal membranes, play essential roles in synaptic function, membrane integrity, and inflammatory regulation^63,64^. Omega-3 PUFAs, in particular, exert anti-inflammatory and pro-resolving effects via lipid-derived mediators and have been shown to influence endothelial function and neurovascular homeostasis^65^. Importantly, PUFA metabolism has also been linked to the regulation of blood–brain barrier (BBB) integrity. Experimental and clinical evidence suggests that omega-3 PUFAs can modulate tight junction proteins, endothelial activation, and matrix metalloproteinase activity, thereby reducing BBB permeability and limiting neuroinflammatory signaling^66,67^. As a dynamic neuroimmune interface, the BBB both restricts and coordinates communication between peripheral immune processes and central nervous system function, providing a biologically plausible pathway through which systemic metabolic disturbances may influence brain-related outcomes^68^. Notably, the lifestyle-related metabolomic signature identified in our study was associated with a lower risk of schizophrenia, accounting for 15.59% of the total effect. Fatty acids, inflammatory pathways, and relative lipoprotein lipid composition emerged as key contributors to this mediation process. Together, these findings support the hypothesis that lifestyle-related metabolic alterations—particularly those involving triglyceride-rich lipoproteins and fatty acid metabolism—may represent an important biological interface linking health behaviors, systemic inflammation, and central nervous system dysfunction in schizophrenia.

Schizophrenia is increasingly recognized as a biologically heterogeneous disorder, and growing evidence highlights the importance of identifying biologically informed subtypes to advance mechanistic understanding and therapeutic development^69^. In this context, peripheral metabolomic alterations may provide complementary insights into systemic biological processes that interact with central nervous system dysfunction. Emerging studies suggest that metabolic dysregulation is associated with neurobiological abnormalities, including structural and functional brain alterations as well as variability in treatment response^70–72^. Importantly, second-generation antipsychotics (e.g., olanzapine and risperidone) have been consistently linked to adverse metabolic effects in individuals with schizophrenia, and unhealthy lifestyle factors may further compound treatment-related metabolic disturbances^73–76^. Together, these findings underscore the need to consider metabolic pathways as a potential interface linking disease heterogeneity, treatment effects, and clinical outcomes. Future research integrating longitudinal metabolomic profiling with neuroimaging, genetic data, and detailed pharmacological information may help clarify the biological mechanisms underlying schizophrenia heterogeneity and support the development of precision psychiatry approaches.

To the best of our knowledge, this study provides novel evidence on potential metabolic pathways underlying the reduced risk of schizophrenia associated with a healthy lifestyle. However, several limitations should be acknowledged. First, lifestyle behaviors were assessed based on self-reported data, which may be subject to recall bias. Nevertheless, previous research has demonstrated good concordance between self-reported data and primary care records within the UK Biobank, supporting the reliability of the information collected^77^. Second, for most participants, plasma metabolomics measurements were only taken once at baseline, which limited our ability to observe longitudinal metabolic changes and to investigate critical time windows in which metabolomic signatures may affect schizophrenia risk. Third, the overall healthy lifestyle score was unweighted, which may not accurately represent the independent contributions of each behavior. Fourth, both the derivation of the metabolomic signature and the evaluation of its association with schizophrenia risk were conducted within the same cohort, which may introduce potential optimism bias. Although independent external validation would be ideal, the relatively limited number of schizophrenia cases in our study may reduce the stability of estimates if the dataset were split into separate training and testing subsets. To mitigate potential overfitting, we used elastic net regression with a 10-fold cross-validation procedure and applied the one-standard-error rule to select a parsimonious model. Nevertheless, the findings should be interpreted with caution and require validation in independent cohorts. Fifth, given the relatively limited number of incident schizophrenia cases, mediation estimates should be interpreted cautiously, although sensitivity analyses yielded broadly consistent results. Finally, this study was conducted in a generally healthier middle-aged and older European population, which may lead to lower absolute event rates; therefore, caution is warranted when generalizing these findings to other age groups or ethnic populations. In addition, subtle changes in lifestyle behaviors at baseline could reflect prodromal symptoms of schizophrenia, introducing the possibility of reverse causality, which should be considered when interpreting the observed associations.

In summary, using individual-level data from the UK Biobank, we identified a novel metabolomic signature of a healthy lifestyle and demonstrated its potential causal link to late-onset schizophrenia risk. These findings may contribute to improved risk stratification and the development of targeted preventive strategies focusing on lifestyle-related metabolic pathways in schizophrenia.

## Supplementary information

Supplementary Material 1

Supplementary Material 2 (PDF)

Supplementary Material 2 (Excel)

## Data Availability

The data supporting the findings of this study are available from the UK Biobank (https://www.ukbiobank.ac.uk/) under application number 452,018. Access to the raw data requires approval from the UK Biobank.

## References

[1] Lehman, A. F. et al. Practice guideline for the treatment of patients with schizophrenia, second edition. *Am. J. Psychiatry.* **161**, 1–56 (2004). 15000267

[2] Laursen, T. M., Nordentoft, M. & Mortensen, P. B. Excess early mortality in schizophrenia. *Annu. Rev. Clin. Psychol.* **10**, 425–448 (2014). 24313570 10.1146/annurev-clinpsy-032813-153657

[3] Howard, R., Rabins, P. V., Seeman, M. V. & Jeste, D. V. Late-onset schizophrenia and very-late-onset schizophrenia-like psychosis: an international consensus. *Int. Late-Onset Schizophrenia Group. Am. J. Psychiatry* **157**, 172–178 (2000). 10.1176/appi.ajp.157.2.172 10671383

[4] van Os, J., Kenis, G. & Rutten, B. P. The environment and schizophrenia. *Nature* **468**, 203–212 (2010). 21068828 10.1038/nature09563

[5] Stilo, S. A. & Murray, R. M. Non-genetic factors in schizophrenia. *Curr. Psychiatry Rep.* **21**, 100 (2019). 31522306 10.1007/s11920-019-1091-3 PMC6745031

[6] Cougnard, A. et al. Does normal developmental expression of psychosis combine with environmental risk to cause persistence of psychosis? A psychosis proneness-persistence model. *Psychol. Med.* **37**, 513–527 (2007). 17288646 10.1017/S0033291706009731

[7] Stepniak, B. et al. Accumulated environmental risk determining age at schizophrenia onset: a deep phenotyping-based study. *Lancet Psychiatry.* **1**, 444–453 (2014). 26361199 10.1016/S2215-0366(14)70379-7

[8] Padmanabhan, J. L., Shah, J. L., Tandon, N. & Keshavan, M. S. The “polyenviromic risk score”: Aggregating environmental risk factors predicts conversion to psychosis in familial high-risk subjects. *Schizophr. Res.* **181**, 17–22 (2017). 28029515 10.1016/j.schres.2016.10.014 PMC5365360

[9] Firth, J. et al. A meta-review of “lifestyle psychiatry”: the role of exercise, smoking, diet and sleep in the prevention and treatment of mental disorders. *World Psychiatry* **19**, 360–380 (2020). 32931092 10.1002/wps.20773 PMC7491615

[10] Gurillo, P., Jauhar, S., Murray, R. M. & MacCabe, J. H. Does tobacco use cause psychosis? Systematic review and meta-analysis. *Lancet Psychiatry* **2**, 718–725 (2015). 26249303 10.1016/S2215-0366(15)00152-2 PMC4698800

[11] Wootton, R. E. et al. Evidence for causal effects of lifetime smoking on risk for depression and schizophrenia: a Mendelian randomisation study. *Psychol. Med.* **50**, 2435–2443 (2020). 31689377 10.1017/S0033291719002678 PMC7610182

[12] Gage, S. H. et al. Investigating causality in associations between smoking initiation and schizophrenia using Mendelian randomization. *Sci. Rep.* **7**, 40653 (2017). 28102331 10.1038/srep40653 PMC5244403

[13] Brokmeier, L. L. et al. Does physical activity reduce the risk of psychosis? A systematic review and meta-analysis of prospective studies. *Psychiatry Res.* **284**, 112675 (2020). 31757637 10.1016/j.psychres.2019.112675

[14] Kirli, U., Binbay, T., Alptekin, K., Kayahan, B. & Elbi, H. The relationship between alcohol-cannabis use and stressful events with the development of incident clinical psychosis in a community-based prospective cohort. *Turk. Psikiyatr. Derg.* **32**, 235–245 (2021). 10.5080/u26410 34964097

[15] Andreu-Bernabeu, Á et al. Polygenic contribution to the relationship of loneliness and social isolation with schizophrenia. *Nat. Commun.* **13**, 51 (2022). 35013163 10.1038/s41467-021-27598-6 PMC8748758

[16] Hoang, T. D. et al. Effect of early adult patterns of physical activity and television viewing on midlife cognitive function. *JAMA Psychiatry* **73**, 73–79 (2016). 26629780 10.1001/jamapsychiatry.2015.2468 PMC4755299

[17] Raichlen, D. A. et al. Sedentary behavior and incident dementia among older adults. *Jama* **330**, 934–940 (2023). 37698563 10.1001/jama.2023.15231 PMC10498332

[18] Tian, F. et al. Plasma metabolomic signature of healthy lifestyle, structural brain reserve and risk of dementia. *Brain.* **148**, 143–153 (2025). 39324695 10.1093/brain/awae257

[19] Szoke, A. et al. Multimorbidity and the etiology of schizophrenia. *Curr. Psychiatry Rep.* **26**, 253–263 (2024). 38625632 10.1007/s11920-024-01500-9

[20] Jahrami, H. A., Faris, M. A. E., Saif, Z. Q. & Hammad, L. H. Assessing dietary and lifestyle risk factors and their associations with disease comorbidities among patients with schizophrenia: a case-control study from Bahrain. *Asian J. Psychiatr.* **28**, 115–123 (2017). 28784363 10.1016/j.ajp.2017.03.036

[21] Tolstikov, V., Moser, A. J., Sarangarajan, R., Narain, N. R. & Kiebish, M. A. Current status of metabolomic biomarker discovery: impact of study design and demographic characteristics. *Metabolites.* **10**, 10.3390/metabo10060224 (2020). 10.3390/metabo10060224 PMC7345110 32485899

[22] Delgado-Velandia, M. et al. Healthy lifestyle, metabolomics and incident type 2 diabetes in a population-based cohort from Spain. *Int J. Behav. Nutr. Phys. Act.* **19**, 8 (2022). 35086546 10.1186/s12966-021-01219-3 PMC8793258

[23] Wang, Y. et al. Healthy lifestyle, metabolic signature, and risk of cardiovascular diseases: a population-based study. *Nutrients.* **16**, 10.3390/nu16203553 (2024). 10.3390/nu16203553 PMC11510148 39458547

[24] Fernandez, C. et al. Plasma lipidome and prediction of type 2 diabetes in the population-based Malmö diet and cancer cohort. *Diab. Care* **43**, 366–373 (2020). 10.2337/dc19-1199 31818810

[25] Sudlow, C. et al. UK Biobank: an open access resource for identifying the causes of a wide range of complex diseases of middle and old age. *PLoS Med.* **12**, e1001779 (2015). 25826379 10.1371/journal.pmed.1001779 PMC4380465

[26] Han, H. et al. Association of a healthy lifestyle with all-cause and cause-specific mortality among individuals with type 2 diabetes: a prospective study in UK Biobank. *Diab. Care* **45**, 319–329 (2022). 10.2337/dc21-1512 34857534

[27] Wang, B. et al. Association of combined healthy lifestyle factors with incident dementia in patients with type 2 diabetes. *Neurology.* **99**, e2336–e2345 (2022). 36104282 10.1212/WNL.0000000000201231

[28] Tian, F. et al. Post-cardiovascular disease healthy lifestyle, inflammation and metabolic biomarkers, and risk of dementia: a population-based longitudinal study. *Am. J. Clin. Nutr.* **121**, 511–521 (2025). 40044393 10.1016/j.ajcnut.2024.09.012

[29] Zhang, Y. B. et al. Associations of healthy lifestyle and socioeconomic status with mortality and incident cardiovascular disease: two prospective cohort studies. *Bmj* **373**, n604 (2021). 33853828 10.1136/bmj.n604 PMC8044922

[30] Navratilova, H. F., Lanham-New, S., Whetton, A. D. & Geifman, N. Associations of diet with health outcomes in the UK Biobank: a systematic review. *Nutrients* **16**, 10.3390/nu16040523 (2024). 10.3390/nu16040523 PMC10892867 38398847

[31] Smith, R. W. et al. Social isolation and risk of heart disease and stroke: analysis of two large UK prospective studies. *Lancet Public Health* **6**, e232–e239 (2021). 33662329 10.1016/S2468-2667(20)30291-7 PMC7994247

[32] Julkunen, H., Cichońska, A., Slagboom, P. E. & Würtz, P. Metabolic biomarker profiling for identification of susceptibility to severe pneumonia and COVID-19 in the general population. *Elife* **10**, 10.7554/eLife.63033 (2021). 10.7554/eLife.63033 PMC8172246 33942721

[33] Zhuang, Z. et al. Sleep patterns, plasma metabolome, and risk of incident type 2 diabetes mellitus. *J. Clin. Endocrinol. Metab.* **108**, e1034–e1043 (2023). 37084357 10.1210/clinem/dgad218

[34] Friedman, J., Hastie, T. & Tibshirani, R. Regularization paths for generalized linear models via coordinate descent. *J. Stat. Softw.* **33**, 1–22 (2010). 20808728 PMC2929880

[35] Shi, B., Choirat, C., Coull, B. A., VanderWeele, T. J. & Valeri, L. CMAverse: a suite of functions for reproducible causal mediation analyses. *Epidemiology* **32**, e20–e22 (2021). 34028370 10.1097/EDE.0000000000001378

[36] Pedersen, E. M. et al. ADuLT: An efficient and robust time-to-event GWAS. *Nat. Commun.* **14**, 5553 (2023). 37689771 10.1038/s41467-023-41210-z PMC10492844

[37] Didelez, V. & Sheehan, N. Mendelian randomization as an instrumental variable approach to causal inference. *Stat. Methods Med. Res.* **16**, 309–330 (2007). 17715159 10.1177/0962280206077743

[38] Wei, Y. et al. Metabolic profiling of smoking, associations with type 2 diabetes and interaction with genetic susceptibility. *Eur. J. Epidemiol.* **39**, 667–678 (2024). 38555549 10.1007/s10654-024-01117-5 PMC11249521

[39] Würtz, P. et al. Metabolic profiling of alcohol consumption in 9778 young adults. *Int. J. Epidemiol.* **45**, 1493–1506 (2016). 27494945 10.1093/ije/dyw175 PMC5100616

[40] Muth, N. D., Laughlin, G. A., von Mühlen, D., Smith, S. C. & Barrett-Connor, E. High-density lipoprotein subclasses are a potential intermediary between alcohol intake and reduced risk of cardiovascular disease: the Rancho Bernardo Study. *Br. J. Nutr.* **104**, 1034–1042 (2010). 20426890 10.1017/S0007114510001595

[41] Wang, F. et al. Plasma metabolite profiles related to plant-based diets and the risk of type 2 diabetes. *Diabetologia* **65**, 1119–1132 (2022). 35391539 10.1007/s00125-022-05692-8 PMC9810389

[42] Li, J. et al. The Mediterranean diet, plasma metabolome, and cardiovascular disease risk. *Eur. Heart J.* **41**, 2645–2656 (2020). 32406924 10.1093/eurheartj/ehaa209 PMC7377580

[43] Esko, T. et al. Metabolomic profiles as reliable biomarkers of dietary composition. *Am. J. Clin. Nutr.* **105**, 547–554 (2017). 28077380 10.3945/ajcn.116.144428 PMC5320413

[44] Akbaraly, T. et al. Association of circulating metabolites with healthy diet and risk of cardiovascular disease: analysis of two cohort studies. *Sci. Rep.* **8**, 8620 (2018). 29872056 10.1038/s41598-018-26441-1 PMC5988716

[45] Pang, Y. et al. Physical activity, sedentary leisure time, circulating metabolic markers, and risk of major vascular diseases. *Circ. Genom. Precis Med.* **12**, 386–396 (2019). 31461308 10.1161/CIRCGEN.118.002527 PMC6752700

[46] Xiang, H. et al. Association of social isolation and plasma metabolites with the risk of venous thromboembolism. *Arterioscler. Thromb. Vasc. Biol.* **45**, 332–340 (2025). 39723538 10.1161/ATVBAHA.124.322112

[47] Das, U. N. Polyunsaturated fatty acids and their metabolites in the pathobiology of schizophrenia. *Prog. Neuropsychopharmacol. Biol. Psychiatry* **42**, 122–134 (2013). 22735394 10.1016/j.pnpbp.2012.06.010

[48] Kurowska, A., Ziemichód, W., Herbet, M. & Piątkowska-Chmiel, I. The role of diet as a modulator of the inflammatory process in the neurological diseases. *Nutrients* **15**, 10.3390/nu15061436 (2023). 10.3390/nu15061436 PMC10057655 36986165

[49] Goh, K. K., Chen, C. Y., Chen, C. H. & Lu, M. L. Effects of omega-3 polyunsaturated fatty acids supplements on psychopathology and metabolic parameters in schizophrenia: a meta-analysis of randomized controlled trials. *J. Psychopharmacol.* **35**, 221–235 (2021). 33586517 10.1177/0269881120981392

[50] Rarinca, V. et al. Relevance of diet in schizophrenia: a review focusing on prenatal nutritional deficiency, obesity, oxidative stress and inflammation. *Front. Nutr.* **11**, 1497569 (2024). 39734678 10.3389/fnut.2024.1497569 PMC11673491

[51] Tkachev, A. et al. Lipid alteration signature in the blood plasma of individuals with schizophrenia, depression, and bipolar disorder. *JAMA Psychiatry* **80**, 250–259 (2023). 36696101 10.1001/jamapsychiatry.2022.4350 PMC9878436

[52] Tajik-Esmaeeli, S. et al. Simvastatin adjunct therapy for negative symptoms of schizophrenia: a randomized double-blind placebo-controlled trial. *Int. Clin. Psychopharmacol.* **32**, 87–94 (2017). 27941358 10.1097/YIC.0000000000000159

[53] Vincenzi, B. et al. A randomized placebo-controlled pilot study of pravastatin as an adjunctive therapy in schizophrenia patients: effect on inflammation, psychopathology, cognition and lipid metabolism. *Schizophr. Res.* **159**, 395–403 (2014). 25261882 10.1016/j.schres.2014.08.021 PMC4311769

[54] Shen, H. et al. Adjunctive therapy with statins in schizophrenia patients: a meta-analysis and implications. *Psychiatry Res.* **262**, 84–93 (2018). 29427912 10.1016/j.psychres.2018.02.018

[55] Murphy, C. E., Walker, A. K. & Weickert, C. S. Neuroinflammation in schizophrenia: the role of nuclear factor kappa B. *Transl. Psychiatry* **11**, 528 (2021). 34650030 10.1038/s41398-021-01607-0 PMC8516884

[56] Belalcazar, L. M. et al. A 1-year lifestyle intervention for weight loss in individuals with type 2 diabetes reduces high C-reactive protein levels and identifies metabolic predictors of change: from the Look AHEAD (Action for Health in Diabetes) study. *Diab. Care* **33**, 2297–2303 (2010). 10.2337/dc10-0728 PMC2963483 20682679

[57] Valenzuela, P. L. et al. Lifestyle interventions for the prevention and treatment of hypertension. *Nat. Rev. Cardiol.* **18**, 251–275 (2021). 33037326 10.1038/s41569-020-00437-9

[58] Ornish, D. et al. Intensive lifestyle changes for reversal of coronary heart disease. *Jama* **280**, 2001–2007 (1998). 9863851 10.1001/jama.280.23.2001

[59] Zhao, X., Zhang, S., Sanders, A. R. & Duan, J. Brain lipids and lipid droplet dysregulation in Alzheimer’s disease and neuropsychiatric disorders. *Complex Psychiatry* **9**, 154–171 (2023). 38058955 10.1159/000535131 PMC10697751

[60] Yeo, I. J. et al. Overexpression of transmembrane TNFα in brain endothelial cells induces schizophrenia-relevant behaviors. *Mol. Psychiatry* **28**, 843–855 (2023). 36333582 10.1038/s41380-022-01846-7

[61] González-Castro, T. B. et al. Effects of IL-6/IL-6R axis alterations in serum, plasma and cerebrospinal fluid with the schizophrenia: an updated review and meta-analysis of 58 studies. *Mol. Cell Biochem.* **479**, 525–537 (2024). 37103677 10.1007/s11010-023-04747-7

[62] Huang, J. K. & Lee, H. C. Emerging evidence of pathological roles of very-low-density lipoprotein (VLDL). *Int. J. Mol. Sci.* **23**, 10.3390/ijms23084300 (2022). 10.3390/ijms23084300 PMC9031540 35457118

[63] Bazinet, R. P. & Layé, S. Polyunsaturated fatty acids and their metabolites in brain function and disease. *Nat. Rev. Neurosci.* **15**, 771–785 (2014). 25387473 10.1038/nrn3820

[64] Janssen, C. I. & Kiliaan, A. J. Long-chain polyunsaturated fatty acids (LCPUFA) from genesis to senescence: the influence of LCPUFA on neural development, aging, and neurodegeneration. *Prog. Lipid Res* **53**, 1–17 (2014). 24334113 10.1016/j.plipres.2013.10.002

[65] Devassy, J. G., Leng, S., Gabbs, M., Monirujjaman, M. & Aukema, H. M. Omega-3 polyunsaturated fatty acids and oxylipins in neuroinflammation and management of Alzheimer disease. *Adv. Nutr.* **7**, 905–916 (2016). 27633106 10.3945/an.116.012187 PMC5015035

[66] Navarro, C. et al. Influence of polyunsaturated fatty acids on Cortisol transport through MDCK and MDCK-MDR1 cells as blood-brain barrier in vitro model. *Eur. J. Pharm. Sci.* **42**, 290–299 (2011). 21182940 10.1016/j.ejps.2010.12.005

[67] Wen, J. et al. Unraveling the impact of Omega-3 polyunsaturated fatty acids on blood-brain barrier (BBB) integrity and glymphatic function. *Brain Behav. Immun.* **115**, 335–355 (2024). 37914102 10.1016/j.bbi.2023.10.018

[68] Banks, W. A. The blood-brain barrier in neuroimmunology: tales of separation and assimilation. *Brain Behav. Immun.* **44**, 1–8 (2015). 25172555 10.1016/j.bbi.2014.08.007 PMC4275374

[69] Zhang, W. J., Sweeney, J. A., Bishop, J. R., Gong, Q. Y. & Lui, S. Biological subtyping of psychiatric syndromes as a pathway for advances in drug discovery and personalized medicine. *NATURE MENTAL HEALTH* **1**, 88–99 (2023).

[70] Du, W., Tang, B. Q., Liu, S. H., Zhang, W. J. & Lui, S. Causal associations between iron levels in subcortical brain regions and psychiatric disorders: a Mendelian randomization study. *Transl. Psychiatry* **15**, 10.1038/s41398-025-03231-8 (2025). 10.1038/s41398-025-03231-8 PMC11754438 39843424

[71] Zhang, W., Qiu, C. & Lui, S. Imaging biomarker studies of antipsychotic-naïve first-episode schizophrenia in China: progress and future directions. *Schizophr. Bull.* **51**, 379–391 (2025). 39841545 10.1093/schbul/sbaf002 PMC11908865

[72] Paul, T. et al. Neurostructural changes in schizophrenia and treatment-resistance: a narrative review. *Psychoradiology* **4**, kkae015 (2024). 39399446 10.1093/psyrad/kkae015 PMC11467815

[73] Li, R. et al. Effects of olanzapine treatment on lipid profiles in patients with schizophrenia: a systematic review and meta-analysis. *Sci. Rep.* **10**, 17028 (2020). 33046806 10.1038/s41598-020-73983-4 PMC7552389

[74] Dai, S. et al. A comprehensive metabolomic and lipidomic study of olanzapine in the treatment of first-episode schizophrenia. *Asian J. Psychiatr.* **105**, 104387 (2025). 40015078 10.1016/j.ajp.2025.104387

[75] Aquino, A. et al. Blood-based lipidomics approach to evaluate biomarkers associated with response to olanzapine, risperidone, and quetiapine treatment in schizophrenia patients. *Front. Psychiatry* **9**, 209 (2018). 29887809 10.3389/fpsyt.2018.00209 PMC5982405

[76] Meyer, J. M. & Correll, C. U. Increased metabolic potential, efficacy, and safety of emerging treatments in schizophrenia. *CNS Drugs* **37**, 545–570 (2023). 37470979 10.1007/s40263-023-01022-7 PMC10374807

[77] Darke, P. et al. Curating a longitudinal research resource using linked primary care EHR data-a UK Biobank case study. *J. Am. Med. Inf. Assoc.* **29**, 546–552 (2022). 10.1093/jamia/ocab260 PMC8800530 34897458

