# Supplementary material for: Healthy lifestyle, metabolomic signature, and risk of late-onset schizophrenia: evidence from the prospective cohort
Source: Schizophrenia (Heidelb). 2026 Apr 14;12(1):50. doi: 10.1038/s41537-026-00752-z (PMC13270109; doi:10.1038/s41537-026-00752-z)
Supplement: Supplementary file 1 — Supplementary Material 1 [file 41537_2026_752_MOESM1_ESM.pdf]

Supplementary Materials

Supplementary Figure 1. Correlation matrix of all 251 metabolic biomarkers. Warmer colors (red) indicate stronger positive correlations, whereas cooler colors (blue) indicate stronger negative correlations. — *Page 1*

Supplementary Figure 2. Composition of the metabolomic signature and its correlation with the healthy lifestyle score. (A) The metabolomic signature comprises 113 metabolites identified through elastic net regression, in which the healthy lifestyle score was regressed on a total of 251 metabolites. (B) and (C) show the correlations between the healthy lifestyle score and the metabolomic signature at baseline and at the first follow-up assessment, respectively. — *Page 2*

Supplementary Figure 3. Dose-response relationships between (A) the healthy lifestyle score, (B) the metabolomic signature, and the risk of schizophrenia. Models were adjusted for age, sex, ethnicity, Townsend deprivation index, education level, body mass index (BMI), and a range of medication use, including antidiabetic, antihypertensive, and lipid-lowering drugs. Additionally, both the healthy lifestyle score and the metabolomic signature were included simultaneously in the model to assess their independent associations. — *Page 3*

Supplementary Figure 4. Cumulative incidence of schizophrenia estimated separately for (A) healthy lifestyle score groups and (B) metabolomic signature groups. — *Page 4*

Supplementary Figure 5. Leave-one-out analysis of Mendelian randomization for the association between the metabolomic signature and schizophrenia risk. — *Page 5*

Supplementary Table 1. Definitions and assessment methods for lifestyle factors in the UK Biobank. — *Page 6*

Supplementary Table 2. Definitions of dietary components in the UK Biobank. — *Page 7*

Supplementary Table 3. Information of 251 circulating metabolites in the UK Biobank. — *Page 8*

Supplementary Table 4. Summary statistics of baseline age in the UK Biobank, stratified by incident schizophrenia status. — *Page 9*

Supplementary Table 5. The characteristics of the participants at first repeat assessment. — *Page 10*

Supplementary Table 6. Distribution of metabolites and associations of the healthy lifestyle score with the metabolites at baseline and first repeat assessment visit. — *Page 11*

Supplementary Table 7. Top 20 metabolites contributing to PC1 in principal component analysis. — *Page 12*

Supplementary Table 8. Associations of individual healthy lifestyle components with the risk of schizophrenia. — *Page 13*

Supplementary Table 9. Associations of the 113 metabolites comprised the metabolomic signature with healthy lifestyle components, healthy lifestyle scores, and the risk of schizophrSupplementary Table 15. Associations of the healthy lifestyle score and metabolomic signature with schizophrenia risk: sensitivity

analyses excluding baseline dementia or organic psychosis.enia. (Figure 2 source data) — *Page 14*

Supplementary Table 10. Thirty-four single nucleotide polymorphisms (SNPs) for the metabolomic signature used as instrumental variables in Mendelian randomization. — *Page 15*

Supplementary Table 11. Results of Mendelian randomization analyses examining the metabolomic signature as the exposure and schizophrenia as the outcome. — *Page 16*

Supplementary Table 12. Associations between the metabolomic signature and risk of incident schizophrenia, stratified by age and sex. — *Page 17*

Supplementary Table 13. Associations between the metabolomic signature and risk of incident schizophrenia, stratified by ethnicity, socioeconomic status, education, medication use, and polygenic risk score. — *Page 18*

Supplementary Table 14. Associations of the healthy lifestyle score and the metabolomic signature with the risk of incident schizophrenia after excluding events occurring within the first two years of follow-up. — *Page 19*

Supplementary Table 15. Associations of the healthy lifestyle score and the metabolomic signature with the risk of incident schizophrenia after excluding baseline dementia or organic psychosis. — *Page 20*

Supplementary Table 16. Associations of the healthy lifestyle score and the metabolomic signature with the risk of incident schizophrenia after further adjusting history of cancer and cardiovascular disease. — *Page 21*

Supplementary Table 17. Associations of the healthy lifestyle score and the metabolomic signature with the risk of incident schizophrenia after interpolating missing lifestyle data. — *Page 22*

Supplementary Table 18. Associations of the healthy lifestyle score and the metabolomic signature with the risk of incident schizophrenia using a competing-risk model (Fine–Gray). — *Page 23*

Supplementary Table 19. Associations of the healthy lifestyle score and the metabolomic signature with the risk of incident schizophrenia using alternative outcome definitions. — *Page 24*

Supplementary Table 20. Associations between metabolomic signature quintiles (Q1–Q5) and risk of incident schizophrenia. — *Page 25*

Supplementary Table 21. Sensitivity analyses of the mediation effect of the metabolomic signature on the association between healthy lifestyle score and incident schizophrenia. — *Page 26*

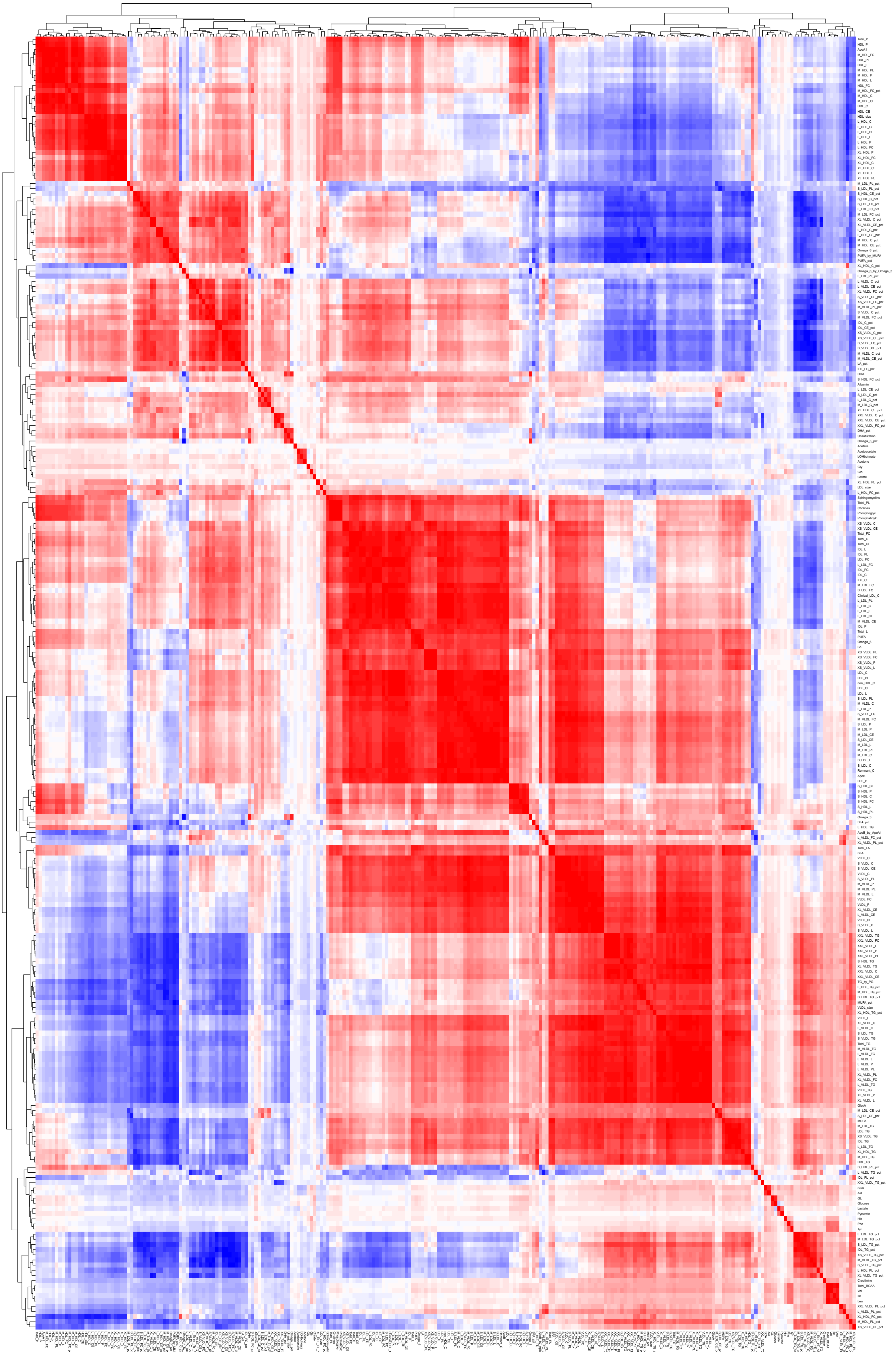

Supplementary Figure 1. Correlation matrix of all 251 metabolic biomarkers. Warmer colors (red) indicate stronger positive correlations, whereas cooler colors (blue) indicate stronger negative correlations.

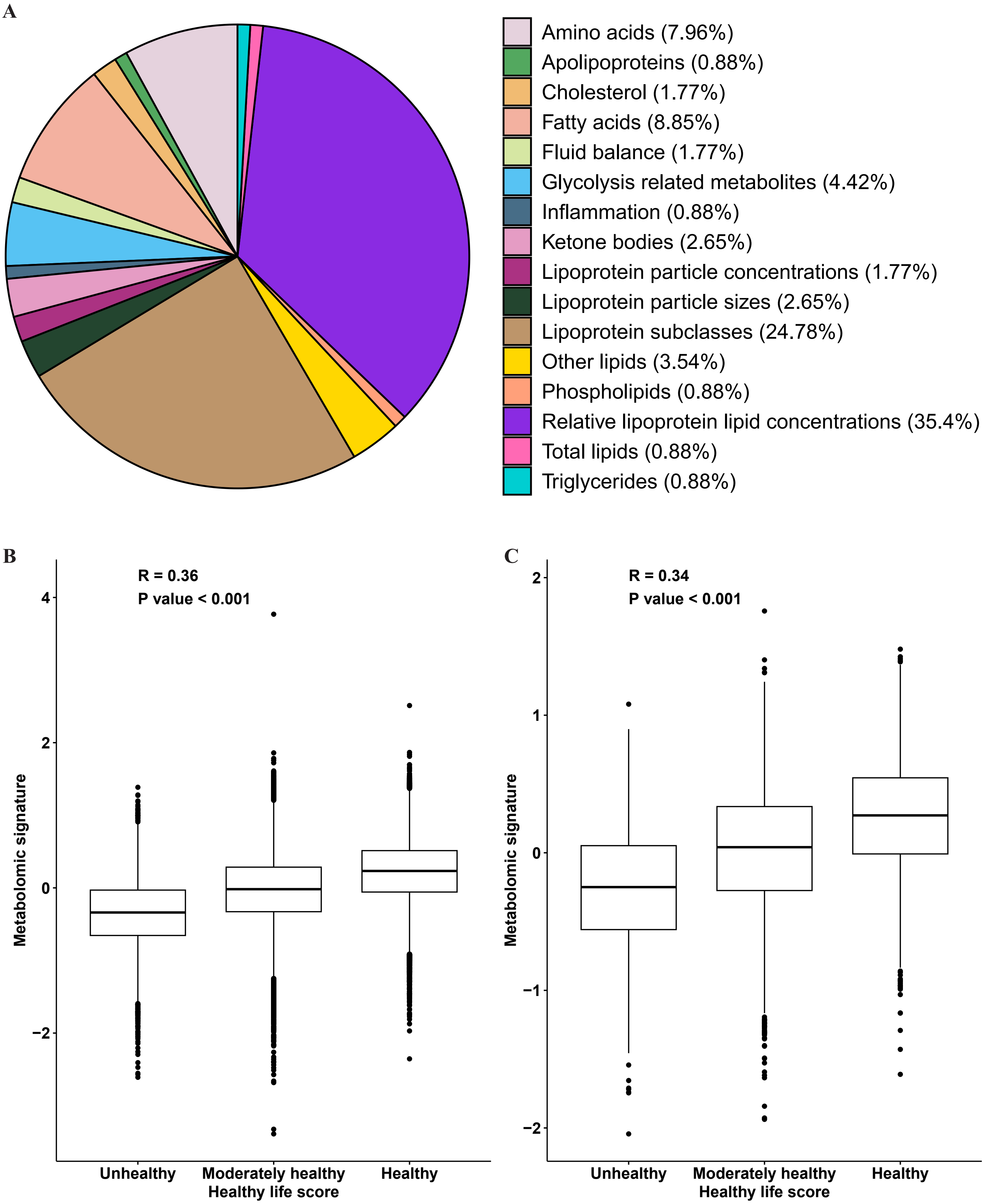

Supplementary Figure 2. Composition of the metabolomic signature and its correlation with the healthy lifestyle score. **(A)** The metabolomic signature comprises 113 metabolites identified through elastic net regression, in which the healthy lifestyle score was regressed on a total of 251 metabolites. **(B)** and **(C)** show the correlations between the healthy lifestyle score and the metabolomic signature at baseline and at the first follow-up assessment, respectively.

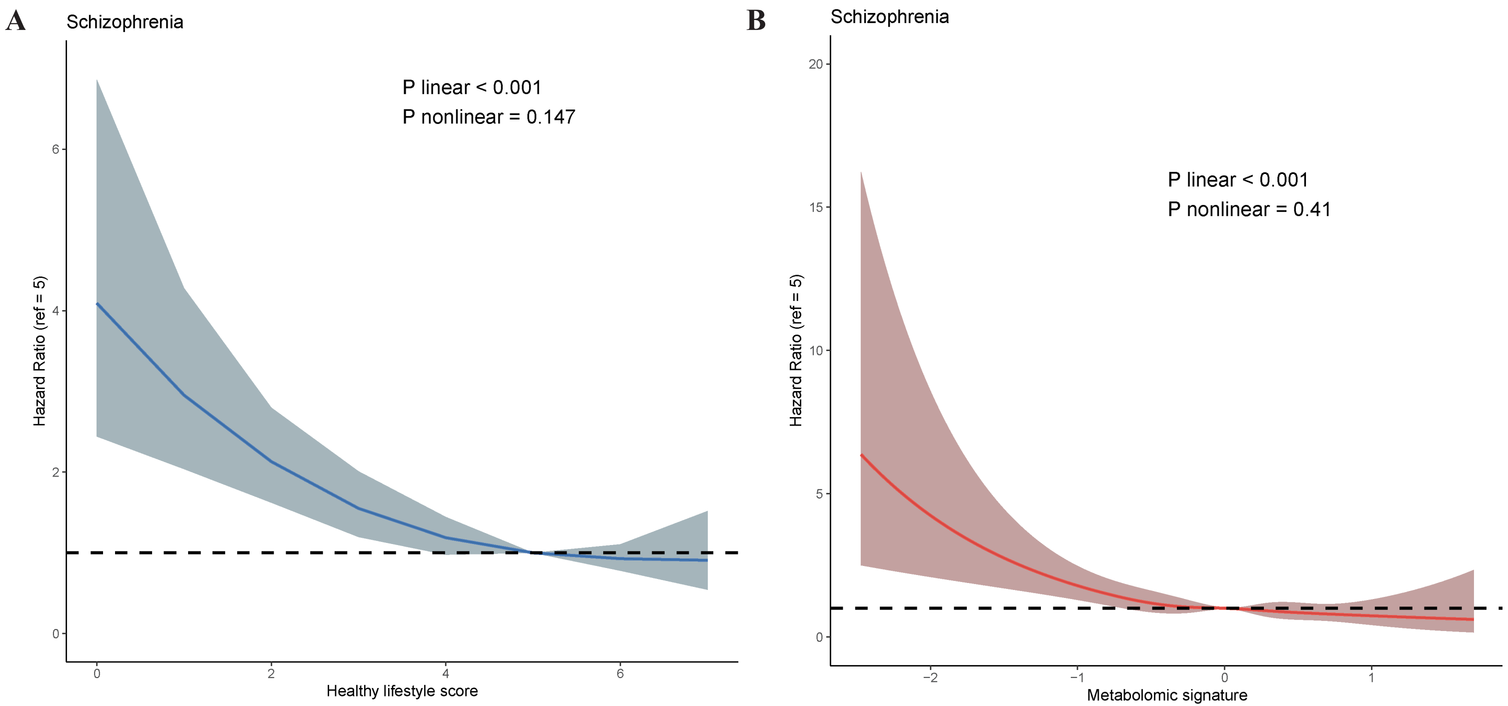

Supplementary Figure 3. Dose-response relationships between **(A)** the healthy lifestyle score, **(B)** the metabolomic signature, and the risk of schizophrenia. Models were adjusted for age, sex, ethnicity, Townsend deprivation index, education level, body mass index (BMI), and a range of medication use, including antidiabetic, antihypertensive, and lipid-lowering drugs. Additionally, both the healthy lifestyle score and the metabolomic signature were included simultaneously in the model to assess their independent associations.

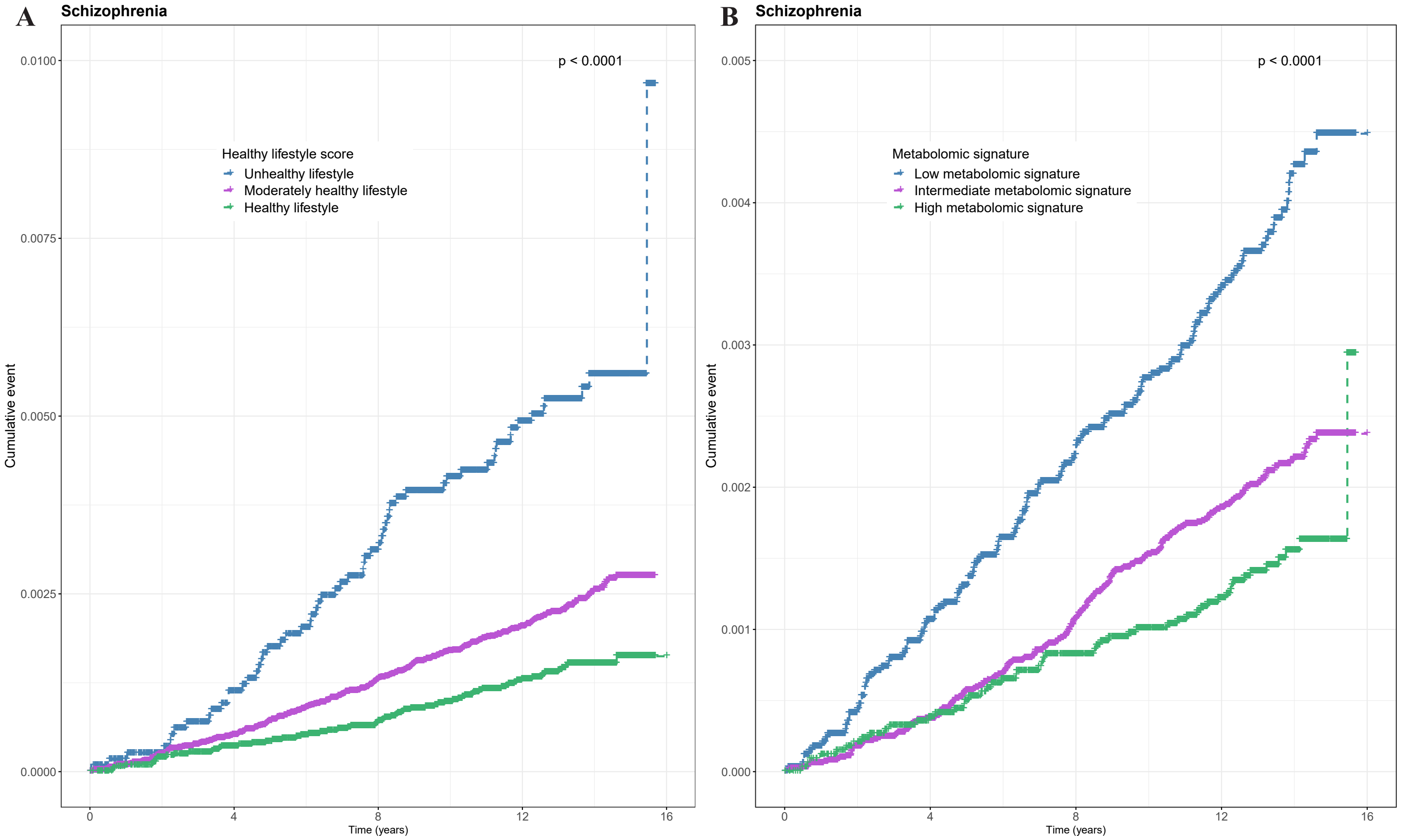

Supplementary Figure 4. Cumulative incidence of schizophrenia estimated separately for **(A)** healthy lifestyle score groups and **(B)** metabolomic signature groups.

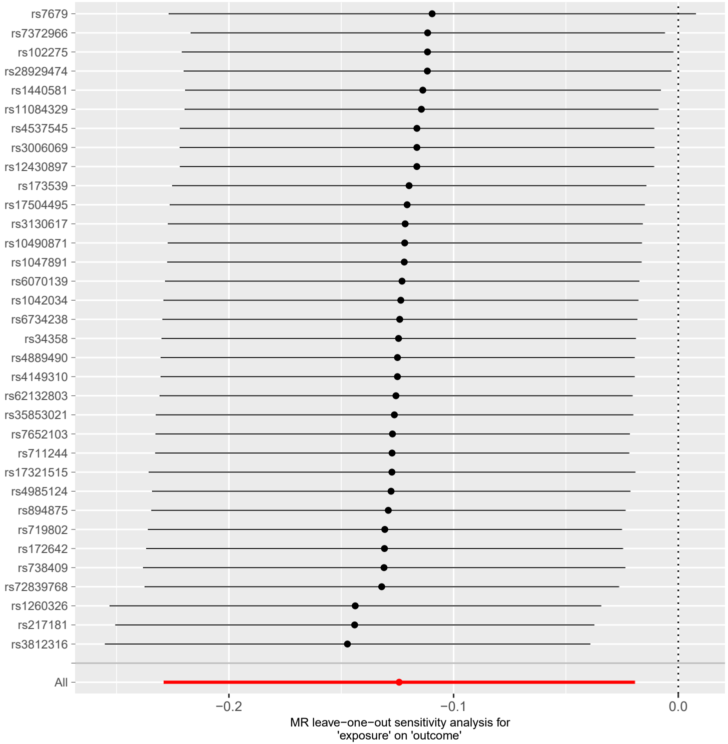

Supplementary Figure 5. Leave-one-out analysis of Mendelian randomization for the association between the metabolomic signature and schizophrenia risk.

Supplementary Table 1. Definitions and assessment methods for lifestyle factors in the UK Biobank.

**Lifestyle factors**	**Definition or method of measurement**	**Healthy behavior (1 point)**	**Unhealthy behavior (0 point)**
Smoking	Self-reported current/past smoking status of the participants.	Never smoking.	Previous/current smoking.
Alcohol consumption	Based on participants' self-reported drinking frequency and their average weekly or monthly consumption of red wine (glasses), champagne and white wine (glasses), beer and cider (pints), spirits (measures), fortified wine (glasses), and other types of alcoholic beverages (glasses), the average daily alcohol intake was calculated for each participant.	Healthy drinking as consumption of <1 drink/day (women), <2 drinks /day (men).	Unhealthy drinking as consumption of ≥1 drinks/day (women), ≥2 drinks/day (men), or no alcohol consumption.
Physical activity	Based on participants' self-reported number of days engaging in moderate or vigorous physical activity per week and the average duration on a typical day, the total weekly duration of moderate or vigorous physical activity was calculated for each participant.	Regular physical activity as ≥150 min/week of moderate activity, ≥75 min/week of vigorous activity, or equivalent combination.	Irregular physical activity as <150 min/week of moderate activity, <75 min/week of vigorous activity, or not meeting equivalent combination.
Diet	Based on participants' self-reported dietary data from the Oxford WebQ, a web-based 24-hour dietary recall questionnaire, and following previous UK Biobank studies, a diet score was constructed using seven food components: fruits, vegetables, fish, whole grains (higher intake considered beneficial), and red meat, processed products, and refined grains (lower intake considered beneficial). Details are provided in Supplementary Table 2.	Diet score ≥4.	Diet score <4.
Sleep duration	Self-reported sleep duration in every 24 hours.	Sleep duration of 7–8 h/day.	Sleep duration <7 h/day or sleep duration >8 h/day.
Sedentary behavior	Self-reported time spent watching television in a typical day.	Time spent watching television <4 h/day.	Time spent watching television ≥4 h/day.
Social contact	A social contact score was constructed based on three self-reported questions. The first question asked about the number of people living in the participant's household, with one point assigned to those living alone. The second question assessed the frequency of visiting or being visited by friends or family, with one point assigned to those reporting less than once per month. The third question inquired about participation in group activities, such as sports, gym, pub, social club, religious group, or adult education classes, with one point assigned to those not engaging in any of these activities on a weekly basis. The total social contact score was calculated by summing the points from these three items.	Social contact score <2.	Social contact score ≥2.

Supplementary Table 2. Definitions of dietary components in the UK Biobank.

Components	Field ID	Description	Intake goal *
Fruit	1309	Fresh fruit intake (pieces/day)	≥3 servings/day
	1319	Dried fruit intake (pieces/day)	
Vegetable	1299	Salad / raw vegetable intake (tablespoons/day)	≥3 servings/day
	1289	Cooked vegetable intake (tablespoons/day)	
Whole grains	1438 & 1448	Bread intake (whole meal or wholegrain, slices/week)	≥3 servings/day
	1458 & 1468	Cereal intake (bran cereal, oat cereal, or muesli, bowls/week)	
Fish	1329	Oily fish intake (times/week)	≥2 servings/week
	1339	Non-oily fish intake (times/week)	
Refined grains	1438 & 1448	Bread intake (white, brown, or other type of bread, slices/week)	≤2 servings/day
	1458 & 1468	Cereal intake (biscuit cereal or other, bowls/week)	
Processed meats	1349 & 3680	Processed meat intake (0 times/week if never eaten meat)	≤1 serving/week
Unprocessed meats	1369 & 1379 & 1389 & 3680	Beef, lamb/mutton or pork intake (0 times/week if never eaten meat)	≤2 serving/week

* If participants achieved the intake target for a given dietary component, one point was assigned to the diet score.

Supplementary Table 3. Information of 251 circulating metabolites in the UK Biobank.

The detailed data information could be found in supplementary Excel table.

C, cholesterol; HDL, high-density lipoprotein; IDL, intermediate-density lipoprotein; LDL, low-density lipoprotein; VLDL, very-low-density lipoprotein.

Supplementary Table 4. Summary statistics of baseline age in the UK Biobank, stratified by incident schizophrenia status.

	N	Min	Q1.25%	Median	Q3.75%	Max	Mean	SD
Total	170783	38	50	58	63	73	56.4	8.07
Schizophrenia	400	40	49.75	58	64	69	56.75	8.56
Non-schizophrenia	170383	38	50	58	63	73	56.4	8.07

Standard deviation, SD.

Supplementary Table 5. The characteristics of the participants at first repeat assessment.

Characteristics *	At baseline (n=170,783)	First repeat assessment visit (n=9,750)
**Age**		
<50 years	40696 (23.83)	1824 (18.71)
50–60 years	57113 (33.44)	3561 (36.52)
≥60 years	72974 (42.73)	4365 (44.77)
**Male**	85891 (50.29)	5318 (54.54)
**White**	163714 (95.86)	9550 (97.95)
**Townsend deprivation index (above median)**	80374 (47.06)	3842 (39.41)
**College**	60748 (35.57)	4547 (46.64)
**Body mass index (≥18.5 & ≤24.9, kg/m^2)**	117276 (68.67)	6319 (64.81)
**Glucose lowering drug use**	5491 (3.22)	262 (2.69)
**Blood pressure medication use**	34354 (20.12)	1772 (18.17)
**Lipid lowering medication use**	29569 (17.31)	1595 (16.36)
**Healthy lifestyle scores**		
Healthy	45478 (26.63)	3006 (30.83)
Moderately healthy	113607 (66.52)	6276 (64.37)
Unhealthy	11698 (6.85)	468 (4.80)
**Lifestyle components**		
**Drinking**		
Excess	86024 (50.37)	4643 (47.62)
Moderate	84759 (49.63)	5107 (52.38)
**Diet**		
Unideal	93258 (54.61)	5125 (52.56)
Ideal	77525 (45.39)	4625 (47.44)
**Physical activity**		
Lacking	32230 (18.87)	1826 (18.73)
Regular	138553 (81.13)	7924 (81.27)
**Smoking**		
Previous/Current	77719 (45.51)	4118 (42.24)
Never	93064 (54.49)	5632 (57.76)
**Sleep duration**		
<7 h/day or >8 h/day	52796 (30.91)	2711 (27.81)
7–8 h/day	117987 (69.09)	7039 (72.19)
**Social contact**		
Infrequent	22748 (13.32)	1177 (12.07)
Frequent	148035 (86.68)	8573 (87.93)
**Sedentary behavior**		
High	46758 (27.38)	2131 (21.86)
Low	124025 (72.62)	7619 (78.14)

* Categorical variables are presented as number (percentage).

Supplementary Table 6. Distribution of metabolites and associations of the healthy lifestyle score with the metabolites at baseline and first repeat assessment visit.

The detailed data information could be found in supplementary Excel table.

† Beta coefficients represent the standard deviation (SD) change in metabolite levels per 1-point increase in the healthy lifestyle score. Adjusted for age, sex, ethnicity, Townsend deprivation index, education level, body mass index (BMI), and a range of medication use, including antidiabetic, antihypertensive, and lipid-lowering drugs.

* *P* values were Bonferroni-corrected.

SE, standard error; C, cholesterol; HDL, high-density lipoprotein; IDL, intermediate-density lipoprotein; LDL, low-density lipoprotein; VLDL, very-low-density lipoprotein.

Supplementary Table 7. Top 20 metabolites contributing to PC1 in principal component analysis.

Metabolic biomarkers	Description	Group	PC1 loading
Total_TG	Total triglycerides	Triglycerides	-0.16455
XL_VLDL_TG	Triglycerides in very large VLDL	Lipoprotein subclasses	-0.16311
XL_VLDL_L	Total lipids in very large VLDL	Lipoprotein subclasses	-0.16206
S_VLDL_TG	Triglycerides in small VLDL	Lipoprotein subclasses	-0.15985
XL_VLDL_PL	Phospholipids in very large VLDL	Lipoprotein subclasses	-0.15924
TG_by_PG	Ratio of triglycerides to phosphoglycerides	Other lipids	-0.15892
XXL_VLDL_FC	Free cholesterol in chylomicrons and extremely large VLDL	Lipoprotein subclasses	-0.15721
XXL_VLDL_CE	Cholesteryl esters in chylomicrons and extremely large VLDL	Lipoprotein subclasses	-0.15675
L_VLDL_L	Total lipids in large VLDL	Lipoprotein subclasses	-0.15622
L_VLDL_TG	Triglycerides in large VLDL	Lipoprotein subclasses	-0.15504
XXL_VLDL_TG	Triglycerides in chylomicrons and extremely large VLDL	Lipoprotein subclasses	-0.15356
XL_HDL_TG_pct	Triglycerides to total lipids ratio in very large HDL	Relative lipoprotein lipid concentrations	-0.15304
PUFA_by_MUFA	Ratio of polyunsaturated fatty acids to monounsaturated fatty acids	Fatty acids	0.152057
M_HDL_TG_pct	Triglycerides to total lipids ratio in medium HDL	Relative lipoprotein lipid concentrations	-0.15161
VLDL_size	Average diameter for VLDL particles	Lipoprotein particle sizes	-0.15035
VLDL_L	Total lipids in VLDL	Total lipids	-0.15013
L_HDL_TG_pct	Triglycerides to total lipids ratio in large HDL	Relative lipoprotein lipid concentrations	-0.14986
Omega_6_pct	Ratio of omega-6 fatty acids to total fatty acids	Fatty acids	0.144498
M_HDL_C_pct	Cholesterol to total lipids ratio in medium HDL	Relative lipoprotein lipid concentrations	0.144328
M_LDL_TG	Triglycerides in medium LDL	Lipoprotein subclasses	-0.14392

C, cholesterol; HDL, high-density lipoprotein; IDL, intermediate-density lipoprotein; LDL, low-density lipoprotein; VLDL, very-low-density lipoprotein.

Supplementary Table 8. Associations of individual healthy lifestyle components with the risk of schizophrenia.

**Healthy lifestyle components**	**Model 1 †**		**Model 2 ***	
	**HR (95% CI)**	***P* value**	**HR (95% CI)**	***P* value**
Frequent social contact	0.57 (0.45, 0.72)	<0.001	0.62 (0.49, 0.79)	<0.001
Adequate sleep duration	0.65 (0.53, 0.80)	<0.001	0.68 (0.56, 0.84)	<0.001
No smoking	0.72 (0.59, 0.88)	0.001	0.75 (0.61, 0.91)	0.005
Less sedentary behavior	0.69 (0.56, 0.86)	<0.001	0.75 (0.61, 0.93)	0.010
Regular physical activity	0.69 (0.55, 0.87)	0.002	0.78 (0.62, 0.98)	0.030
Healthy diet	0.84 (0.69, 1.03)	0.102	0.89 (0.73, 1.10)	0.280
Moderate alcohol consumption	0.88 (0.72, 1.08)	0.231	0.95 (0.78, 1.16)	0.620

† Model 1 adjusted for age, sex, ethnicity, Townsend deprivation index, education level, body mass index (BMI), and a range of medication use, including antidiabetic, antihypertensive, and lipid-lowering drugs.
* Model 2 further adjusted for the remaining six healthy lifestyle components.
HR, hazard ratio; CI, confidence interval.

Supplementary Table 9. Associations of the 113 metabolites comprised the metabolomic signature with healthy lifestyle components, healthy lifestyle scores, and the risk of schizophrenia. (Figure 2 source data)

The detailed data information could be found in supplementary Excel table.

† Beta coefficients represent the standard deviation (SD) change in metabolite levels per 1-point increase in the healthy lifestyle score. Adjusted for age, sex, ethnicity, Townsend deprivation index, education level, body mass index (BMI), and a range of medication use, including antidiabetic, antihypertensive, and lipid-lowering drugs.

* *P* values were Bonferroni-corrected.

SE, standard error; C, cholesterol; HDL, high-density lipoprotein; IDL, intermediate-density lipoprotein; LDL, low-density lipoprotein; VLDL, very-low-density lipoprotein.

Supplementary Table 10. Thirty-four single nucleotide polymorphisms (SNPs) for the metabolomic signature used as instrumental variables in Mendelian randomization.

SNP	Beta	SE	*P* value	Effect allele	Other allele	Eaf
rs102275	-0.0249747	0.00169623	4.92863E-49	C	T	0.355295
rs1042034	-0.0128886	0.00202114	1.81264E-10	C	T	0.20277
rs1047891	0.0104023	0.00175018	2.79541E-09	A	C	0.31587
rs10490871	-0.00928168	0.00168387	3.55129E-08	G	A	0.367219
rs11084329	0.010333	0.00172335	2.02896E-09	C	T	0.336407
rs12430897	-0.0106519	0.00191487	2.66023E-08	A	G	0.235729
rs1260326	-0.0246841	0.00166415	9.77098E-50	T	C	0.392562
rs1440581	-0.0111762	0.00163168	7.43868E-12	T	C	0.467309
rs172642	-0.0129418	0.00163116	2.13451E-15	A	C	0.468597
rs17321515	0.0209149	0.00162883	1.0203E-37	G	A	0.474585
rs173539	0.00956397	0.00172779	3.11097E-08	T	C	0.331831
rs17504495	-0.0124146	0.00191662	9.36333E-11	C	T	0.236801
rs217181	-0.018343	0.00205488	4.4448E-19	T	C	0.194735
rs28929474	0.0673852	0.0058344	7.64609E-31	T	C	0.0198497
rs3006069	0.0101669	0.00163497	5.03513E-10	G	A	0.459095
rs3130617	0.0114901	0.00180943	2.15798E-10	C	T	0.280341
rs34358	0.0099507	0.00171028	5.96255E-09	G	A	0.346091
rs35853021	0.0138015	0.00169867	4.51246E-16	T	G	0.357476
rs3812316	0.0300574	0.00243681	6.12764E-35	G	C	0.127827
rs4149310	0.0126711	0.00224891	1.76057E-08	T	A	0.153902
rs4537545	0.00944281	0.00164852	1.01793E-08	T	C	0.418571
rs4889490	0.00935652	0.00166629	1.96697E-08	T	G	0.39048
rs4985124	-0.0154633	0.00178151	4.00101E-18	G	T	0.296233
rs6070139	0.00945529	0.00165535	1.11904E-08	A	G	0.400518
rs62132803	-0.00983418	0.00178298	3.4821E-08	T	C	0.295711
rs6734238	-0.0103505	0.00165362	3.87797E-10	G	A	0.402844
rs711244	0.00936389	0.00164995	1.38749E-08	T	C	0.419612
rs719802	-0.00914733	0.00167093	4.39764E-08	T	C	0.385145
rs72839768	-0.0316611	0.00524891	1.62386E-09	A	G	0.0244771
rs7372966	-0.00942154	0.00162498	6.72761E-09	T	C	0.499773
rs738409	0.0220614	0.00197196	4.82275E-29	G	C	0.216347
rs7652103	-0.00935809	0.00162024	7.67637E-09	G	A	0.497341
rs7679	-0.0486647	0.00209414	3.0676E-119	C	T	0.185432
rs894875	-0.00974548	0.00171222	1.25995E-08	C	T	0.34629

SE, standard error.

Supplementary Table 11. Results of Mendelian randomization analyses examining the metabolomic signature as the exposure and schizophrenia as the outcome.

**Method**	**OR (95% CI)**	***P* value**	**Q**	**Q *P* value**	**Egger intercept**	**Egger intercept *P* value**
MR Egger	0.98 (0.79, 1.21)	0.839	30.675	0.583	-0.00190	0.274
Weighted median	0.84 (0.71, 0.99)	0.042				
Inverse variance weighted	0.88 (0.80, 0.98)	0.020				

OR, odds ratio; CI, confidence interval.

Supplementary Table 12. Associations between the metabolomic signature and risk of incident schizophrenia, stratified by age and sex.

**Subgroup**	**HR (95% CI) ***	***P* value**
**Healthy Lifestyle Score**		
**Age**		
<50 years	0.80 (0.69, 0.92)	0.002
50-60 years	0.79 (0.69, 0.90)	<0.001
≥60 years	0.75 (0.68, 0.83)	<0.001
**Sex**		
Female	0.75 (0.68, 0.83)	<0.001
Male	0.80 (0.72, 0.88)	<0.001
**Metabolomic signature**		
**Age**		
<50 years	0.51 (0.33, 0.79)	0.002
50-60 years	0.45 (0.30, 0.68)	<0.001
≥60 years	0.57 (0.41, 0.79)	<0.001
**Sex**		
Female	0.49 (0.36, 0.69)	<0.001
Male	0.54 (0.40, 0.72)	<0.001

* Adjusted for age, sex, ethnicity, Townsend deprivation index, education level, body mass index (BMI), and a range of medication use, including antidiabetic, antihypertensive, and lipid-lowering drugs.

HR, hazard ratio; CI, confidence interval.

Supplementary Table 13. Associations between the metabolomic signature and risk of incident schizophrenia, stratified by ethnicity, socioeconomic status, education, medication use, and polygenic risk score.

Subgroup	Healthy Lifestyle Score			Metabolomic signature		
	**HR (95% CI)**	***P* value**	***P* for interaction**	**HR (95% CI)**	***P* value**	***P* for interaction**
**Ethnicity**			0.21			0.781
White	0.76 (0.71, 0.82)	<0.001		0.53 (0.42, 0.66)	<0.001	
Others	0.88 (0.7, 1.11)	0.287		0.53 (0.27, 1.04)	0.064	
**Townsend deprivation index**			<0.001			0.153
Above median	0.71 (0.65, 0.78)	<0.001		0.48 (0.36, 0.62)	<0.001	
Below median	0.91 (0.8, 1.03)	0.151		0.65 (0.44, 0.96)	0.031	
**Education**			0.87			0.803
College	0.76 (0.67, 0.87)	<0.001		0.57 (0.38, 0.88)	0.01	
Others	0.78 (0.72, 0.85)	<0.001		0.52 (0.4, 0.67)	<0.001	
**Glucose lowering drug use**			0.697			0.41
Yes	0.74 (0.56, 0.99)	0.04		0.45 (0.2, 1.03)	0.059	
No	0.78 (0.72, 0.84)	<0.001		0.54 (0.43, 0.68)	<0.001	
**Blood pressure medication use**			0.938			0.379
Yes	0.77 (0.67, 0.88)	<0.001		0.58 (0.39, 0.87)	0.008	
No	0.78 (0.71, 0.84)	<0.001		0.51 (0.39, 0.66)	<0.001	
**Lipid lowering medication use**			0.902			0.753
Yes	0.77 (0.67, 0.89)	<0.001		0.53 (0.35, 0.81)	0.003	
No	0.78 (0.71, 0.84)	<0.001		0.54 (0.42, 0.7)	<0.001	
**PRS**			0.965			0.838
Above median	0.78 (0.69, 0.87)	<0.001		0.55 (0.39, 0.79)	0.001	
Below median	0.78 (0.71, 0.85)	<0.001		0.5 (0.38, 0.66)	<0.001	

* Adjusted for age, sex, ethnicity, Townsend deprivation index, education level, body mass index (BMI), and a range of medication use, including antidiabetic, antihypertensive, and lipid-lowering drugs.

HR, hazard ratio; CI, confidence interval; PRS, polygenic risk score.

Supplementary Table 14. Associations of the healthy lifestyle score and the metabolomic signature with the risk of incident schizophrenia after excluding events occurring within the first 2 or 5 years of follow-up.

Outcome definition	Variable	Age- and sex-adjusted model		Multivariable-adjusted model *		Multivariable-adjusted + mutual adjustment †	
		HR (95% CI)	*P* value	HR (95% CI)	*P* value	HR (95% CI)	*P* value
**Excluding first 2 years**							
	**Healthy Lifestyle Score**						
	Unhealthy						
	Moderately healthy	0.42 (0.32, 0.57)	<0.001	0.48 (0.36, 0.65)	<0.001	0.54 (0.4, 0.72)	<0.001
	Healthy	0.25 (0.17, 0.36)	<0.001	0.31 (0.21, 0.45)	<0.001	0.38 (0.25, 0.55)	<0.001
	Per 1-point increase (continuous)	0.72 (0.67, 0.78)	<0.001	0.76 (0.7, 0.81)	<0.001	0.79 (0.73, 0.86)	<0.001
	**Metabolomic Signature**						
	Low						
	Intermediate	0.55 (0.44, 0.69)	<0.001	0.59 (0.46, 0.74)	<0.001	0.65 (0.51, 0.83)	<0.001
	High	0.37 (0.26, 0.52)	<0.001	0.38 (0.26, 0.55)	<0.001	0.45 (0.31, 0.67)	<0.001
	Per 1-SD increase (continuous)	0.48 (0.39, 0.59)	<0.001	0.49 (0.39, 0.62)	<0.001	0.6 (0.47, 0.77)	<0.001
**Excluding first 5 years**							
	**Healthy Lifestyle Score**						
	Unhealthy						
	Moderately healthy	0.47 (0.34, 0.66)	<0.001	0.54 (0.38, 0.76)	<0.001	0.59 (0.41, 0.83)	0.003
	Healthy	0.29 (0.19, 0.44)	<0.001	0.36 (0.23, 0.55)	<0.001	0.43 (0.27, 0.66)	<0.001
	Per 1-point increase (continuous)	0.73 (0.67, 0.79)	<0.001	0.76 (0.7, 0.83)	<0.001	0.79 (0.73, 0.87)	<0.001
	**Metabolomic Signature**						
	Low						
	Intermediate	0.59 (0.45, 0.77)	<0.001	0.64 (0.49, 0.84)	0.001	0.7 (0.53, 0.93)	0.013
	High	0.39 (0.26, 0.57)	<0.001	0.41 (0.27, 0.63)	<0.001	0.48 (0.31, 0.75)	0.001
	Per 1-SD increase (continuous)	0.49 (0.38, 0.62)	<0.001	0.51 (0.39, 0.67)	<0.001	0.63 (0.48, 0.84)	0.001

* Adjusted for age, sex, ethnicity, Townsend deprivation index, education level, body mass index (BMI), and a range of medication use, including antidiabetic, antihypertensive, and lipid-lowering drugs.

† Further adjusted for both the healthy lifestyle score and the metabolomic signature simultaneously in the multivariable model to assess their independent associations.

HR, hazard ratio; CI, confidence interval.

Supplementary Table 15. Associations of the healthy lifestyle score and the metabolomic signature with the risk of incident schizophrenia after excluding baseline dementia or organic psychosis.

Variable	Age- and sex-adjusted model		Multivariable-adjusted model *		Multivariable-adjusted + mutual adjustment †	
	**HR (95% CI)**	***P* value**	**HR (95% CI)**	***P* value**	**HR (95% CI)**	***P* value**
**Healthy Lifestyle Score**						
Unhealthy	Ref.		Ref.		Ref.	
Moderately healthy	0.45 (0.34, 0.6)	<0.001	0.52 (0.39, 0.69)	<0.001	0.58 (0.43, 0.77)	<0.001
Healthy	0.27 (0.19, 0.38)	<0.001	0.35 (0.24, 0.5)	<0.001	0.42 (0.29, 0.61)	<0.001
Per 1-point increase (continuous)	0.73 (0.68, 0.79)	<0.001	0.77 (0.72, 0.83)	<0.001	0.81 (0.75, 0.87)	<0.001
**Metabolic Signature**						
Low	Ref.		Ref.		Ref.	
Intermediate	0.53 (0.43, 0.67)	<0.001	0.58 (0.46, 0.73)	<0.001	0.64 (0.51, 0.81)	<0.001
High	0.38 (0.28, 0.53)	<0.001	0.41 (0.29, 0.58)	<0.001	0.48 (0.33, 0.69)	<0.001
Per 1-SD increase (continuous)	0.48 (0.4, 0.59)	<0.001	0.51 (0.41, 0.64)	<0.001	0.62 (0.5, 0.79)	<0.001

* Adjusted for age, sex, ethnicity, Townsend deprivation index, education level, body mass index (BMI), and a range of medication use, including antidiabetic, antihypertensive, and lipid-lowering drugs.

† Further adjusted for both the healthy lifestyle score and the metabolomic signature simultaneously in the multivariable model to assess their independent associations.

HR, hazard ratio; CI, confidence interval.

Supplementary Table 16. Associations of the healthy lifestyle score and the metabolomic signature with the risk of incident schizophrenia after further adjusting history of cancer and cardiovascular disease.

**Variable**	**Age- and sex-adjusted model**		**Multivariable-adjusted model ***		**Multivariable-adjusted + mutual adjustment †**	
	**HR (95% CI)**	***P* value**	**HR (95% CI)**	***P* value**	**HR (95% CI)**	***P* value**
**Healthy Lifestyle Score**						
Unhealthy	Ref.		Ref.		Ref.	
Moderately healthy	0.45 (0.34, 0.60)	<0.001	0.53 (0.40, 0.71)	<0.001	0.59 (0.44, 0.79)	<0.001
Healthy	0.27 (0.19, 0.39)	<0.001	0.36 (0.25, 0.52)	<0.001	0.44 (0.30, 0.63)	<0.001
Per 1-point increase (continuous)	0.74 (0.69, 0.79)	<0.001	0.78 (0.72, 0.84)	<0.001	0.81 (0.75, 0.88)	<0.001
**Metabolomic signature**						
Low	Ref.		Ref.		Ref.	
Intermediate	0.54 (0.43, 0.67)	<0.001	0.59 (0.47, 0.74)	<0.001	0.65 (0.51, 0.82)	<0.001
High	0.38 (0.28, 0.53)	<0.001	0.41 (0.29, 0.59)	<0.001	0.48 (0.33, 0.69)	<0.001
Per 1-SD increase (continuous)	0.49 (0.40, 0.59)	<0.001	0.52 (0.42, 0.65)	<0.001	0.62 (0.49, 0.79)	<0.001

* Adjusted for age, sex, ethnicity, Townsend deprivation index, education level, body mass index (BMI), a range of medication use, including antidiabetic, antihypertensive, and lipid-lowering drugs, as well as history of cancer and cardiovascular disease.

† Further adjusted for both the healthy lifestyle score and the metabolomic signature simultaneously in the multivariable model to assess their independent associations.

HR, hazard ratio; CI, confidence interval.

Supplementary Table 17. Associations of the healthy lifestyle score and the metabolomic signature with the risk of incident schizophrenia after interpolating missing lifestyle data.

Variable	Age- and sex-adjusted model		Multivariable-adjusted model *		Multivariable-adjusted + mutual adjustment †	
	**HR (95% CI)**	***P* value**	**HR (95% CI)**	***P* value**	**HR (95% CI)**	***P* value**
**Healthy Lifestyle Score**						
Unhealthy	Ref.		Ref.		Ref.	
Moderately healthy	0.42 (0.34, 0.52)	<0.001	0.48 (0.39, 0.60)	<0.001	0.53 (0.43, 0.65)	<0.001
Healthy	0.24 (0.18, 0.32)	<0.001	0.30 (0.23, 0.40)	<0.001	0.36 (0.27, 0.48)	<0.001
Per 1-point increase (continuous)	0.73 (0.69, 0.77)	<0.001	0.76 (0.72, 0.81)	<0.001	0.81 (0.76, 0.85)	<0.001
**Metabolomic signature**						
Low	Ref.		Ref.		Ref.	
Intermediate	0.57 (0.48, 0.68)	<0.001	0.62 (0.52, 0.74)	<0.001	0.69 (0.57, 0.83)	<0.001
High	0.38 (0.29, 0.49)	<0.001	0.39 (0.29, 0.52)	<0.001	0.48 (0.35, 0.64)	<0.001
Per 1-SD increase (continuous)	0.45 (0.38, 0.53)	<0.001	0.46 (0.39, 0.55)	<0.001	0.56 (0.46, 0.67)	<0.001

* Adjusted for age, sex, ethnicity, Townsend deprivation index, education level, body mass index (BMI), and a range of medication use, including antidiabetic, antihypertensive, and lipid-lowering drugs.

† Further adjusted for both the healthy lifestyle score and the metabolomic signature simultaneously in the multivariable model to assess their independent associations.

HR, hazard ratio; CI, confidence interval.

Supplementary Table 18. Associations of the healthy lifestyle score and the metabolomic signature with the risk of incident schizophrenia using a competing-risk model (Fine–Gray).

Variable	Age- and sex-adjusted model		Multivariable-adjusted model *		Multivariable-adjusted + mutual adjustment †	
	SHR (95% CI)	*P* value	SHR (95% CI)	*P* value	SHR (95% CI)	*P* value
**Healthy Lifestyle Score**						
Unhealthy	Ref.		Ref.		Ref.	
Moderately healthy	0.47 (0.36, 0.62)	<0.001	0.54 (0.41, 0.71)	<0.001	0.6 (0.45, 0.79)	<0.001
Healthy	0.29 (0.2, 0.41)	<0.001	0.37 (0.26, 0.52)	<0.001	0.44 (0.31, 0.64)	<0.001
Per 1-point increase (continuous)	0.74 (0.69, 0.8)	<0.001	0.78 (0.72, 0.84)	<0.001	0.82 (0.75, 0.88)	<0.001
**Metabolomic signature**						
Low	Ref.		Ref.		Ref.	
Intermediate	0.55 (0.45, 0.69)	<0.001	0.6 (0.48, 0.75)	<0.001	0.65 (0.52, 0.82)	<0.001
High	0.4 (0.29, 0.55)	<0.001	0.42 (0.29, 0.6)	<0.001	0.49 (0.34, 0.7)	<0.001
Per 1-SD increase (continuous)	0.51 (0.41, 0.63)	<0.001	0.53 (0.43, 0.67)	<0.001	0.64 (0.51, 0.81)	<0.001

* Adjusted for age, sex, ethnicity, Townsend deprivation index, education level, body mass index (BMI), and a range of medication use, including antidiabetic, antihypertensive, and lipid-lowering drugs.

† Further adjusted for both the healthy lifestyle score and the metabolomic signature simultaneously in the multivariable model to assess their independent associations.

SHR, subdistribution hazard ratio; CI, confidence interval.

Supplementary Table 19. Associations of the healthy lifestyle score and the metabolomic signature with the risk of incident schizophrenia using alternative outcome definitions.

Outcome definition	Variable	Age- and sex-adjusted model		Multivariable-adjusted model *		Multivariable-adjusted + mutual adjustment †	
		HR (95% CI)	*P* value	HR (95% CI)	*P* value	HR (95% CI)	*P* value
**ICD-10 F20**							
	**Healthy Lifestyle Score**						
	Unhealthy	Ref.		Ref.		Ref.	
	Moderately healthy	0.27 (0.18, 0.42)	<0.001	0.35 (0.23, 0.54)	<0.001	0.4 (0.26, 0.61)	<0.001
	Healthy	0.18 (0.1, 0.31)	<0.001	0.27 (0.15, 0.49)	<0.001	0.34 (0.19, 0.62)	<0.001
	Per 1-point increase (continuous)	0.66 (0.58, 0.74)	<0.001	0.72 (0.64, 0.81)	<0.001	0.77 (0.68, 0.87)	<0.001
	**Metabolomic signature**						
	Low	Ref.		Ref.		Ref.	
	Intermediate	0.41 (0.28, 0.6)	<0.001	0.48 (0.33, 0.72)	<0.001	0.56 (0.37, 0.83)	0.004
	High	0.33 (0.19, 0.58)	<0.001	0.38 (0.21, 0.7)	0.002	0.47 (0.25, 0.88)	0.018
	Per 1-SD increase (continuous)	0.35 (0.25, 0.49)	<0.001	0.4 (0.27, 0.57)	<0.001	0.5 (0.34, 0.74)	<0.001
**ICD-10 F20 & F25**							
	**Healthy Lifestyle Score**						
	Unhealthy	Ref.		Ref.		Ref.	
	Moderately healthy	0.3 (0.2, 0.45)	<0.001	0.38 (0.25, 0.57)	<0.001	0.43 (0.28, 0.65)	<0.001
	Healthy	0.18 (0.11, 0.31)	<0.001	0.27 (0.16, 0.48)	<0.001	0.33 (0.19, 0.6)	<0.001
	Per 1-point increase (continuous)	0.66 (0.59, 0.74)	<0.001	0.72 (0.64, 0.8)	<0.001	0.76 (0.68, 0.86)	<0.001
	**Metabolomic signature**						
	Low	Ref.		Ref.		Ref.	
	Intermediate	0.44 (0.31, 0.62)	<0.001	0.51 (0.35, 0.74)	<0.001	0.58 (0.4, 0.85)	0.005
	High	0.34 (0.2, 0.57)	<0.001	0.38 (0.21, 0.68)	0.001	0.47 (0.26, 0.85)	0.012
	Per 1-SD increase (continuous)	0.37 (0.27, 0.51)	<0.001	0.41 (0.29, 0.58)	<0.001	0.52 (0.36, 0.75)	<0.001
**ICD-10 F20–F29 excluding F23**							
	**Healthy Lifestyle Score**						
	Unhealthy	Ref.		Ref.		Ref.	
	Moderately healthy	0.42 (0.32, 0.56)	<0.001	0.49 (0.37, 0.66)	<0.001	0.54 (0.41, 0.73)	<0.001
	Healthy	0.24 (0.17, 0.35)	<0.001	0.31 (0.22, 0.46)	<0.001	0.38 (0.26, 0.55)	<0.001
	Per 1-point increase (continuous)	0.72 (0.67, 0.77)	<0.001	0.76 (0.7, 0.81)	<0.001	0.79 (0.73, 0.85)	<0.001
	**Metabolomic signature**						
	Low	Ref.		Ref.		Ref.	
	Intermediate	0.54 (0.43, 0.67)	<0.001	0.59 (0.47, 0.75)	<0.001	0.66 (0.52, 0.84)	<0.001
	High	0.37 (0.26, 0.52)	<0.001	0.4 (0.28, 0.58)	<0.001	0.48 (0.33, 0.71)	<0.001
	Per 1-SD increase (continuous)	0.47 (0.38, 0.58)	<0.001	0.51 (0.4, 0.64)	<0.001	0.63 (0.49, 0.8)	<0.001

* Adjusted for age, sex, ethnicity, Townsend deprivation index, education level, body mass index (BMI), and a range of medication use, including antidiabetic, antihypertensive, and lipid-lowering drugs.

† Further adjusted for both the healthy lifestyle score and the metabolomic signature simultaneously in the multivariable model to assess their independent associations.

HR, hazard ratio; CI, confidence interval.

Supplementary Table 20. Associations between metabolomic signature quintiles (Q1–Q5) and risk of incident schizophrenia.

Metabolomic signature	Age- and sex-adjusted model		Multivariable-adjusted model *		Multivariable-adjusted + mutual adjustment †	
	HR (95% CI)	*P* value	HR (95% CI)	*P* value	HR (95% CI)	*P* value
Q1	Ref.		Ref.		Ref.	
Q2	0.53 (0.4, 0.71)	<0.001	0.57 (0.43, 0.76)	<0.001	0.61 (0.45, 0.82)	<0.001
Q3	0.61 (0.46, 0.8)	<0.001	0.66 (0.5, 0.89)	0.005	0.73 (0.55, 0.98)	0.036
Q4	0.47 (0.35, 0.64)	<0.001	0.52 (0.38, 0.71)	<0.001	0.59 (0.43, 0.81)	0.001
Q5	0.38 (0.28, 0.53)	<0.001	0.4 (0.28, 0.58)	<0.001	0.48 (0.33, 0.69)	<0.001

* Adjusted for age, sex, ethnicity, Townsend deprivation index, education level, body mass index (BMI), and a range of medication use, including antidiabetic, antihypertensive, and lipid-lowering drugs.

† Further adjusted for the healthy lifestyle score.

HR, hazard ratio; CI, confidence interval.

Supplementary Table 21. Sensitivity analyses of the mediation effect of the metabolomic signature on the association between healthy lifestyle score and incident schizophrenia.

**Sensitivity analysis**	**Total effect (95% CI)**	***P* value**	**Total natural direct effect (95% CI)**	***P* value**	**Total natural indirect effect (95% CI)**	***P* value**	**Proportion of effect mediated (95% CI)**
Main analysis	0.7737 (0.7185, 0.7993)	< 0.001	0.8089 (0.7576, 0.8381)	< 0.001	0.9564 (0.9379, 0.9667)	< 0.001	0.1559 (0.1082, 0.205)
F20 only	0.7198 (0.6524, 0.7997)	< 0.001	0.7683 (0.6942, 0.853)	< 0.001	0.9369 (0.907, 0.9536)	< 0.001	0.1731 (0.1134, 0.3116)
F20 and F25 combined	0.717 (0.6489, 0.7786)	< 0.001	0.7626 (0.6942, 0.824)	< 0.001	0.9402 (0.9134, 0.9684)	< 0.001	0.1612 (0.0825, 0.2866)
F20–F29 excluding F23	0.7558 (0.7086, 0.7802)	< 0.001	0.7898 (0.742, 0.8208)	< 0.001	0.957 (0.9449, 0.9688)	< 0.001	0.1392 (0.0938, 0.1989)
Excluding first 2 years	0.7556 (0.6947, 0.7804)	< 0.001	0.7922 (0.7293, 0.819)	< 0.001	0.9538 (0.9446, 0.9691)	< 0.001	0.1499 (0.0846, 0.1776)
Excluding first 5 years	0.7609 (0.724, 0.8199)	< 0.001	0.7941 (0.7503, 0.8557)	< 0.001	0.9582 (0.9329, 0.9938)	< 0.001	0.1388 (0.0216, 0.2358)
Excluding baseline dementia or organic psychosis	0.7717 (0.6994, 0.8009)	< 0.001	0.8068 (0.733, 0.8336)	< 0.001	0.9565 (0.9512, 0.9745)	< 0.001	0.1537 (0.0934, 0.1801)
Additional adjustment (cancer/CVD)	0.7773 (0.7685, 0.8475)	< 0.001	0.8126 (0.8084, 0.8774)	< 0.001	0.9565 (0.9383, 0.9719)	< 0.001	0.1587 (0.1288, 0.2678)
Imputed lifestyle data	0.7643 (0.7374, 0.7912)	< 0.001	0.8063 (0.7779, 0.8399)	< 0.001	0.9478 (0.9369, 0.9685)	< 0.001	0.1785 (0.1067, 0.2365)

HR, hazard ratio; CI, confidence interval; CVD, cardiovascular disease.
