# Supplementary material for: Healthy lifestyle, metabolomic signature, and risk of late-onset schizophrenia: evidence from the prospective cohort
Source: Schizophrenia (Heidelb). 2026 Apr 14;12(1):50. doi: 10.1038/s41537-026-00752-z (PMC13270109; doi:10.1038/s41537-026-00752-z)
Supplement: Supplementary file 2 — Supplementary Material 2 (PDF) [file 41537_2026_752_MOESM2_ESM.pdf]

Supplementary Table 3. Information of 251 circulating metabolites in the UK Biobank.

Field ID	Metabolic biomarkers	Description	Group	Subgroup	Unit	Numerator Field ID	Denominator Field ID
23610	IDL_C_pct	Cholesterol to total lipids ratio in IDL	Relative lipoprotein lipid concentrations	IDL ratios	%	23526	23524
23580	XXL_VLDL_C_pct	Cholesterol to total lipids ratio in chylomicrons and extremely large VLDL	Relative lipoprotein lipid concentrations	Chylomicrons and extremely large VLDL ratios	%	23484	23482
23635	L_HDL_C_pct	Cholesterol to total lipids ratio in large HDL	Relative lipoprotein lipid concentrations	Large HDL ratios	%	23561	23559
23615	L_LDL_C_pct	Cholesterol to total lipids ratio in large LDL	Relative lipoprotein lipid concentrations	Large LDL ratios	%	23533	23531
23590	L_VLDL_C_pct	Cholesterol to total lipids ratio in large VLDL	Relative lipoprotein lipid concentrations	Large VLDL ratios	%	23498	23496
23640	M_HDL_C_pct	Cholesterol to total lipids ratio in medium HDL	Relative lipoprotein lipid concentrations	Medium HDL ratios	%	23568	23566
23620	M_LDL_C_pct	Cholesterol to total lipids ratio in medium LDL	Relative lipoprotein lipid concentrations	Medium LDL ratios	%	23540	23538
23595	M_VLDL_C_pct	Cholesterol to total lipids ratio in medium VLDL	Relative lipoprotein lipid concentrations	Medium VLDL ratios	%	23505	23503
23645	S_HDL_C_pct	Cholesterol to total lipids ratio in small HDL	Relative lipoprotein lipid concentrations	Small HDL ratios	%	23575	23573
23625	S_LDL_C_pct	Cholesterol to total lipids ratio in small LDL	Relative lipoprotein lipid concentrations	Small LDL ratios	%	23547	23545
23600	S_VLDL_C_pct	Cholesterol to total lipids ratio in small VLDL	Relative lipoprotein lipid concentrations	Small VLDL ratios	%	23512	23510
23630	XL_HDL_C_pct	Cholesterol to total lipids ratio in very large HDL	Relative lipoprotein lipid concentrations	Very large HDL ratios	%	23554	23552
23585	XL_VLDL_C_pct	Cholesterol to total lipids ratio in very large VLDL	Relative lipoprotein lipid concentrations	Very large VLDL ratios	%	23491	23489
23605	XS_VLDL_C_pct	Cholesterol to total lipids ratio in very small VLDL	Relative lipoprotein lipid concentrations	Very small VLDL ratios	%	23519	23517
23611	IDL_CE_pct	Cholesteryl esters to total lipids ratio in IDL	Relative lipoprotein lipid concentrations	IDL ratios	%	23527	23524
23581	XXL_VLDL_CE_pct	Cholesteryl esters to total lipids ratio in chylomicrons and extremely large VLDL	Relative lipoprotein lipid concentrations	Chylomicrons and extremely large VLDL ratios	%	23485	23482
23636	L_HDL_CE_pct	Cholesteryl esters to total lipids ratio in large HDL	Relative lipoprotein lipid concentrations	Large HDL ratios	%	23562	23559
23616	L_LDL_CE_pct	Cholesteryl esters to total lipids ratio in large LDL	Relative lipoprotein lipid concentrations	Large LDL ratios	%	23534	23531
23591	L_VLDL_CE_pct	Cholesteryl esters to total lipids ratio in large VLDL	Relative lipoprotein lipid concentrations	Large VLDL ratios	%	23499	23496
23641	M_HDL_CE_pct	Cholesteryl esters to total lipids ratio in medium HDL	Relative lipoprotein lipid concentrations	Medium HDL ratios	%	23569	23566
23621	M_LDL_CE_pct	Cholesteryl esters to total lipids ratio in medium LDL	Relative lipoprotein lipid concentrations	Medium LDL ratios	%	23541	23538
23596	M_VLDL_CE_pct	Cholesteryl esters to total lipids ratio in medium VLDL	Relative lipoprotein lipid concentrations	Medium VLDL ratios	%	23506	23503
23646	S_HDL_CE_pct	Cholesteryl esters to total lipids ratio in small HDL	Relative lipoprotein lipid concentrations	Small HDL ratios	%	23576	23573
23626	S_LDL_CE_pct	Cholesteryl esters to total lipids ratio in small LDL	Relative lipoprotein lipid concentrations	Small LDL ratios	%	23548	23545
23601	S_VLDL_CE_pct	Cholesteryl esters to total lipids ratio in small VLDL	Relative lipoprotein lipid concentrations	Small VLDL ratios	%	23513	23510
23631	XL_HDL_CE_pct	Cholesteryl esters to total lipids ratio in very large HDL	Relative lipoprotein lipid concentrations	Very large HDL ratios	%	23555	23552
23586	XL_VLDL_CE_pct	Cholesteryl esters to total lipids ratio in very large VLDL	Relative lipoprotein lipid concentrations	Very large VLDL ratios	%	23492	23489
23606	XS_VLDL_CE_pct	Cholesteryl esters to total lipids ratio in very small VLDL	Relative lipoprotein lipid concentrations	Very small VLDL ratios	%	23520	23517
23612	IDL_FC_pct	Free cholesterol to total lipids ratio in IDL	Relative lipoprotein lipid concentrations	IDL ratios	%	23528	23524
23582	XXL_VLDL_FC_pct	Free cholesterol to total lipids ratio in chylomicrons and extremely large VLDL	Relative lipoprotein lipid concentrations	Chylomicrons and extremely large VLDL ratios	%	23486	23482
23637	L_HDL_FC_pct	Free cholesterol to total lipids ratio in large HDL	Relative lipoprotein lipid concentrations	Large HDL ratios	%	23563	23559
23617	L_LDL_FC_pct	Free cholesterol to total lipids ratio in large LDL	Relative lipoprotein lipid concentrations	Large LDL ratios	%	23535	23531
23592	L_VLDL_FC_pct	Free cholesterol to total lipids ratio in large VLDL	Relative lipoprotein lipid concentrations	Large VLDL ratios	%	23500	23496
23642	M_HDL_FC_pct	Free cholesterol to total lipids ratio in medium HDL	Relative lipoprotein lipid concentrations	Medium HDL ratios	%	23570	23566
23622	M_LDL_FC_pct	Free cholesterol to total lipids ratio in medium LDL	Relative lipoprotein lipid concentrations	Medium LDL ratios	%	23542	23538
23597	M_VLDL_FC_pct	Free cholesterol to total lipids ratio in medium VLDL	Relative lipoprotein lipid concentrations	Medium VLDL ratios	%	23507	23503
23647	S_HDL_FC_pct	Free cholesterol to total lipids ratio in small HDL	Relative lipoprotein lipid concentrations	Small HDL ratios	%	23577	23573
23627	S_LDL_FC_pct	Free cholesterol to total lipids ratio in small LDL	Relative lipoprotein lipid concentrations	Small LDL ratios	%	23549	23545
23602	S_VLDL_FC_pct	Free cholesterol to total lipids ratio in small VLDL	Relative lipoprotein lipid concentrations	Small VLDL ratios	%	23514	23510
23632	XL_HDL_FC_pct	Free cholesterol to total lipids ratio in very large HDL	Relative lipoprotein lipid concentrations	Very large HDL ratios	%	23556	23552
23587	XL_VLDL_FC_pct	Free cholesterol to total lipids ratio in very large VLDL	Relative lipoprotein lipid concentrations	Very large VLDL ratios	%	23493	23489
23607	XS_VLDL_FC_pct	Free cholesterol to total lipids ratio in very small VLDL	Relative lipoprotein lipid concentrations	Very small VLDL ratios	%	23521	23517
23609	IDL_PL_pct	Phospholipids to total lipids ratio in IDL	Relative lipoprotein lipid concentrations	IDL ratios	%	23525	23524
23579	XXL_VLDL_PL_pct	Phospholipids to total lipids ratio in chylomicrons and extremely large VLDL	Relative lipoprotein lipid concentrations	Chylomicrons and extremely large VLDL ratios	%	23483	23482
23634	L_HDL_PL_pct	Phospholipids to total lipids ratio in large HDL	Relative lipoprotein lipid concentrations	Large HDL ratios	%	23560	23559
23614	L_LDL_PL_pct	Phospholipids to total lipids ratio in large LDL	Relative lipoprotein lipid concentrations	Large LDL ratios	%	23532	23531
23589	L_VLDL_PL_pct	Phospholipids to total lipids ratio in large VLDL	Relative lipoprotein lipid concentrations	Large VLDL ratios	%	23497	23496
23639	M_HDL_PL_pct	Phospholipids to total lipids ratio in medium HDL	Relative lipoprotein lipid concentrations	Medium HDL ratios	%	23567	23566
23619	M_LDL_PL_pct	Phospholipids to total lipids ratio in medium LDL	Relative lipoprotein lipid concentrations	Medium LDL ratios	%	23539	23538
23594	M_VLDL_PL_pct	Phospholipids to total lipids ratio in medium VLDL	Relative lipoprotein lipid concentrations	Medium VLDL ratios	%	23504	23503
23644	S_HDL_PL_pct	Phospholipids to total lipids ratio in small HDL	Relative lipoprotein lipid concentrations	Small HDL ratios	%	23574	23573
23624	S_LDL_PL_pct	Phospholipids to total lipids ratio in small LDL	Relative lipoprotein lipid concentrations	Small LDL ratios	%	23546	23545
23599	S_VLDL_PL_pct	Phospholipids to total lipids ratio in small VLDL	Relative lipoprotein lipid concentrations	Small VLDL ratios	%	23511	23510
23629	XL_HDL_PL_pct	Phospholipids to total lipids ratio in very large HDL	Relative lipoprotein lipid concentrations	Very large HDL ratios	%	23553	23552
23584	XL_VLDL_PL_pct	Phospholipids to total lipids ratio in very large VLDL	Relative lipoprotein lipid concentrations	Very large VLDL ratios	%	23490	23489
23604	XS_VLDL_PL_pct	Phospholipids to total lipids ratio in very small VLDL	Relative lipoprotein lipid concentrations	Very small VLDL ratios	%	23518	23517
23441	ApoB_by_ApoA1	Ratio of apolipoprotein B to apolipoprotein A1	Apolipoproteins		ratio	23439	23440
23457	DHA_pct	Ratio of docosahexaenoic acid to total fatty acids	Fatty acids	Fatty acid ratios	%	23450	23442
23456	LA_pct	Ratio of linoleic acid to total fatty acids	Fatty acids	Fatty acid ratios	%	23449	23442
23454	MUFA_pct	Ratio of monounsaturated fatty acids to total fatty acids	Fatty acids	Fatty acid ratios	%	23447	23442
23451	Omega_3_pct	Ratio of omega-3 fatty acids to total fatty acids	Fatty acids	Fatty acid ratios	%	23444	23442
23459	Omega_6_by_Omega_3	Ratio of omega-6 fatty acids to omega-3 fatty acids	Fatty acids	Fatty acid ratios	ratio	23445	23444
23452	Omega_6_pct	Ratio of omega-6 fatty acids to total fatty acids	Fatty acids	Fatty acid ratios	%	23445	23442
23458	PUFA_by_MUFA	Ratio of polyunsaturated fatty acids to monounsaturated fatty acids	Fatty acids	Fatty acid ratios	ratio	23446	23447
23453	PUFA_pct	Ratio of polyunsaturated fatty acids to total fatty acids	Fatty acids	Fatty acid ratios	%	23446	23442
23455	SFA_pct	Ratio of saturated fatty acids to total fatty acids	Fatty acids	Fatty acid ratios	%	23448	23442
23435	TG_by_PG	Ratio of triglycerides to phosphoglycerides	Other lipids		ratio	23407	23434
23613	IDL_TG_pct	Triglycerides to total lipids ratio in IDL	Relative lipoprotein lipid concentrations	IDL ratios	%	23529	23524
23583	XXL_VLDL_TG_pct	Triglycerides to total lipids ratio in chylomicrons and extremely large VLDL	Relative lipoprotein lipid concentrations	Chylomicrons and extremely large VLDL ratios	%	23487	23482
23638	L_HDL_TG_pct	Triglycerides to total lipids ratio in large HDL	Relative lipoprotein lipid concentrations	Large HDL ratios	%	23564	23559
23618	L_LDL_TG_pct	Triglycerides to total lipids ratio in large LDL	Relative lipoprotein lipid concentrations	Large LDL ratios	%	23536	23531
23593	L_VLDL_TG_pct	Triglycerides to total lipids ratio in large VLDL	Relative lipoprotein lipid concentrations	Large VLDL ratios	%	23501	23496
23643	M_HDL_TG_pct	Triglycerides to total lipids ratio in medium HDL	Relative lipoprotein lipid concentrations	Medium HDL ratios	%	23571	23566
23623	M_LDL_TG_pct	Triglycerides to total lipids ratio in medium LDL	Relative lipoprotein lipid concentrations	Medium LDL ratios	%	23543	23538
23598	M_VLDL_TG_pct	Triglycerides to total lipids ratio in medium VLDL	Relative lipoprotein lipid concentrations	Medium VLDL ratios	%	23508	23503
23648	S_HDL_TG_pct	Triglycerides to total lipids ratio in small HDL	Relative lipoprotein lipid concentrations	Small HDL ratios	%	23578	23573
23628	S_LDL_TG_pct	Triglycerides to total lipids ratio in small LDL	Relative lipoprotein lipid concentrations	Small LDL ratios	%	23550	23545
23603	S_VLDL_TG_pct	Triglycerides to total lipids ratio in small VLDL	Relative lipoprotein lipid concentrations	Small VLDL ratios	%	23515	23510
23633	XL_HDL_TG_pct	Triglycerides to total lipids ratio in very large HDL	Relative lipoprotein lipid concentrations	Very large HDL ratios	%	23557	23552
23588	XL_VLDL_TG_pct	Triglycerides to total lipids ratio in very large VLDL	Relative lipoprotein lipid concentrations	Very large VLDL ratios	%	23494	23489
23608	XS_VLDL_TG_pct	Triglycerides to total lipids ratio in very small VLDL	Relative lipoprotein lipid concentrations	Very small VLDL ratios	%	23522	23517
20280	GL	Glucose-lactate	Glycolysis related metabolites		mmol/L	NA	NA
20281	SCA	Spectrometer-corrected alanine	Amino acids		mmol/L	NA	NA
23400	Total_C	Total cholesterol	Cholesterol		mmol/L	NA	NA
23401	non_HDL_C	Total cholesterol minus HDL-C	Cholesterol		mmol/L	NA	NA
23402	Remnant_C	Remnant cholesterol (non-HDL, non-LDL -cholesterol)	Cholesterol		mmol/L	NA	NA
23403	VLDL_C	VLDL cholesterol	Cholesterol		mmol/L	NA	NA
23404	Clinical_LDL_C	Clinical LDL cholesterol	Cholesterol		mmol/L	NA	NA
23405	LDL_C	LDL cholesterol	Cholesterol		mmol/L	NA	NA
23406	HDL_C	HDL cholesterol	Cholesterol		mmol/L	NA	NA
23407	Total_TG	Total triglycerides	Triglycerides		mmol/L	NA	NA
23408	VLDL_TG	Triglycerides in VLDL	Triglycerides		mmol/L	NA	NA
23409	LDL_TG	Triglycerides in LDL	Triglycerides		mmol/L	NA	NA
23410	HDL_TG	Triglycerides in HDL	Triglycerides		mmol/L	NA	NA
23411	Total_PL	Total phospholipids in lipoprotein particles	Phospholipids		mmol/L	NA	NA
23412	VLDL_PL	Phospholipids in VLDL	Phospholipids		mmol/L	NA	NA
23413	LDL_PL	Phospholipids in LDL	Phospholipids		mmol/L	NA	NA
23414	HDL_PL	Phospholipids in HDL	Phospholipids		mmol/L	NA	NA
23415	Total_CE	Total esterified cholesterol	Cholesteryl esters		mmol/L	NA	NA
23416	VLDL_CE	Cholesteryl esters in VLDL	Cholesteryl esters		mmol/L	NA	NA
23417	LDL_CE	Cholesteryl esters in LDL	Cholesteryl esters		mmol/L	NA	NA
23418	HDL_CE	Cholesteryl esters in HDL	Cholesteryl esters		mmol/L	NA	NA
23419	Total_FC	Total free cholesterol	Free cholesterol		mmol/L	NA	NA
23420	VLDL_FC	Free cholesterol in VLDL	Free cholesterol		mmol/L	NA	NA
23421	LDL_FC	Free cholesterol in LDL	Free cholesterol		mmol/L	NA	NA
23422	HDL_FC	Free cholesterol in HDL	Free cholesterol		mmol/L	NA	NA
23423	Total_L	Total lipids in lipoprotein particles	Total lipids		mmol/L	NA	NA
23424	VLDL_L	Total lipids in VLDL	Total lipids		mmol/L	NA	NA
23425	LDL_L	Total lipids in LDL	Total lipids		mmol/L	NA	NA
23426	HDL_L	Total lipids in HDL	Total lipids		mmol/L	NA	NA
23427	Total_P	Total concentration of lipoprotein particles	Lipoprotein particle concentrations		mmol/L	NA	NA
23428	VLDL_P	Concentration of VLDL particles	Lipoprotein particle concentrations		mmol/L	NA	NA
23429	LDL_P	Concentration of LDL particles	Lipoprotein particle concentrations		mmol/L	NA	NA
23430	HDL_P	Concentration of HDL particles	Lipoprotein particle concentrations		mmol/L	NA	NA
23431	VLDL_size	Average diameter for VLDL particles	Lipoprotein particle sizes		nm	NA	NA
23432	LDL_size	Average diameter for LDL particles	Lipoprotein particle sizes		nm	NA	NA
23433	HDL_size	Average diameter for HDL particles	Lipoprotein particle sizes		nm	NA	NA
23434	Phosphoglyc	Phosphoglycerides	Other lipids		mmol/L	NA	NA
23436	Cholines	Total cholines	Other lipids		mmol/L	NA	NA
23437	Phosphatidylc	Phosphatidylcholines	Other lipids		mmol/L	NA	NA
23438	Sphingomyelins	Sphingomyelins	Other lipids		mmol/L	NA	NA
23439	ApoB	Apolipoprotein B	Apolipoproteins		g/l	NA	NA
23440	ApoA1	Apolipoprotein A1	Apolipoproteins		g/l	NA	NA
23442	Total_FA	Total fatty acids	Fatty acids		mmol/L	NA	NA
23443	Unsaturation	Degree of unsaturation	Fatty acids		degree	NA	NA
23444	Omega_3	Omega-3 fatty acids	Fatty acids		mmol/L	NA	NA
23445	Omega_6	Omega-6 fatty acids	Fatty acids		mmol/L	NA	NA
23446	PUFA	Polyunsaturated fatty acids	Fatty acids		mmol/L	NA	NA
23447	MUFA	Monounsaturated fatty acids	Fatty acids		mmol/L	NA	NA
23448	SFA	Saturated fatty acids	Fatty acids		mmol/L	NA	NA
23449	LA	Linoleic acid	Fatty acids		mmol/L	NA	NA
23450	DHA	Docosahexaenoic acid	Fatty acids		mmol/L	NA	NA
23460	Ala	Alanine	Amino acids		mmol/L	NA	NA
23461	Gln	Glutamine	Amino acids		mmol/L	NA	NA
23462	Gly	Glycine	Amino acids		mmol/L	NA	NA
23463	His	Histidine	Amino acids		mmol/L	NA	NA
23464	Total_BCAA	Total concentration of branched-chain amino acids (leucine + isoleucine + valine)	Amino acids	Branched-chain amino acids	mmol/L	NA	NA
23465	Ile	Isoleucine	Amino acids	Branched-chain amino acids	mmol/L	NA	NA
23466	Leu	Leucine	Amino acids	Branched-chain amino acids	mmol/L	NA	NA
23467	Val	Valine	Amino acids	Branched-chain amino acids	mmol/L	NA	NA
23468	Phe	Phenylalanine	Amino acids	Aromatic amino acids	mmol/L	NA	NA
23469	Tyr	Tyrosine	Amino acids	Aromatic amino acids	mmol/L	NA	NA
23470	Glucose	Glucose	Glycolysis related metabolites		mmol/L	NA	NA
23471	Lactate	Lactate	Glycolysis related metabolites		mmol/L	NA	NA
23472	Pyruvate	Pyruvate	Glycolysis related metabolites		mmol/L	NA	NA
23473	Citrate	Citrate	Glycolysis related metabolites		mmol/L	NA	NA
23474	bOHbutyrate	3-Hydroxybutyrate	Ketone bodies		mmol/L	NA	NA
23475	Acetate	Acetate	Ketone bodies		mmol/L	NA	NA
23476	Acetoacetate	Acetoacetate	Ketone bodies		mmol/L	NA	NA
23477	Acetone	Acetone	Ketone bodies		mmol/L	NA	NA
23478	Creatinine	Creatinine	Fluid balance		mmol/L	NA	NA
23479	Albumin	Albumin	Fluid balance		g/l	NA	NA
23480	GlycA	Glycoprotein acetyls	Inflammation		mmol/L	NA	NA
23481	XXL_VLDL_P	Concentration of chylomicrons and extremely large VLDL particles	Lipoprotein subclasses	Chylomicrons and extremely large VLDL (particle diameters from 75 nm upwards)	mmol/L	NA	NA
23482	XXL_VLDL_L	Total lipids in chylomicrons and extremely large VLDL	Lipoprotein subclasses	Chylomicrons and extremely large VLDL (particle diameters from 75 nm upwards)	mmol/L	NA	NA
23483	XXL_VLDL_PL	Phospholipids in chylomicrons and extremely large VLDL	Lipoprotein subclasses	Chylomicrons and extremely large VLDL (particle diameters from 75 nm upwards)	mmol/L	NA	NA
23484	XXL_VLDL_C	Cholesterol in chylomicrons and extremely large VLDL	Lipoprotein subclasses	Chylomicrons and extremely large VLDL (particle diameters from 75 nm upwards)	mmol/L	NA	NA
23485	XXL_VLDL_CE	Cholesteryl esters in chylomicrons and extremely large VLDL	Lipoprotein subclasses	Chylomicrons and extremely large VLDL (particle diameters from 75 nm upwards)	mmol/L	NA	NA
23486	XXL_VLDL_FC	Free cholesterol in chylomicrons and extremely large VLDL	Lipoprotein subclasses	Chylomicrons and extremely large VLDL (particle diameters from 75 nm upwards)	mmol/L	NA	NA
23487	XXL_VLDL_TG	Triglycerides in chylomicrons and extremely large VLDL	Lipoprotein subclasses	Chylomicrons and extremely large VLDL (particle diameters from 75 nm upwards)	mmol/L	NA	NA
23488	XL_VLDL_P	Concentration of very large VLDL particles	Lipoprotein subclasses	Very large VLDL (average diameter 64 nm)	mmol/L	NA	NA
23489	XL_VLDL_L	Total lipids in very large VLDL	Lipoprotein subclasses	Very large VLDL (average diameter 64 nm)	mmol/L	NA	NA
23490	XL_VLDL_PL	Phospholipids in very large VLDL	Lipoprotein subclasses	Very large VLDL (average diameter 64 nm)	mmol/L	NA	NA
23491	XL_VLDL_C	Cholesterol in very large VLDL	Lipoprotein subclasses	Very large VLDL (average diameter 64 nm)	mmol/L	NA	NA
23492	XL_VLDL_CE	Cholesteryl esters in very large VLDL	Lipoprotein subclasses	Very large VLDL (average diameter 64 nm)	mmol/L	NA	NA
23493	XL_VLDL_FC	Free cholesterol in very large VLDL	Lipoprotein subclasses	Very large VLDL (average diameter 64 nm)	mmol/L	NA	NA
23494	XL_VLDL_TG	Triglycerides in very large VLDL	Lipoprotein subclasses	Very large VLDL (average diameter 64 nm)	mmol/L	NA	NA
23495	L_VLDL_P	Concentration of large VLDL particles	Lipoprotein subclasses	Large VLDL (average diameter 53.6 nm)	mmol/L	NA	NA
23496	L_VLDL_L	Total lipids in large VLDL	Lipoprotein subclasses	Large VLDL (average diameter 53.6 nm)	mmol/L	NA	NA
23497	L_VLDL_PL	Phospholipids in large VLDL	Lipoprotein subclasses	Large VLDL (average diameter 53.6 nm)	mmol/L	NA	NA
23498	L_VLDL_C	Cholesterol in large VLDL	Lipoprotein subclasses	Large VLDL (average diameter 53.6 nm)	mmol/L	NA	NA
23499	L_VLDL_CE	Cholesteryl esters in large VLDL	Lipoprotein subclasses	Large VLDL (average diameter 53.6 nm)	mmol/L	NA	NA
23500	L_VLDL_FC	Free cholesterol in large VLDL	Lipoprotein subclasses	Large VLDL (average diameter 53.6 nm)	mmol/L	NA	NA
23501	L_VLDL_TG	Triglycerides in large VLDL	Lipoprotein subclasses	Large VLDL (average diameter 53.6 nm)	mmol/L	NA	NA
23502	M_VLDL_P	Concentration of medium VLDL particles	Lipoprotein subclasses	Medium VLDL (average diameter 44.5 nm)	mmol/L	NA	NA
23503	M_VLDL_L	Total lipids in medium VLDL	Lipoprotein subclasses	Medium VLDL (average diameter 44.5 nm)	mmol/L	NA	NA
23504	M_VLDL_PL	Phospholipids in medium VLDL	Lipoprotein subclasses	Medium VLDL (average diameter 44.5 nm)	mmol/L	NA	NA
23505	M_VLDL_C	Cholesterol in medium VLDL	Lipoprotein subclasses	Medium VLDL (average diameter 44.5 nm)	mmol/L	NA	NA
23506	M_VLDL_CE	Cholesteryl esters in medium VLDL	Lipoprotein subclasses	Medium VLDL (average diameter 44.5 nm)	mmol/L	NA	NA
23507	M_VLDL_FC	Free cholesterol in medium VLDL	Lipoprotein subclasses	Medium VLDL (average diameter 44.5 nm)	mmol/L	NA	NA
23508	M_VLDL_TG	Triglycerides in medium VLDL	Lipoprotein subclasses	Medium VLDL (average diameter 44.5 nm)	mmol/L	NA	NA
23509	S_VLDL_P	Concentration of small VLDL particles	Lipoprotein subclasses	Small VLDL (average diameter 36.8 nm)	mmol/L	NA	NA
23510	S_VLDL_L	Total lipids in small VLDL	Lipoprotein subclasses	Small VLDL (average diameter 36.8 nm)	mmol/L	NA	NA
23511	S_VLDL_PL	Phospholipids in small VLDL	Lipoprotein subclasses	Small VLDL (average diameter 36.8 nm)	mmol/L	NA	NA
23512	S_VLDL_C	Cholesterol in small VLDL	Lipoprotein subclasses	Small VLDL (average diameter 36.8 nm)	mmol/L	NA	NA
23513	S_VLDL_CE	Cholesteryl esters in small VLDL	Lipoprotein subclasses	Small VLDL (average diameter 36.8 nm)	mmol/L	NA	NA
23514	S_VLDL_FC	Free cholesterol in small VLDL	Lipoprotein subclasses	Small VLDL (average diameter 36.8 nm)	mmol/L	NA	NA
23515	S_VLDL_TG	Triglycerides in small VLDL	Lipoprotein subclasses	Small VLDL (average diameter 36.8 nm)	mmol/L	NA	NA
23516	XS_VLDL_P	Concentration of very small VLDL particles	Lipoprotein subclasses	Very small VLDL (average diameter 31.3 nm)	mmol/L	NA	NA
23517	XS_VLDL_L	Total lipids in very small VLDL	Lipoprotein subclasses	Very small VLDL (average diameter 31.3 nm)	mmol/L	NA	NA
23518	XS_VLDL_PL	Phospholipids in very small VLDL	Lipoprotein subclasses	Very small VLDL (average diameter 31.3 nm)	mmol/L	NA	NA
23519	XS_VLDL_C	Cholesterol in very small VLDL	Lipoprotein subclasses	Very small VLDL (average diameter 31.3 nm)	mmol/L	NA	NA
23520	XS_VLDL_CE	Cholesteryl esters in very small VLDL	Lipoprotein subclasses	Very small VLDL (average diameter 31.3 nm)	mmol/L	NA	NA
23521	XS_VLDL_FC	Free cholesterol in very small VLDL	Lipoprotein subclasses	Very small VLDL (average diameter 31.3 nm)	mmol/L	NA	NA
23522	XS_VLDL_TG	Triglycerides in very small VLDL	Lipoprotein subclasses	Very small VLDL (average diameter 31.3 nm)	mmol/L	NA	NA
23523	IDL_P	Concentration of IDL particles	Lipoprotein subclasses	IDL (average diameter 28.6 nm)	mmol/L	NA	NA
23524	IDL_L	Total lipids in IDL	Lipoprotein subclasses	IDL (average diameter 28.6 nm)	mmol/L	NA	NA
23525	IDL_PL	Phospholipids in IDL	Lipoprotein subclasses	IDL (average diameter 28.6 nm)	mmol/L	NA	NA
23526	IDL_C	Cholesterol in IDL	Lipoprotein subclasses	IDL (average diameter 28.6 nm)	mmol/L	NA	NA
23527	IDL_CE	Cholesteryl esters in IDL	Lipoprotein subclasses	IDL (average diameter 28.6 nm)	mmol/L	NA	NA
23528	IDL_FC	Free cholesterol in IDL	Lipoprotein subclasses	IDL (average diameter 28.6 nm)	mmol/L	NA	NA
23529	IDL_TG	Triglycerides in IDL	Lipoprotein subclasses	IDL (average diameter 28.6 nm)	mmol/L	NA	NA
23530	L_LDL_P	Concentration of large LDL particles	Lipoprotein subclasses	Large LDL (average diameter 25.5 nm)	mmol/L	NA	NA
23531	L_LDL_L	Total lipids in large LDL	Lipoprotein subclasses	Large LDL (average diameter 25.5 nm)	mmol/L	NA	NA
23532	L_LDL_PL	Phospholipids in large LDL	Lipoprotein subclasses	Large LDL (average diameter 25.5 nm)	mmol/L	NA	NA
23533	L_LDL_C	Cholesterol in large LDL	Lipoprotein subclasses	Large LDL (average diameter 25.5 nm)	mmol/L	NA	NA
23534	L_LDL_CE	Cholesteryl esters in large LDL	Lipoprotein subclasses	Large LDL (average diameter 25.5 nm)	mmol/L	NA	NA
23535	L_LDL_FC	Free cholesterol in large LDL	Lipoprotein subclasses	Large LDL (average diameter 25.5 nm)	mmol/L	NA	NA
23536	L_LDL_TG	Triglycerides in large LDL	Lipoprotein subclasses	Large LDL (average diameter 25.5 nm)	mmol/L	NA	NA
23537	M_LDL_P	Concentration of medium LDL particles	Lipoprotein subclasses	Medium LDL (average diameter 23 nm)	mmol/L	NA	NA
23538	M_LDL_L	Total lipids in medium LDL	Lipoprotein subclasses	Medium LDL (average diameter 23 nm)	mmol/L	NA	NA
23539	M_LDL_PL	Phospholipids in medium LDL	Lipoprotein subclasses	Medium LDL (average diameter 23 nm)	mmol/L	NA	NA
23540	M_LDL_C	Cholesterol in medium LDL	Lipoprotein subclasses	Medium LDL (average diameter 23 nm)	mmol/L	NA	NA
23541	M_LDL_CE	Cholesteryl esters in medium LDL	Lipoprotein subclasses	Medium LDL (average diameter 23 nm)	mmol/L	NA	NA
23542	M_LDL_FC	Free cholesterol in medium LDL	Lipoprotein subclasses	Medium LDL (average diameter 23 nm)	mmol/L	NA	NA
23543	M_LDL_TG	Triglycerides in medium LDL	Lipoprotein subclasses	Medium LDL (average diameter 23 nm)	mmol/L	NA	NA
23544	S_LDL_P	Concentration of small LDL particles	Lipoprotein subclasses	Small LDL (average diameter 18.7 nm)	mmol/L	NA	NA
23545	S_LDL_L	Total lipids in small LDL	Lipoprotein subclasses	Small LDL (average diameter 18.7 nm)	mmol/L	NA	NA
23546	S_LDL_PL	Phospholipids in small LDL	Lipoprotein subclasses	Small LDL (average diameter 18.7 nm)	mmol/L	NA	NA
23547	S_LDL_C	Cholesterol in small LDL	Lipoprotein subclasses	Small LDL (average diameter 18.7 nm)	mmol/L	NA	NA
23548	S_LDL_CE	Cholesteryl esters in small LDL	Lipoprotein subclasses	Small LDL (average diameter 18.7 nm)	mmol/L	NA	NA
23549	S_LDL_FC	Free cholesterol in small LDL	Lipoprotein subclasses	Small LDL (average diameter 18.7 nm)	mmol/L	NA	NA
23550	S_LDL_TG	Triglycerides in small LDL	Lipoprotein subclasses	Small LDL (average diameter 18.7 nm)	mmol/L	NA	NA
23551	XL_HDL_P	Concentration of very large HDL particles	Lipoprotein subclasses	Very large HDL (average diameter 14.3 nm)	mmol/L	NA	NA
23552	XL_HDL_L	Total lipids in very large HDL	Lipoprotein subclasses	Very large HDL (average diameter 14.3 nm)	mmol/L	NA	NA
23553	XL_HDL_PL	Phospholipids in very large HDL	Lipoprotein subclasses	Very large HDL (average diameter 14.3 nm)	mmol/L	NA	NA
23554	XL_HDL_C	Cholesterol in very large HDL	Lipoprotein subclasses	Very large HDL (average diameter 14.3 nm)	mmol/L	NA	NA
23555	XL_HDL_CE	Cholesteryl esters in very large HDL	Lipoprotein subclasses	Very large HDL (average diameter 14.3 nm)	mmol/L	NA	NA
23556	XL_HDL_FC	Free cholesterol in very large HDL	Lipoprotein subclasses	Very large HDL (average diameter 14.3 nm)	mmol/L	NA	NA
23557	XL_HDL_TG	Triglycerides in very large HDL	Lipoprotein subclasses	Very large HDL (average diameter 14.3 nm)	mmol/L	NA	NA
23558	L_HDL_P	Concentration of large HDL particles	Lipoprotein subclasses	Large HDL (average diameter 12.1 nm)	mmol/L	NA	NA
23559	L_HDL_L	Total lipids in large HDL	Lipoprotein subclasses	Large HDL (average diameter 12.1 nm)	mmol/L	NA	NA
23560	L_HDL_PL	Phospholipids in large HDL	Lipoprotein subclasses	Large HDL (average diameter 12.1 nm)	mmol/L	NA	NA
23561	L_HDL_C	Cholesterol in large HDL	Lipoprotein subclasses	Large HDL (average diameter 12.1 nm)	mmol/L	NA	NA
23562	L_HDL_CE	Cholesteryl esters in large HDL	Lipoprotein subclasses	Large HDL (average diameter 12.1 nm)	mmol/L	NA	NA
23563	L_HDL_FC	Free cholesterol in large HDL	Lipoprotein subclasses	Large HDL (average diameter 12.1 nm)	mmol/L	NA	NA
23564	L_HDL_TG	Triglycerides in large HDL	Lipoprotein subclasses	Large HDL (average diameter 12.1 nm)	mmol/L	NA	NA
23565	M_HDL_P	Concentration of medium HDL particles	Lipoprotein subclasses	Medium HDL (average diameter 10.9 nm)	mmol/L	NA	NA
23566	M_HDL_L	Total lipids in medium HDL	Lipoprotein subclasses	Medium HDL (average diameter 10.9 nm)	mmol/L	NA	NA
23567	M_HDL_PL	Phospholipids in medium HDL	Lipoprotein subclasses	Medium HDL (average diameter 10.9 nm)	mmol/L	NA	NA
23568	M_HDL_C	Cholesterol in medium HDL	Lipoprotein subclasses	Medium HDL (average diameter 10.9 nm)	mmol/L	NA	NA
23569	M_HDL_CE	Cholesteryl esters in medium HDL	Lipoprotein subclasses	Medium HDL (average diameter 10.9 nm)	mmol/L	NA	NA
23570	M_HDL_FC	Free cholesterol in medium HDL	Lipoprotein subclasses	Medium HDL (average diameter 10.9 nm)	mmol/L	NA	NA
23571	M_HDL_TG	Triglycerides in medium HDL	Lipoprotein subclasses	Medium HDL (average diameter 10.9 nm)	mmol/L	NA	NA
23572	S_HDL_P	Concentration of small HDL particles	Lipoprotein subclasses	Small HDL (average diameter 8.7 nm)	mmol/L	NA	NA
23573	S_HDL_L	Total lipids in small HDL	Lipoprotein subclasses	Small HDL (average diameter 8.7 nm)	mmol/L	NA	NA
23574	S_HDL_PL	Phospholipids in small HDL	Lipoprotein subclasses	Small HDL (average diameter 8.7 nm)	mmol/L	NA	NA
23575	S_HDL_C	Cholesterol in small HDL	Lipoprotein subclasses	Small HDL (average diameter 8.7 nm)	mmol/L	NA	NA
23576	S_HDL_CE	Cholesteryl esters in small HDL	Lipoprotein subclasses	Small HDL (average diameter 8.7 nm)	mmol/L	NA	NA
23577	S_HDL_FC	Free cholesterol in small HDL	Lipoprotein subclasses	Small HDL (average diameter 8.7 nm)	mmol/L	NA	NA
23578	S_HDL_TG	Triglycerides in small HDL	Lipoprotein subclasses	Small HDL (average diameter 8.7 nm)	mmol/L	NA	NA

Supplementary Table 6. Distribution of metabolites and associations of the healthy lifestyle score with the metabolites at baseline and first repeat assessment visit.

		At baseline			First repeat assessment visit		
**Metabolic biomarkers**	**Unit**	**Mean (SD)**	**Beta (SE) †**	***P* value ***	**Mean (SD)**	**Beta (SE) †**	***P* value ***
Amino acids							
Spectrometer-corrected alanine	mmol/L	0.02253 (0.99171)	0.00565 (0.00178)	0.366	-0.00103 (0.995)	0.00427 (0.0078)	1
Alanine	mmol/L	0.02356 (0.99417)	0.00577 (0.0018)	0.342	0.00404 (0.9986)	-0.00279 (0.0079)	1
Glutamine	mmol/L	-0.00488 (0.98633)	0.02956 (0.0018)	< 0.001	-0.00037 (0.99863)	0.03398 (0.00792)	0.005
Glycine	mmol/L	-0.03038 (0.97862)	0.02366 (0.00168)	< 0.001	-0.04349 (0.97762)	0.02895 (0.0072)	0.015
Histidine	mmol/L	0.01902 (0.98239)	0.01513 (0.00181)	< 0.001	0.00125 (0.97241)	0.01203 (0.00786)	1
Total concentration of branched-chain amino acids (leucine + isoleucine + valine)	mmol/L	0.03702 (0.99575)	-0.00019 (0.00175)	1	0.03221 (0.9994)	0.00757 (0.00774)	1
Isoleucine	mmol/L	0.03039 (0.99947)	-0.0014 (0.0018)	1	0.02246 (1.00585)	0.01007 (0.00795)	1
Leucine	mmol/L	0.04098 (0.99772)	-0.00224 (0.00175)	1	0.03894 (1.00446)	0.00393 (0.00776)	1
Valine	mmol/L	0.03311 (0.99428)	0.00146 (0.00175)	1	0.02861 (0.99611)	0.00911 (0.00771)	1
Phenylalanine	mmol/L	0.00091 (0.96327)	-0.01018 (0.00177)	< 0.001	-0.01158 (0.9861)	0.0028 (0.00801)	1
Tyrosine	mmol/L	0.00824 (0.99175)	-0.01555 (0.00181)	< 0.001	0.00076 (0.98869)	-0.01112 (0.00786)	1
Apolipoproteins							
Apolipoprotein B	g/l	0.02809 (0.98299)	-0.0173 (0.00166)	< 0.001	0.0343 (0.99456)	-0.00931 (0.00742)	1
Apolipoprotein A1	g/l	-0.02242 (0.97604)	-0.01559 (0.00163)	< 0.001	-0.0346 (0.98496)	-0.02985 (0.00701)	0.005
Ratio of apolipoprotein B to apolipoprotein A1	ratio	0.03391 (0.97986)	-0.00847 (0.00167)	< 0.001	0.04932 (0.99056)	0.00806 (0.0076)	1
Cholesterol							
Total cholesterol	mmol/L	0.00939 (0.9869)	-0.01036 (0.00161)	< 0.001	0.00844 (0.99852)	-0.01178 (0.00705)	1
Total cholesterol minus HDL-C	mmol/L	0.02325 (0.98426)	-0.01624 (0.00164)	< 0.001	0.02663 (0.9961)	-0.01181 (0.0073)	1
Rem nt cholesterol (non-HDL, non-LDL -cholesterol)	mmol/L	0.02788 (0.98134)	-0.02106 (0.00162)	< 0.001	0.03201 (0.99279)	-0.01363 (0.00725)	1
VLDL cholesterol	mmol/L	0.04451 (0.96839)	-0.03409 (0.00167)	< 0.001	0.05121 (0.97631)	-0.02055 (0.00746)	1
Clinical LDL cholesterol	mmol/L	0.01562 (0.98684)	-0.00638 (0.00164)	0.026	0.01987 (0.99866)	-0.00665 (0.00731)	1
LDL cholesterol	mmol/L	0.01749 (0.98683)	-0.01157 (0.00168)	< 0.001	0.021 (0.99861)	-0.01046 (0.00742)	1
HDL cholesterol	mmol/L	-0.0284 (0.9723)	0.01359 (0.00154)	< 0.001	-0.0392 (0.97803)	-0.00432 (0.00668)	1
Cholesteryl esters							
Total esterified cholesterol	mmol/L	0.00619 (0.98707)	-0.00904 (0.00161)	< 0.001	0.00512 (0.99805)	-0.01207 (0.00703)	1
Cholesteryl esters in VLDL	mmol/L	0.0397 (0.97332)	-0.02656 (0.00166)	< 0.001	0.04695 (0.98236)	-0.01471 (0.00742)	1
Cholesteryl esters in LDL	mmol/L	0.02069 (0.98634)	-0.01701 (0.00169)	< 0.001	0.02425 (0.99758)	-0.0142 (0.00747)	1
Cholesteryl esters in HDL	mmol/L	-0.02872 (0.97166)	0.01579 (0.00155)	< 0.001	-0.03946 (0.97684)	-0.00372 (0.00673)	1
Fatty acids							
Total fatty acids	mmol/L	0.02304 (0.98056)	-0.06136 (0.00174)	< 0.001	0.02192 (0.99082)	-0.05049 (0.00766)	< 0.001
Degree of unsaturation	degree	-0.01624 (0.97925)	0.11668 (0.00166)	< 0.001	-0.02672 (0.97143)	0.08947 (0.00728)	< 0.001
Omega-3 fatty acids	mmol/L	0.0178 (0.98799)	0.04596 (0.00176)	< 0.001	-0.00305 (0.98802)	0.02381 (0.00761)	0.442
Omega-6 fatty acids	mmol/L	0.014 (0.98494)	-0.01898 (0.0017)	< 0.001	0.01597 (0.99351)	-0.00778 (0.00734)	1
Polyunsaturated fatty acids	mmol/L	0.01651 (0.98497)	-0.00355 (0.00172)	1	0.01261 (0.99327)	-0.00003 (0.00737)	1
Monounsaturated fatty acids	mmol/L	0.0276 (0.97655)	-0.08608 (0.00172)	< 0.001	0.03283 (0.98258)	-0.06933 (0.00768)	< 0.001
Saturated fatty acids	mmol/L	0.02118 (0.98235)	-0.07899 (0.00174)	< 0.001	0.01854 (0.99244)	-0.07149 (0.00771)	< 0.001
Linoleic acid	mmol/L	0.0196 (0.98423)	-0.00506 (0.00168)	0.676	0.02053 (0.99147)	0.01289 (0.0073)	1
Docosahexaenoic acid	mmol/L	0.00596 (0.9889)	0.07263 (0.00173)	< 0.001	-0.01541 (0.98396)	0.04231 (0.00745)	< 0.001
Ratio of omega-3 fatty acids to total fatty acids	%	0.01243 (0.98015)	0.09298 (0.00172)	< 0.001	-0.01342 (0.9833)	0.05909 (0.00759)	< 0.001
Ratio of omega-6 fatty acids to total fatty acids	%	-0.02412 (0.98463)	0.09177 (0.00168)	< 0.001	-0.01843 (0.98291)	0.09048 (0.00747)	< 0.001
Ratio of polyunsaturated fatty acids to total fatty acids	%	-0.02042 (0.98176)	0.12413 (0.00166)	< 0.001	-0.02488 (0.97853)	0.11189 (0.00739)	< 0.001
Ratio of monounsaturated fatty acids to total fatty acids	%	0.0316 (0.96458)	-0.10168 (0.0016)	< 0.001	0.04404 (0.96229)	-0.077 (0.00706)	< 0.001
Ratio of saturated fatty acids to total fatty acids	%	0.00677 (0.98484)	-0.09853 (0.00177)	< 0.001	-0.00227 (0.99503)	-0.10513 (0.0079)	< 0.001
Ratio of linoleic acid to total fatty acids	%	-0.00435 (0.98893)	0.08981 (0.00162)	< 0.001	0.00082 (0.98711)	0.10179 (0.00709)	< 0.001
Ratio of docosahexaenoic acid to total fatty acids	%	-0.00606 (0.97836)	0.11261 (0.00169)	< 0.001	-0.02837 (0.97822)	0.07681 (0.00752)	< 0.001
Ratio of polyunsaturated fatty acids to monounsaturated fatty acids	ratio	-0.02994 (0.97035)	0.11559 (0.00161)	< 0.001	-0.03961 (0.96645)	0.09599 (0.00713)	< 0.001
Ratio of omega-6 fatty acids to omega-3 fatty acids	ratio	-0.01945 (0.98044)	-0.06194 (0.00174)	< 0.001	0.00611 (0.98562)	-0.03141 (0.00763)	0.01
Fluid balance							
Creatinine	mmol/L	0.03293 (0.93863)	-0.01291 (0.00148)	< 0.001	0.02644 (0.9533)	-0.0128 (0.00655)	1
Albumin	g/l	0.03685 (0.98042)	0.0356 (0.00179)	< 0.001	0.02372 (0.98357)	0.01424 (0.00782)	1
Free cholesterol							
Total free cholesterol	mmol/L	0.01648 (0.9869)	-0.01421 (0.00162)	< 0.001	0.01708 (0.99858)	-0.01187 (0.00714)	1
Free cholesterol in VLDL	mmol/L	0.04532 (0.96948)	-0.04376 (0.00168)	< 0.001	0.05195 (0.97597)	-0.02798 (0.00757)	0.055
Free cholesterol in LDL	mmol/L	0.00706 (0.9889)	0.00208 (0.00166)	1	0.01207 (1.00035)	-0.00153 (0.00734)	1
Free cholesterol in HDL	mmol/L	-0.02891 (0.96921)	0.00401 (0.00154)	1	-0.0388 (0.97541)	-0.0079 (0.00654)	1
Glycolysis related metabolites							
Glucose-lactate	mmol/L	-0.014 (0.97062)	-0.02556 (0.00164)	< 0.001	-0.01564 (0.97775)	-0.04382 (0.00733)	< 0.001
Glucose	mmol/L	-0.02038 (0.97631)	-0.01053 (0.00169)	< 0.001	-0.01192 (0.9748)	-0.0218 (0.00735)	0.761
Lactate	mmol/L	0.01435 (1.0005)	-0.03146 (0.00184)	< 0.001	-0.01057 (0.98641)	-0.06651 (0.00776)	< 0.001
Pyruvate	mmol/L	0.00699 (0.96649)	-0.02397 (0.00178)	< 0.001	-0.02518 (0.99425)	-0.07052 (0.00774)	< 0.001
Citrate	mmol/L	-0.01618 (0.99046)	0.03147 (0.00179)	< 0.001	-0.01618 (0.99306)	0.01184 (0.00782)	1
Inflammation							
Glycoprotein acetyls	mmol/L	-0.0183 (0.97918)	-0.08897 (0.00171)	< 0.001	-0.00683 (0.98027)	-0.06647 (0.00756)	< 0.001
Ketone bodies							
3-Hydroxybutyrate	mmol/L	-0.00273 (0.98765)	-0.03643 (0.00182)	< 0.001	0.00862 (1.00032)	-0.04941 (0.00791)	< 0.001
Acetate	mmol/L	-0.05346 (0.4098)	0.00831 (0.00076)	< 0.001	-0.02812 (0.59429)	0.01969 (0.00468)	0.006
Acetoacetate	mmol/L	0.00399 (0.98554)	-0.0393 (0.00182)	< 0.001	0.0196 (1.00834)	-0.03438 (0.00796)	0.004
Acetone	mmol/L	0.00409 (0.95805)	-0.02149 (0.00177)	< 0.001	0.00823 (0.98471)	-0.02958 (0.00779)	0.037
Lipoprotein particle concentrations							
Total concentration of lipoprotein particles	mmol/L	-0.01371 (0.97865)	-0.0269 (0.00168)	< 0.001	-0.02136 (0.98427)	-0.03972 (0.00722)	< 0.001
Concentration of VLDL particles	mmol/L	0.03779 (0.97486)	-0.0416 (0.00171)	< 0.001	0.04407 (0.98299)	-0.02618 (0.00766)	0.159
Concentration of LDL particles	mmol/L	0.02599 (0.98617)	-0.01458 (0.00168)	< 0.001	0.03386 (0.99797)	-0.00691 (0.00752)	1
Concentration of HDL particles	mmol/L	-0.0184 (0.97552)	-0.02514 (0.00168)	< 0.001	-0.02761 (0.98045)	-0.04014 (0.00724)	< 0.001
Lipoprotein particle sizes							
Average diameter for VLDL particles	nm	0.0644 (0.95114)	-0.04959 (0.00157)	< 0.001	0.06452 (0.9492)	-0.03502 (0.00692)	< 0.001
Average diameter for LDL particles	nm	-0.01213 (0.99515)	0.02532 (0.00174)	< 0.001	0.00045 (0.99332)	0.0195 (0.00749)	1
Average diameter for HDL particles	nm	-0.02878 (0.97748)	0.042 (0.00151)	< 0.001	-0.04047 (0.97543)	0.03125 (0.00649)	< 0.001
Lipoprotein subclasses							
Concentration of chylomicrons and extremely large VLDL particles	mmol/L	0.03183 (0.98278)	-0.06887 (0.00171)	< 0.001	0.03571 (0.98628)	-0.04748 (0.0076)	< 0.001
Total lipids in chylomicrons and extremely large VLDL	mmol/L	0.038 (0.97919)	-0.06833 (0.00169)	< 0.001	0.04076 (0.98184)	-0.0483 (0.00753)	< 0.001
Phospholipids in chylomicrons and extremely large VLDL	mmol/L	0.03348 (0.98122)	-0.06861 (0.0017)	< 0.001	0.03754 (0.986)	-0.04769 (0.00756)	< 0.001
Cholesterol in chylomicrons and extremely large VLDL	mmol/L	0.03955 (0.97618)	-0.06697 (0.00169)	< 0.001	0.04383 (0.98258)	-0.04888 (0.00755)	< 0.001
Cholesteryl esters in chylomicrons and extremely large VLDL	mmol/L	0.03899 (0.97781)	-0.06586 (0.00169)	< 0.001	0.04319 (0.98527)	-0.0496 (0.00757)	< 0.001
Free cholesterol in chylomicrons and extremely large VLDL	mmol/L	0.03784 (0.97691)	-0.06749 (0.0017)	< 0.001	0.04234 (0.9825)	-0.04704 (0.00756)	< 0.001
Triglycerides in chylomicrons and extremely large VLDL	mmol/L	0.03349 (0.98409)	-0.06827 (0.0017)	< 0.001	0.03582 (0.98548)	-0.04723 (0.00759)	< 0.001
Concentration of very large VLDL particles	mmol/L	0.04043 (0.97681)	-0.06249 (0.00168)	< 0.001	0.04356 (0.97728)	-0.0433 (0.00752)	< 0.001
Total lipids in very large VLDL	mmol/L	0.04694 (0.97134)	-0.06139 (0.00166)	< 0.001	0.04966 (0.97084)	-0.04264 (0.00744)	< 0.001
Phospholipids in very large VLDL	mmol/L	0.04307 (0.97429)	-0.0605 (0.00168)	< 0.001	0.04773 (0.97612)	-0.04072 (0.00752)	< 0.001
Cholesterol in very large VLDL	mmol/L	0.04634 (0.97098)	-0.05228 (0.00167)	< 0.001	0.05307 (0.97477)	-0.03464 (0.00753)	0.001
Cholesteryl esters in very large VLDL	mmol/L	0.04538 (0.97244)	-0.04546 (0.00167)	< 0.001	0.05339 (0.9772)	-0.03013 (0.00754)	0.016
Free cholesterol in very large VLDL	mmol/L	0.04477 (0.97217)	-0.05774 (0.00168)	< 0.001	0.04996 (0.97543)	-0.03812 (0.00754)	< 0.001
Triglycerides in very large VLDL	mmol/L	0.04316 (0.97591)	-0.06392 (0.00167)	< 0.001	0.04374 (0.97405)	-0.04489 (0.00745)	< 0.001
Concentration of large VLDL particles	mmol/L	0.04334 (0.97552)	-0.05506 (0.00169)	< 0.001	0.04614 (0.97708)	-0.03741 (0.00756)	< 0.001
Total lipids in large VLDL	mmol/L	0.05229 (0.96781)	-0.05173 (0.00166)	< 0.001	0.054 (0.96756)	-0.03515 (0.00746)	< 0.001
Phospholipids in large VLDL	mmol/L	0.04482 (0.9734)	-0.05726 (0.00167)	< 0.001	0.04878 (0.97318)	-0.03866 (0.00749)	< 0.001
Cholesterol in large VLDL	mmol/L	0.04834 (0.96875)	-0.0459 (0.00168)	< 0.001	0.05414 (0.97165)	-0.02944 (0.00753)	0.023
Cholesteryl esters in large VLDL	mmol/L	0.0486 (0.96751)	-0.03636 (0.00169)	< 0.001	0.05543 (0.97229)	-0.02112 (0.00756)	1
Free cholesterol in large VLDL	mmol/L	0.04525 (0.97324)	-0.05392 (0.00167)	< 0.001	0.04989 (0.97484)	-0.03655 (0.00751)	< 0.001
Triglycerides in large VLDL	mmol/L	0.04996 (0.97366)	-0.05158 (0.00168)	< 0.001	0.04849 (0.97296)	-0.03559 (0.00752)	< 0.001
Concentration of medium VLDL particles	mmol/L	0.04191 (0.97712)	-0.02933 (0.0017)	< 0.001	0.04835 (0.98629)	-0.01768 (0.00763)	1
Total lipids in medium VLDL	mmol/L	0.05005 (0.96888)	-0.03408 (0.00169)	< 0.001	0.05324 (0.97476)	-0.02123 (0.00757)	1
Phospholipids in medium VLDL	mmol/L	0.04158 (0.97611)	-0.0283 (0.00169)	< 0.001	0.04769 (0.98471)	-0.01621 (0.00757)	1
Cholesterol in medium VLDL	mmol/L	0.03155 (0.983)	-0.00234 (0.00165)	1	0.03898 (0.99243)	0.0033 (0.00733)	1
Cholesteryl esters in medium VLDL	mmol/L	0.02324 (0.98723)	0.01022 (0.00163)	< 0.001	0.03145 (0.99526)	0.01218 (0.0072)	1
Free cholesterol in medium VLDL	mmol/L	0.03882 (0.97866)	-0.01884 (0.00168)	< 0.001	0.0455 (0.98816)	-0.00911 (0.00753)	1
Triglycerides in medium VLDL	mmol/L	0.05029 (0.97143)	-0.0478 (0.0017)	< 0.001	0.04969 (0.97196)	-0.03319 (0.00757)	0.003
Concentration of small VLDL particles	mmol/L	0.03988 (0.97524)	-0.04022 (0.00172)	< 0.001	0.04538 (0.97939)	-0.02359 (0.0077)	0.549
Total lipids in small VLDL	mmol/L	0.04382 (0.97098)	-0.03985 (0.00171)	< 0.001	0.04891 (0.97464)	-0.02478 (0.00762)	0.289
Phospholipids in small VLDL	mmol/L	0.03831 (0.97692)	-0.02958 (0.00171)	< 0.001	0.04525 (0.98423)	-0.01712 (0.00762)	1
Cholesterol in small VLDL	mmol/L	0.03675 (0.97755)	-0.02633 (0.0017)	< 0.001	0.04533 (0.98484)	-0.01501 (0.00757)	1
Cholesteryl esters in small VLDL	mmol/L	0.03683 (0.97645)	-0.03036 (0.0017)	< 0.001	0.04614 (0.98298)	-0.01789 (0.0076)	1
Free cholesterol in small VLDL	mmol/L	0.03431 (0.98142)	-0.01903 (0.0017)	< 0.001	0.04178 (0.99022)	-0.00992 (0.00755)	1
Triglycerides in small VLDL	mmol/L	0.04066 (0.97316)	-0.04936 (0.00171)	< 0.001	0.04077 (0.97356)	-0.03213 (0.00761)	0.006
Concentration of very small VLDL particles	mmol/L	0.01922 (0.98111)	-0.03007 (0.00168)	< 0.001	0.02504 (0.99318)	-0.01839 (0.00739)	1
Total lipids in very small VLDL	mmol/L	0.02225 (0.97852)	-0.03131 (0.00168)	< 0.001	0.02705 (0.98912)	-0.01855 (0.00732)	1
Phospholipids in very small VLDL	mmol/L	0.02139 (0.97687)	-0.0349 (0.0017)	< 0.001	0.02814 (0.98802)	-0.02044 (0.00741)	1
Cholesterol in very small VLDL	mmol/L	0.01719 (0.9843)	-0.01082 (0.00161)	< 0.001	0.02253 (0.99333)	-0.00421 (0.00704)	1
Cholesteryl esters in very small VLDL	mmol/L	0.01489 (0.9863)	-0.00514 (0.00158)	0.293	0.01997 (0.99474)	-0.00034 (0.00695)	1
Free cholesterol in very small VLDL	mmol/L	0.02083 (0.97988)	-0.02405 (0.00167)	< 0.001	0.02738 (0.9901)	-0.01392 (0.00729)	1
Triglycerides in very small VLDL	mmol/L	0.0221 (0.97882)	-0.06114 (0.00175)	< 0.001	0.02322 (0.98392)	-0.04499 (0.00776)	< 0.001
Concentration of IDL particles	mmol/L	0.01418 (0.98747)	-0.01503 (0.00163)	< 0.001	0.02017 (0.99897)	-0.01174 (0.00725)	1
Total lipids in IDL	mmol/L	0.00591 (0.98792)	-0.00833 (0.00158)	< 0.001	0.00897 (0.99821)	-0.00719 (0.00695)	1
Phospholipids in IDL	mmol/L	0.00783 (0.98768)	-0.00578 (0.00159)	0.067	0.01295 (0.99796)	-0.00314 (0.00695)	1
Cholesterol in IDL	mmol/L	0.00398 (0.98819)	-0.00129 (0.00156)	1	0.00718 (0.9975)	-0.00349 (0.00693)	1
Cholesteryl esters in IDL	mmol/L	0.0037 (0.98793)	-0.00386 (0.00157)	1	0.00659 (0.99769)	-0.00549 (0.00694)	1
Free cholesterol in IDL	mmol/L	0.00332 (0.98894)	0.00479 (0.00158)	0.621	0.00841 (0.9968)	0.00029 (0.007)	1
Triglycerides in IDL	mmol/L	0.00885 (0.98562)	-0.0666 (0.00177)	< 0.001	0.00878 (0.99522)	-0.05117 (0.0078)	< 0.001
Concentration of large LDL particles	mmol/L	0.02217 (0.98766)	-0.0101 (0.00168)	< 0.001	0.03213 (0.99955)	-0.00266 (0.00747)	1
Total lipids in large LDL	mmol/L	0.01187 (0.98774)	-0.01158 (0.00167)	< 0.001	0.0157 (0.99976)	-0.01044 (0.00735)	1
Phospholipids in large LDL	mmol/L	0.01205 (0.98927)	-0.01243 (0.00166)	< 0.001	0.01742 (1.00029)	-0.0126 (0.00738)	1
Cholesterol in large LDL	mmol/L	0.01165 (0.98734)	-0.00543 (0.00166)	0.268	0.01579 (0.99946)	-0.00572 (0.00732)	1
Cholesteryl esters in large LDL	mmol/L	0.01382 (0.98717)	-0.00893 (0.00168)	< 0.001	0.01827 (0.99911)	-0.00766 (0.00738)	1
Free cholesterol in large LDL	mmol/L	0.00404 (0.98852)	0.00285 (0.00163)	1	0.00889 (0.99972)	-0.00186 (0.00722)	1
Triglycerides in large LDL	mmol/L	0.00221 (0.99)	-0.07347 (0.00177)	< 0.001	0.00107 (0.99929)	-0.06015 (0.00787)	< 0.001
Concentration of medium LDL particles	mmol/L	0.0323 (0.98563)	-0.02087 (0.0017)	< 0.001	0.03546 (0.99464)	-0.01551 (0.00764)	1
Total lipids in medium LDL	mmol/L	0.02463 (0.9879)	-0.0286 (0.00171)	< 0.001	0.02837 (0.99771)	-0.02428 (0.00765)	0.378
Phospholipids in medium LDL	mmol/L	0.0191 (0.99025)	-0.0275 (0.00172)	< 0.001	0.02502 (1.0008)	-0.02304 (0.0077)	0.7
Cholesterol in medium LDL	mmol/L	0.02614 (0.98769)	-0.02447 (0.00171)	< 0.001	0.02973 (0.99718)	-0.02154 (0.00763)	1
Cholesteryl esters in medium LDL	mmol/L	0.03066 (0.98658)	-0.03179 (0.00171)	< 0.001	0.03361 (0.99478)	-0.0273 (0.00765)	0.09
Free cholesterol in medium LDL	mmol/L	0.01102 (0.98976)	-0.0037 (0.0017)	1	0.01735 (1.00121)	-0.00548 (0.00758)	1
Triglycerides in medium LDL	mmol/L	0.01217 (0.98886)	-0.07182 (0.00176)	< 0.001	0.01194 (0.99619)	-0.05794 (0.00789)	< 0.001
Concentration of small LDL particles	mmol/L	0.02619 (0.98577)	-0.02023 (0.00169)	< 0.001	0.03235 (0.99751)	-0.00858 (0.00762)	1
Total lipids in small LDL	mmol/L	0.02337 (0.98906)	-0.0238 (0.0017)	< 0.001	0.02855 (1.00122)	-0.0158 (0.00764)	1
Phospholipids in small LDL	mmol/L	0.01636 (0.99074)	-0.01555 (0.00171)	< 0.001	0.02242 (1.00441)	-0.00656 (0.00765)	1
Cholesterol in small LDL	mmol/L	0.02369 (0.98905)	-0.02028 (0.0017)	< 0.001	0.02917 (1.00049)	-0.01502 (0.00761)	1
Cholesteryl esters in small LDL	mmol/L	0.02736 (0.98807)	-0.03018 (0.0017)	< 0.001	0.03261 (0.99876)	-0.02289 (0.00764)	0.688
Free cholesterol in small LDL	mmol/L	0.01093 (0.99005)	0.00749 (0.0017)	0.003	0.01746 (1.00266)	0.00638 (0.00758)	1
Triglycerides in small LDL	mmol/L	0.02676 (0.98263)	-0.06654 (0.00174)	< 0.001	0.02757 (0.98769)	-0.04845 (0.00779)	< 0.001
Concentration of very large HDL particles	mmol/L	-0.02836 (0.96006)	0.03888 (0.0015)	< 0.001	-0.03469 (0.95952)	0.02964 (0.00626)	< 0.001
Total lipids in very large HDL	mmol/L	-0.02909 (0.96649)	0.04398 (0.00149)	< 0.001	-0.03488 (0.96371)	0.03391 (0.00631)	< 0.001
Phospholipids in very large HDL	mmol/L	-0.02984 (0.96608)	0.04345 (0.00151)	< 0.001	-0.03631 (0.9631)	0.03327 (0.00635)	< 0.001
Cholesterol in very large HDL	mmol/L	-0.03033 (0.96275)	0.04716 (0.00148)	< 0.001	-0.03513 (0.95961)	0.03504 (0.00627)	< 0.001
Cholesteryl esters in very large HDL	mmol/L	-0.0302 (0.96289)	0.04753 (0.00148)	< 0.001	-0.03596 (0.96037)	0.03398 (0.00625)	< 0.001
Free cholesterol in very large HDL	mmol/L	-0.0304 (0.96006)	0.04303 (0.00153)	< 0.001	-0.03201 (0.95492)	0.03667 (0.0064)	< 0.001
Triglycerides in very large HDL	mmol/L	0.01117 (0.97962)	-0.03699 (0.00178)	< 0.001	0.00435 (0.98968)	-0.01895 (0.0077)	1
Concentration of large HDL particles	mmol/L	-0.03189 (0.96431)	0.03335 (0.00149)	< 0.001	-0.04121 (0.96572)	0.01841 (0.00635)	0.937
Total lipids in large HDL	mmol/L	-0.03065 (0.97509)	0.03206 (0.00149)	< 0.001	-0.04067 (0.97656)	0.01818 (0.00644)	1
Phospholipids in large HDL	mmol/L	-0.03251 (0.97246)	0.02486 (0.00151)	< 0.001	-0.04307 (0.97477)	0.01137 (0.00649)	1
Cholesterol in large HDL	mmol/L	-0.0312 (0.97085)	0.04038 (0.00148)	< 0.001	-0.03993 (0.97074)	0.02436 (0.00638)	0.034
Cholesteryl esters in large HDL	mmol/L	-0.0314 (0.96964)	0.0421 (0.00148)	< 0.001	-0.04017 (0.96937)	0.02512 (0.00639)	0.022
Free cholesterol in large HDL	mmol/L	-0.03267 (0.96629)	0.03221 (0.00149)	< 0.001	-0.04108 (0.96714)	0.01908 (0.00634)	0.652
Triglycerides in large HDL	mmol/L	-0.0023 (0.98044)	-0.02733 (0.00176)	< 0.001	-0.01276 (0.98928)	-0.01937 (0.00746)	1
Concentration of medium HDL particles	mmol/L	-0.02397 (0.9745)	-0.0197 (0.00164)	< 0.001	-0.03558 (0.98038)	-0.0349 (0.0071)	< 0.001
Total lipids in medium HDL	mmol/L	-0.02077 (0.9801)	-0.02524 (0.00167)	< 0.001	-0.03358 (0.9891)	-0.03971 (0.00724)	< 0.001
Phospholipids in medium HDL	mmol/L	-0.0187 (0.98172)	-0.03518 (0.00169)	< 0.001	-0.0309 (0.99029)	-0.04723 (0.00732)	< 0.001
Cholesterol in medium HDL	mmol/L	-0.02681 (0.97427)	-0.0087 (0.00163)	< 0.001	-0.03882 (0.98083)	-0.02742 (0.00709)	0.028
Cholesteryl esters in medium HDL	mmol/L	-0.02743 (0.97354)	-0.00794 (0.00163)	< 0.001	-0.03937 (0.97958)	-0.02768 (0.00713)	0.026
Free cholesterol in medium HDL	mmol/L	-0.02576 (0.97326)	-0.01407 (0.00161)	< 0.001	-0.03696 (0.97959)	-0.02794 (0.00694)	0.014
Triglycerides in medium HDL	mmol/L	0.02021 (0.97828)	-0.0682 (0.00176)	< 0.001	0.01816 (0.9793)	-0.0577 (0.00768)	< 0.001
Concentration of small HDL particles	mmol/L	0.00312 (0.9842)	-0.05479 (0.00179)	< 0.001	0.001 (0.98091)	-0.06413 (0.00775)	< 0.001
Total lipids in small HDL	mmol/L	0.00528 (0.98562)	-0.06894 (0.00179)	< 0.001	0.00132 (0.9828)	-0.07501 (0.0077)	< 0.001
Phospholipids in small HDL	mmol/L	0.00348 (0.98589)	-0.0726 (0.00178)	< 0.001	-0.00176 (0.98425)	-0.07856 (0.00769)	< 0.001
Cholesterol in small HDL	mmol/L	-0.00072 (0.98321)	-0.04775 (0.0018)	< 0.001	-0.00338 (0.98146)	-0.05996 (0.00775)	< 0.001
Cholesteryl esters in small HDL	mmol/L	0.00047 (0.98174)	-0.0438 (0.0018)	< 0.001	-0.0007 (0.97931)	-0.05772 (0.00776)	< 0.001
Free cholesterol in small HDL	mmol/L	-0.00462 (0.98496)	-0.05332 (0.00177)	< 0.001	-0.01137 (0.98609)	-0.05894 (0.00762)	< 0.001
Triglycerides in small HDL	mmol/L	0.02911 (0.9761)	-0.07334 (0.00168)	< 0.001	0.03116 (0.97158)	-0.0573 (0.0074)	< 0.001
Other lipids							
Phosphoglycerides	mmol/L	0.00138 (0.98494)	-0.03657 (0.00168)	< 0.001	-0.00509 (0.99667)	-0.03687 (0.0072)	< 0.001
Ratio of triglycerides to phosphoglycerides	ratio	0.04741 (0.97064)	-0.05271 (0.00159)	< 0.001	0.04953 (0.96743)	-0.03263 (0.00707)	0.001
Total cholines	mmol/L	-0.00009 (0.98497)	-0.02917 (0.00165)	< 0.001	-0.006 (0.99625)	-0.02993 (0.00707)	0.006
Phosphatidylcholines	mmol/L	0.00201 (0.98395)	-0.02759 (0.00166)	< 0.001	-0.00567 (0.99579)	-0.02849 (0.0071)	0.015
Sphingomyelins	mmol/L	-0.01396 (0.98488)	-0.01816 (0.00161)	< 0.001	-0.01463 (0.99109)	-0.02422 (0.00691)	0.115
Phospholipids							
Total phospholipids in lipoprotein particles	mmol/L	0.00534 (0.98499)	-0.03504 (0.00165)	< 0.001	0.00012 (0.99752)	-0.03357 (0.00712)	< 0.001
Phospholipids in VLDL	mmol/L	0.04581 (0.96799)	-0.04976 (0.00169)	< 0.001	0.05197 (0.97227)	-0.0326 (0.00757)	0.004
Phospholipids in LDL	mmol/L	0.01543 (0.98916)	-0.01725 (0.00168)	< 0.001	0.02062 (1.001)	-0.01477 (0.0075)	1
Phospholipids in HDL	mmol/L	-0.02366 (0.97799)	-0.01421 (0.00161)	< 0.001	-0.03648 (0.98717)	-0.02623 (0.00693)	0.039
Relative lipoprotein lipid concentrations							
Phospholipids to total lipids ratio in chylomicrons and extremely large VLDL	%	0.00908 (0.9735)	-0.01777 (0.00176)	< 0.001	0.01389 (0.96529)	-0.01519 (0.00725)	1
Cholesterol to total lipids ratio in chylomicrons and extremely large VLDL	%	0.0137 (1.00376)	0.02701 (0.00183)	< 0.001	0.0122 (1.0018)	0.01442 (0.00777)	1
Cholesteryl esters to total lipids ratio in chylomicrons and extremely large VLDL	%	0.01047 (1.0013)	0.01799 (0.00184)	< 0.001	0.01055 (1.00104)	0.0031 (0.00779)	1
Free cholesterol to total lipids ratio in chylomicrons and extremely large VLDL	%	0.01691 (1.00606)	0.03431 (0.00182)	< 0.001	0.01292 (0.9984)	0.02665 (0.00765)	0.124
Triglycerides to total lipids ratio in chylomicrons and extremely large VLDL	%	-0.00992 (1.01646)	-0.01172 (0.00187)	< 0.001	-0.00472 (0.99317)	-0.00597 (0.00775)	1
Phospholipids to total lipids ratio in very large VLDL	%	0.0331 (0.91821)	-0.00827 (0.00168)	< 0.001	0.04306 (0.88662)	-0.0042 (0.0065)	1
Cholesterol to total lipids ratio in very large VLDL	%	-0.05901 (0.92574)	0.05108 (0.00155)	< 0.001	-0.05342 (0.92769)	0.03721 (0.00663)	< 0.001
Cholesteryl esters to total lipids ratio in very large VLDL	%	-0.06052 (0.9259)	0.05161 (0.00155)	< 0.001	-0.05602 (0.92771)	0.0362 (0.00671)	< 0.001
Free cholesterol to total lipids ratio in very large VLDL	%	-0.01814 (0.9364)	0.04099 (0.00162)	< 0.001	-0.01329 (0.92329)	0.03268 (0.00657)	< 0.001
Triglycerides to total lipids ratio in very large VLDL	%	0.06676 (0.76389)	-0.02998 (0.00132)	< 0.001	0.05889 (0.79866)	-0.02682 (0.00581)	< 0.001
Phospholipids to total lipids ratio in large VLDL	%	0.06386 (0.75905)	-0.02531 (0.00136)	< 0.001	0.07262 (0.75259)	-0.01907 (0.00546)	0.12
Cholesterol to total lipids ratio in large VLDL	%	-0.01654 (0.92351)	0.0317 (0.00163)	< 0.001	-0.00417 (0.9323)	0.02518 (0.00688)	0.064
Cholesteryl esters to total lipids ratio in large VLDL	%	-0.02191 (0.94316)	0.04836 (0.00158)	< 0.001	-0.01352 (0.94854)	0.03897 (0.00682)	< 0.001
Free cholesterol to total lipids ratio in large VLDL	%	0.00732 (0.88746)	-0.01574 (0.00161)	< 0.001	0.02433 (0.89135)	-0.01304 (0.00662)	1
Triglycerides to total lipids ratio in large VLDL	%	-0.00123 (0.88996)	0.00283 (0.00163)	1	-0.02064 (0.91267)	-0.0022 (0.00705)	1
Phospholipids to total lipids ratio in medium VLDL	%	-0.0126 (0.94546)	0.02206 (0.00152)	< 0.001	-0.00464 (0.97172)	0.02198 (0.00671)	0.267
Cholesterol to total lipids ratio in medium VLDL	%	-0.02259 (0.97699)	0.04746 (0.00155)	< 0.001	-0.01639 (0.97503)	0.03882 (0.00679)	< 0.001
Cholesteryl esters to total lipids ratio in medium VLDL	%	-0.01975 (0.97605)	0.04747 (0.00156)	< 0.001	-0.01417 (0.97108)	0.03881 (0.00685)	< 0.001
Free cholesterol to total lipids ratio in medium VLDL	%	-0.01725 (0.97445)	0.04031 (0.00156)	< 0.001	-0.0101 (0.97682)	0.0338 (0.0068)	< 0.001
Triglycerides to total lipids ratio in medium VLDL	%	0.03384 (0.96806)	-0.04235 (0.00155)	< 0.001	0.02609 (0.97262)	-0.034 (0.00675)	< 0.001
Phospholipids to total lipids ratio in small VLDL	%	-0.0206 (0.97315)	0.04028 (0.00158)	< 0.001	-0.01416 (0.97992)	0.02869 (0.00691)	0.008
Cholesterol to total lipids ratio in small VLDL	%	-0.01023 (0.9783)	0.03116 (0.00163)	< 0.001	-0.00031 (0.98366)	0.02083 (0.00716)	0.905
Cholesteryl esters to total lipids ratio in small VLDL	%	-0.00161 (0.96986)	0.01975 (0.00168)	< 0.001	0.0103 (0.98021)	0.01197 (0.00738)	1
Free cholesterol to total lipids ratio in small VLDL	%	-0.01776 (0.97638)	0.0413 (0.00157)	< 0.001	-0.01253 (0.98001)	0.0295 (0.00687)	0.004
Triglycerides to total lipids ratio in small VLDL	%	0.02071 (0.97362)	-0.03132 (0.00163)	< 0.001	0.00947 (0.9825)	-0.02032 (0.0071)	1
Phospholipids to total lipids ratio in very small VLDL	%	0.01459 (0.9567)	-0.02996 (0.00167)	< 0.001	0.02361 (0.96568)	-0.01449 (0.00741)	1
Cholesterol to total lipids ratio in very small VLDL	%	-0.00892 (0.98677)	0.05085 (0.00153)	< 0.001	-0.00371 (0.98464)	0.03918 (0.00684)	< 0.001
Cholesteryl esters to total lipids ratio in very small VLDL	%	-0.01029 (0.98504)	0.05032 (0.00152)	< 0.001	-0.00636 (0.98449)	0.03954 (0.0068)	< 0.001
Free cholesterol to total lipids ratio in very small VLDL	%	0.00769 (0.99163)	0.04128 (0.00171)	< 0.001	0.01976 (0.98563)	0.02889 (0.0075)	0.029
Triglycerides to total lipids ratio in very small VLDL	%	0.0117 (0.98841)	-0.04893 (0.00156)	< 0.001	0.00366 (0.98825)	-0.03698 (0.00687)	< 0.001
Phospholipids to total lipids ratio in IDL	%	0.0151 (0.97902)	0.01918 (0.00179)	< 0.001	0.02399 (0.9853)	0.02866 (0.00782)	0.063
Cholesterol to total lipids ratio in IDL	%	-0.00478 (0.98327)	0.04163 (0.00163)	< 0.001	-0.00452 (0.98156)	0.02808 (0.00722)	0.025
Cholesteryl esters to total lipids ratio in IDL	%	-0.00396 (0.97896)	0.02556 (0.00165)	< 0.001	-0.00692 (0.98075)	0.0168 (0.00726)	1
Free cholesterol to total lipids ratio in IDL	%	-0.00343 (0.99133)	0.05642 (0.00175)	< 0.001	0.00378 (0.98416)	0.03733 (0.00771)	< 0.001
Triglycerides to total lipids ratio in IDL	%	0.00513 (0.98689)	-0.05548 (0.0016)	< 0.001	0.00035 (0.98893)	-0.04047 (0.0071)	< 0.001
Phospholipids to total lipids ratio in large LDL	%	0.00344 (0.98937)	0.00215 (0.00182)	1	0.00834 (0.99648)	-0.00546 (0.00785)	1
Cholesterol to total lipids ratio in large LDL	%	0.00589 (0.98961)	0.03648 (0.00178)	< 0.001	0.00983 (1.00528)	0.04194 (0.00769)	< 0.001
Cholesteryl esters to total lipids ratio in large LDL	%	0.02057 (0.98693)	0.01842 (0.00182)	< 0.001	0.03372 (0.95903)	0.02562 (0.0077)	0.221
Free cholesterol to total lipids ratio in large LDL	%	-0.01669 (0.97454)	0.05133 (0.00162)	< 0.001	-0.0194 (0.99508)	0.03487 (0.00737)	< 0.001
Triglycerides to total lipids ratio in large LDL	%	-0.00897 (0.98542)	-0.06408 (0.00164)	< 0.001	-0.01557 (0.99137)	-0.04918 (0.00728)	< 0.001
Phospholipids to total lipids ratio in medium LDL	%	-0.04992 (0.97251)	0.02006 (0.00178)	< 0.001	-0.03818 (0.96213)	0.0198 (0.00758)	1
Cholesterol to total lipids ratio in medium LDL	%	0.02591 (1.0375)	0.02963 (0.00186)	< 0.001	0.03535 (0.96484)	0.02494 (0.00715)	0.122
Cholesteryl esters to total lipids ratio in medium LDL	%	0.06076 (0.96768)	-0.02812 (0.00174)	< 0.001	0.06389 (0.93348)	-0.03142 (0.00714)	0.003
Free cholesterol to total lipids ratio in medium LDL	%	-0.02985 (0.97006)	0.06439 (0.00167)	< 0.001	-0.02881 (0.97503)	0.05334 (0.00729)	< 0.001
Triglycerides to total lipids ratio in medium LDL	%	-0.01071 (0.98051)	-0.05419 (0.00167)	< 0.001	-0.01741 (0.98774)	-0.03866 (0.00737)	< 0.001
Phospholipids to total lipids ratio in small LDL	%	-0.03497 (0.97239)	0.04052 (0.00175)	< 0.001	-0.03467 (0.969)	0.0441 (0.00752)	< 0.001
Cholesterol to total lipids ratio in small LDL	%	0.01614 (1.05995)	0.01601 (0.00189)	< 0.001	0.02369 (0.97881)	-0.00107 (0.00742)	1
Cholesteryl esters to total lipids ratio in small LDL	%	0.03855 (1.01329)	-0.04242 (0.00183)	< 0.001	0.04348 (0.96579)	-0.04884 (0.00747)	< 0.001
Free cholesterol to total lipids ratio in small LDL	%	-0.01577 (0.97146)	0.06059 (0.00173)	< 0.001	-0.01622 (0.9668)	0.0513 (0.00738)	< 0.001
Triglycerides to total lipids ratio in small LDL	%	0.02238 (0.97461)	-0.05815 (0.00166)	< 0.001	0.01694 (0.9795)	-0.04324 (0.00734)	< 0.001
Phospholipids to total lipids ratio in very large HDL	%	-0.00701 (0.99108)	0.02091 (0.00176)	< 0.001	-0.00578 (0.97811)	0.0273 (0.00765)	0.09
Cholesterol to total lipids ratio in very large HDL	%	0.01072 (0.98339)	0.0001 (0.00173)	1	0.01629 (1.00912)	-0.01148 (0.00788)	1
Cholesteryl esters to total lipids ratio in very large HDL	%	0.00238 (0.98356)	0.02581 (0.0018)	< 0.001	-0.00094 (1.03159)	0.00972 (0.00817)	1
Free cholesterol to total lipids ratio in very large HDL	%	0.0154 (0.991)	-0.0325 (0.00156)	< 0.001	0.02596 (0.99559)	-0.02593 (0.00689)	0.043
Triglycerides to total lipids ratio in very large HDL	%	0.03273 (0.98071)	-0.0615 (0.00162)	< 0.001	0.0292 (0.97426)	-0.04947 (0.0072)	< 0.001
Phospholipids to total lipids ratio in large HDL	%	0.00153 (0.96713)	-0.05992 (0.0016)	< 0.001	0.00259 (0.86399)	-0.05417 (0.0061)	< 0.001
Cholesterol to total lipids ratio in large HDL	%	-0.00215 (0.94548)	0.05419 (0.00159)	< 0.001	-0.00202 (0.97752)	0.04274 (0.00722)	< 0.001
Cholesteryl esters to total lipids ratio in large HDL	%	-0.00124 (0.95247)	0.06039 (0.00162)	< 0.001	0.00022 (0.96792)	0.04428 (0.00731)	< 0.001
Free cholesterol to total lipids ratio in large HDL	%	-0.00511 (0.9684)	0.01768 (0.00163)	< 0.001	-0.00565 (0.97618)	0.02139 (0.00728)	0.834
Triglycerides to total lipids ratio in large HDL	%	0.02822 (0.97839)	-0.05229 (0.00165)	< 0.001	0.02773 (0.97745)	-0.03542 (0.00736)	< 0.001
Phospholipids to total lipids ratio in medium HDL	%	0.0246 (0.99779)	-0.05677 (0.00169)	< 0.001	0.03381 (0.97432)	-0.03352 (0.00712)	< 0.001
Cholesterol to total lipids ratio in medium HDL	%	-0.02578 (0.97861)	0.05719 (0.00168)	< 0.001	-0.03319 (0.97293)	0.03443 (0.0073)	< 0.001
Cholesteryl esters to total lipids ratio in medium HDL	%	-0.02375 (0.9784)	0.05836 (0.00172)	< 0.001	-0.02929 (0.97625)	0.03531 (0.00757)	< 0.001
Free cholesterol to total lipids ratio in medium HDL	%	-0.02119 (0.97879)	0.02795 (0.00154)	< 0.001	-0.03223 (0.98503)	0.01645 (0.00659)	1
Triglycerides to total lipids ratio in medium HDL	%	0.03855 (0.96507)	-0.05224 (0.00166)	< 0.001	0.04317 (0.96487)	-0.03275 (0.0073)	0.002
Phospholipids to total lipids ratio in small HDL	%	-0.00873 (0.98455)	-0.03106 (0.00174)	< 0.001	-0.01947 (0.99938)	-0.0301 (0.00771)	0.024
Cholesterol to total lipids ratio in small HDL	%	-0.01451 (0.97639)	0.05834 (0.00176)	< 0.001	-0.01187 (0.98307)	0.04123 (0.00772)	< 0.001
Cholesteryl esters to total lipids ratio in small HDL	%	-0.00706 (0.97818)	0.04732 (0.00178)	< 0.001	-0.00132 (0.98344)	0.02997 (0.00774)	0.027
Free cholesterol to total lipids ratio in small HDL	%	-0.02393 (0.96932)	0.03872 (0.00163)	< 0.001	-0.03144 (0.96814)	0.03642 (0.00672)	< 0.001
Triglycerides to total lipids ratio in small HDL	%	0.03863 (0.96513)	-0.04994 (0.00164)	< 0.001	0.04373 (0.96482)	-0.02864 (0.00716)	0.016
Total lipids							
Total lipids in lipoprotein particles	mmol/L	0.02064 (0.98395)	-0.03778 (0.00168)	< 0.001	0.01746 (0.99672)	-0.0315 (0.00739)	0.005
Total lipids in VLDL	mmol/L	0.05461 (0.95832)	-0.05178 (0.00166)	< 0.001	0.05819 (0.95982)	-0.03528 (0.00745)	< 0.001
Total lipids in LDL	mmol/L	0.01735 (0.98716)	-0.01729 (0.00168)	< 0.001	0.02066 (0.99904)	-0.01453 (0.00745)	1
Total lipids in HDL	mmol/L	-0.02442 (0.97697)	-0.00471 (0.00159)	0.749	-0.03704 (0.98578)	-0.01823 (0.00683)	1
Triglycerides							
Total triglycerides	mmol/L	0.04633 (0.96977)	-0.06248 (0.00168)	< 0.001	0.0467 (0.96954)	-0.04543 (0.00752)	< 0.001
Triglycerides in VLDL	mmol/L	0.05144 (0.96679)	-0.05834 (0.00166)	< 0.001	0.05193 (0.96614)	-0.04084 (0.00743)	< 0.001
Triglycerides in LDL	mmol/L	0.00848 (0.98921)	-0.0732 (0.00177)	< 0.001	0.00768 (0.99726)	-0.05917 (0.00789)	< 0.001
Triglycerides in HDL	mmol/L	0.01968 (0.97893)	-0.0636 (0.00176)	< 0.001	0.01665 (0.98151)	-0.05161 (0.00773)	< 0.001

Supplementary Table 9. Associations of the 113 metabolites comprised the metabolomic signature with healthy lifestyle components, healthy lifestyle scores, and the risk of schizophrenia. (Figure 2 source data)

			Healthy diet		Regular physical activity		No smoking		Moderate alcohol consumption		Frequent social contact		Adequate sleep duration		Less sedentary behavior		Healthy lifestyle score		Schizophrenia risk	
**Metabolic biomarkers**	**Unit**	**Coefficients**	**Beta(SE) †**	***P* value ***	**Beta(SE) †**	***P* value ***	**Beta(SE) †**	***P* value ***	**Beta(SE) †**	***P* value ***	**Beta(SE) †**	***P* value ***	**Beta(SE) †**	***P* value ***	**Beta(SE) †**	***P* value ***	**Beta(SE) †**	***P* value ***	**Beta(SE) †**	***P* value ***
Amino acids																				
Alanine	mmol/L	-0.00938737	-0.00709 (0.00483)	1	-0.01014 (0.00605)	1	0.05424 (0.00482)	< 0.001	0.0145 (0.00478)	0.272	-0.00697 (0.00696)	1	-0.00428 (0.00512)	1	-0.00925 (0.00553)	1	0.00577 (0.0018)	0.154	-0.06558 (0.05184)	1
Glutamine	mmol/L	-0.02413742	0.05824 (0.00484)	< 0.001	0.0528 (0.00606)	< 0.001	0.03625 (0.00483)	< 0.001	0.04862 (0.00479)	< 0.001	0.00861 (0.00697)	1	0.00085 (0.00513)	1	0.03886 (0.00554)	< 0.001	0.02956 (0.0018)	< 0.001	0.00606 (0.0509)	1
Glycine	mmol/L	0.02453992	0.03937 (0.00451)	< 0.001	0.04422 (0.00565)	< 0.001	0.03446 (0.0045)	< 0.001	0.04623 (0.00446)	< 0.001	-0.00373 (0.0065)	1	-0.01001 (0.00478)	1	0.04114 (0.00516)	< 0.001	0.02366 (0.00168)	< 0.001	0.06656 (0.05489)	1
Histidine	mmol/L	0.00074172	0.00881 (0.00485)	1	0.02735 (0.00607)	< 0.001	0.01796 (0.00484)	0.023	0.01774 (0.00479)	0.024	0.02524 (0.00698)	0.034	0.01243 (0.00514)	1	0.03028 (0.00554)	< 0.001	0.01513 (0.00181)	< 0.001	-0.04674 (0.05374)	1
Total concentration of branched-chain amino acids (leucine + isoleucine + valine)	mmol/L	-0.02870968	-0.0374 (0.00469)	< 0.001	-0.03318 (0.00587)	< 0.001	0.02042 (0.00468)	0.001	0.01911 (0.00464)	0.004	0.03976 (0.00676)	< 0.001	0.00461 (0.00498)	1	-0.008 (0.00537)	1	-0.00019 (0.00175)	1	-0.03754 (0.05347)	1
Isoleucine	mmol/L	-0.01781396	-0.03669 (0.00482)	< 0.001	-0.04166 (0.00603)	< 0.001	0.02271 (0.00481)	< 0.001	0.02757 (0.00476)	< 0.001	0.02211 (0.00694)	0.162	-0.00265 (0.00511)	1	-0.00797 (0.00551)	1	-0.0014 (0.0018)	1	0.03368 (0.04999)	1
Valine	mmol/L	0.03448711	-0.01798 (0.00469)	0.014	-0.04258 (0.00586)	< 0.001	0.02311 (0.00468)	< 0.001	0.01719 (0.00464)	0.024	0.03578 (0.00675)	< 0.001	0.00773 (0.00497)	1	-0.01209 (0.00536)	1	0.00146 (0.00175)	1	-0.05439 (0.05368)	1
Phenylalanine	mmol/L	-0.04629388	-0.03702 (0.00475)	< 0.001	-0.04073 (0.00595)	< 0.001	0.00927 (0.00474)	1	-0.01572 (0.0047)	0.094	0.00417 (0.00684)	1	-0.00979 (0.00504)	1	0.00425 (0.00544)	1	-0.01018 (0.00177)	< 0.001	0.06177 (0.0492)	1
Tyrosine	mmol/L	0.10507331	-0.02089 (0.00485)	0.002	-0.04142 (0.00607)	< 0.001	-0.00922 (0.00484)	1	-0.07362 (0.0048)	< 0.001	0.0228 (0.00699)	0.125	-0.00099 (0.00515)	1	0.01287 (0.00555)	1	-0.01555 (0.00181)	< 0.001	0.08429 (0.04933)	1
Apolipoproteins																				
Ratio of apolipoprotein B to apolipoprotein A1	ratio	-0.27700959	-0.0164 (0.00449)	0.029	-0.10906 (0.00561)	< 0.001	0.03122 (0.00448)	< 0.001	0.11382 (0.00443)	< 0.001	-0.07002 (0.00646)	< 0.001	-0.02264 (0.00476)	< 0.001	-0.0899 (0.00513)	< 0.001	-0.00847 (0.00167)	< 0.001	0.12502 (0.05425)	1
Cholesterol																				
Total cholesterol minus HDL-C	mmol/L	0.06437846	-0.03403 (0.0044)	< 0.001	-0.01032 (0.00551)	1	-0.01092 (0.00439)	1	-0.0174 (0.00436)	0.007	-0.0043 (0.00634)	1	-0.01576 (0.00467)	0.084	-0.04103 (0.00504)	< 0.001	-0.01624 (0.00164)	< 0.001	-0.02283 (0.05592)	1
Rem nt cholesterol (non-HDL, non-LDL -cholesterol)	mmol/L	0.0000104	-0.04066 (0.00436)	< 0.001	-0.02764 (0.00545)	< 0.001	-0.01284 (0.00435)	0.356	-0.0199 (0.00431)	< 0.001	-0.01463 (0.00627)	1	-0.0159 (0.00462)	0.065	-0.05073 (0.00498)	< 0.001	-0.02106 (0.00162)	< 0.001	0.01514 (0.05671)	1
Fatty acids																				
Total fatty acids	mmol/L	0.06349895	-0.08242 (0.00468)	< 0.001	-0.05655 (0.00587)	< 0.001	-0.09794 (0.00467)	< 0.001	-0.11766 (0.00463)	< 0.001	-0.014 (0.00675)	1	-0.03702 (0.00497)	< 0.001	-0.0845 (0.00536)	< 0.001	-0.06136 (0.00174)	< 0.001	0.05682 (0.05189)	1
Omega-6 fatty acids	mmol/L	0.02385051	-0.04557 (0.00456)	< 0.001	0.0175 (0.00571)	0.247	-0.02277 (0.00455)	< 0.001	-0.04602 (0.00451)	< 0.001	0.01185 (0.00657)	1	-0.0142 (0.00484)	0.378	-0.03317 (0.00522)	< 0.001	-0.01898 (0.0017)	< 0.001	-0.05829 (0.05414)	1
Linoleic acid	mmol/L	0.22450021	-0.03993 (0.00452)	< 0.001	0.02357 (0.00566)	0.003	0.0041 (0.00451)	1	0.00166 (0.00447)	1	0.00023 (0.00651)	1	-0.00738 (0.00479)	1	-0.01418 (0.00517)	0.687	-0.00506 (0.00168)	0.304	-0.05274 (0.05445)	1
Docosahexaenoic acid	mmol/L	0.474989	0.33512 (0.0046)	< 0.001	0.10393 (0.00584)	< 0.001	0.02575 (0.00466)	< 0.001	-0.03623 (0.00462)	< 0.001	0.06854 (0.00672)	< 0.001	0.05001 (0.00495)	< 0.001	0.07146 (0.00534)	< 0.001	0.07263 (0.00173)	< 0.001	-0.09803 (0.05541)	1
Ratio of omega-3 fatty acids to total fatty acids	%	0.30662044	0.37323 (0.00458)	< 0.001	0.09236 (0.00583)	< 0.001	0.06139 (0.00465)	< 0.001	0.01927 (0.00461)	0.003	0.08039 (0.00671)	< 0.001	0.06577 (0.00494)	< 0.001	0.07592 (0.00533)	< 0.001	0.09298 (0.00172)	< 0.001	-0.1244 (0.05343)	1
Ratio of omega-6 fatty acids to total fatty acids	%	-0.15867545	0.09253 (0.00454)	< 0.001	0.13788 (0.00568)	< 0.001	0.15803 (0.00452)	< 0.001	0.16102 (0.00448)	< 0.001	0.04581 (0.00655)	< 0.001	0.05112 (0.00482)	< 0.001	0.11616 (0.0052)	< 0.001	0.09177 (0.00168)	< 0.001	-0.1855 (0.04885)	0.017
Ratio of saturated fatty acids to total fatty acids	%	-0.03546887	-0.19778 (0.00476)	< 0.001	-0.08817 (0.00599)	< 0.001	-0.16174 (0.00476)	< 0.001	-0.21141 (0.00471)	< 0.001	-0.00486 (0.0069)	1	-0.03711 (0.00508)	< 0.001	-0.05345 (0.00548)	< 0.001	-0.09853 (0.00177)	< 0.001	0.13246 (0.05012)	0.929
Ratio of linoleic acid to total fatty acids	%	-0.12158502	0.06526 (0.00438)	< 0.001	0.12914 (0.00548)	< 0.001	0.1633 (0.00435)	< 0.001	0.1903 (0.00431)	< 0.001	0.02388 (0.00631)	0.017	0.04708 (0.00465)	< 0.001	0.11316 (0.00501)	< 0.001	0.08981 (0.00162)	< 0.001	-0.18284 (0.05242)	0.055
Ratio of docosahexaenoic acid to total fatty acids	%	0.19472377	0.38934 (0.0045)	< 0.001	0.14472 (0.00574)	< 0.001	0.08466 (0.00458)	< 0.001	0.0357 (0.00455)	< 0.001	0.0882 (0.00662)	< 0.001	0.07747 (0.00487)	< 0.001	0.12478 (0.00525)	< 0.001	0.11261 (0.00169)	< 0.001	-0.15842 (0.05377)	0.363
Ratio of polyunsaturated fatty acids to monounsaturated fatty acids	ratio	-0.08936681	0.23201 (0.00434)	< 0.001	0.1732 (0.00546)	< 0.001	0.15364 (0.00435)	< 0.001	0.10673 (0.00432)	< 0.001	0.08171 (0.00629)	< 0.001	0.07286 (0.00463)	< 0.001	0.15872 (0.00499)	< 0.001	0.11559 (0.00161)	< 0.001	-0.23203 (0.05445)	0.002
Fluid balance																				
Creatinine	mmol/L	0.08420285	-0.0877 (0.00396)	< 0.001	-0.01988 (0.00496)	0.007	0.02886 (0.00395)	< 0.001	0.03132 (0.00392)	< 0.001	-0.00069 (0.00571)	1	-0.01138 (0.0042)	0.764	-0.05625 (0.00453)	< 0.001	-0.01291 (0.00148)	< 0.001	0.06551 (0.04061)	1
Albumin	g/l	-0.0468249	0.07443 (0.00481)	< 0.001	0.09779 (0.00601)	< 0.001	0.03338 (0.0048)	< 0.001	-0.0079 (0.00476)	1	0.0424 (0.00692)	< 0.001	0.03603 (0.0051)	< 0.001	0.05404 (0.0055)	< 0.001	0.0356 (0.00179)	< 0.001	-0.17062 (0.04906)	0.057
Glycolysis related metabolites																				
Glucose-lactate	mmol/L	0.10973823	-0.01865 (0.0044)	0.003	-0.04577 (0.00551)	< 0.001	-0.00241 (0.00439)	1	-0.05077 (0.00435)	< 0.001	-0.03653 (0.00633)	< 0.001	-0.02808 (0.00467)	< 0.001	-0.05122 (0.00503)	< 0.001	-0.02556 (0.00164)	< 0.001	0.08018 (0.04979)	1
Glucose	mmol/L	0.05286267	0.0132 (0.00452)	0.398	-0.01749 (0.00566)	0.227	0.01529 (0.00451)	0.08	-0.05373 (0.00447)	< 0.001	0.00559 (0.00651)	1	-0.01877 (0.0048)	0.01	-0.03172 (0.00518)	< 0.001	-0.01053 (0.00169)	< 0.001	0.08186 (0.05038)	1
Lactate	mmol/L	-0.09705496	-0.06631 (0.00493)	< 0.001	-0.05621 (0.00618)	< 0.001	-0.03504 (0.00493)	< 0.001	0.00257 (0.00488)	1	-0.0842 (0.00711)	< 0.001	-0.01671 (0.00523)	0.16	-0.04724 (0.00565)	< 0.001	-0.03146 (0.00184)	< 0.001	-0.00984 (0.0503)	1
Pyruvate	mmol/L	-0.00388647	-0.04172 (0.00478)	< 0.001	-0.03711 (0.00598)	< 0.001	-0.03162 (0.00477)	< 0.001	-0.01518 (0.00473)	0.149	-0.05956 (0.00688)	< 0.001	-0.01561 (0.00507)	0.233	-0.02242 (0.00547)	0.005	-0.02397 (0.00178)	< 0.001	0.06872 (0.05023)	1
Citrate	mmol/L	0.00782488	0.00817 (0.0048)	1	0.0744 (0.006)	< 0.001	0.1377 (0.00478)	< 0.001	0.06107 (0.00475)	< 0.001	-0.05148 (0.00691)	< 0.001	-0.00029 (0.00509)	1	-0.00642 (0.00549)	1	0.03147 (0.00179)	< 0.001	0.02555 (0.05108)	1
Inflammation																				
Glycoprotein acetyls	mmol/L	-0.16163195	-0.10601 (0.00461)	< 0.001	-0.18407 (0.00576)	< 0.001	-0.10429 (0.0046)	< 0.001	-0.05906 (0.00457)	< 0.001	-0.0919 (0.00665)	< 0.001	-0.07331 (0.00489)	< 0.001	-0.1857 (0.00526)	< 0.001	-0.08897 (0.00171)	< 0.001	0.21781 (0.05164)	0.003
Ketone bodies																				
3-Hydroxybutyrate	mmol/L	-0.022902	-0.01539 (0.00489)	0.186	-0.00137 (0.00612)	1	-0.07017 (0.00488)	< 0.001	-0.08953 (0.00483)	< 0.001	-0.05202 (0.00704)	< 0.001	-0.01737 (0.00519)	0.092	-0.05666 (0.00559)	< 0.001	-0.03643 (0.00182)	< 0.001	0.01959 (0.04986)	1
Acetate	mmol/L	0.04073117	0.02011 (0.00203)	< 0.001	0.0174 (0.00254)	< 0.001	0.00627 (0.00203)	0.224	0.0031 (0.00201)	1	0.00802 (0.00293)	0.691	0.00576 (0.00216)	0.854	0.01319 (0.00233)	< 0.001	0.00831 (0.00076)	< 0.001	0.08011 (0.07942)	1
Acetone	mmol/L	-0.02130678	-0.00064 (0.00474)	1	0.03261 (0.00593)	< 0.001	-0.07032 (0.00473)	< 0.001	-0.07906 (0.00469)	< 0.001	-0.03505 (0.00683)	< 0.001	-0.01126 (0.00503)	1	0.00469 (0.00542)	1	-0.02149 (0.00177)	< 0.001	0.02344 (0.0492)	1
Lipoprotein particle concentrations																				
Total concentration of lipoprotein particles	mmol/L	-0.147942	-0.03793 (0.00451)	< 0.001	0.10379 (0.00564)	< 0.001	-0.07798 (0.00449)	< 0.001	-0.18909 (0.00444)	< 0.001	0.06919 (0.00649)	< 0.001	-0.00743 (0.00478)	1	0.03003 (0.00516)	< 0.001	-0.0269 (0.00168)	< 0.001	-0.17531 (0.05685)	0.231
Concentration of HDL particles	mmol/L	-0.00038094	-0.03491 (0.00451)	< 0.001	0.11453 (0.00564)	< 0.001	-0.07996 (0.00449)	< 0.001	-0.19852 (0.00443)	< 0.001	0.07631 (0.00649)	< 0.001	-0.00462 (0.00478)	1	0.04116 (0.00516)	< 0.001	-0.02514 (0.00168)	< 0.001	-0.18969 (0.05723)	0.104
Lipoprotein particle sizes																				
Average diameter for VLDL particles	nm	0.06846202	-0.11569 (0.0042)	< 0.001	-0.11009 (0.00527)	< 0.001	-0.04575 (0.0042)	< 0.001	0.001 (0.00417)	1	-0.03252 (0.00606)	< 0.001	-0.0378 (0.00447)	< 0.001	-0.10012 (0.00481)	< 0.001	-0.04959 (0.00157)	< 0.001	0.03694 (0.05816)	1
Average diameter for LDL particles	nm	-0.00056434	0.07347 (0.00467)	< 0.001	0.06418 (0.00584)	< 0.001	0.00921 (0.00466)	1	0.00132 (0.00462)	1	0.02237 (0.00672)	0.1	0.01724 (0.00495)	0.056	0.04057 (0.00534)	< 0.001	0.02532 (0.00174)	< 0.001	-0.10258 (0.05138)	1
Average diameter for HDL particles	nm	-0.03276584	0.0945 (0.00406)	< 0.001	0.11834 (0.00508)	< 0.001	0.0212 (0.00406)	< 0.001	-0.04264 (0.00402)	< 0.001	0.05314 (0.00585)	< 0.001	0.04534 (0.00431)	< 0.001	0.1157 (0.00464)	< 0.001	0.042 (0.00151)	< 0.001	-0.02148 (0.06213)	1
Lipoprotein subclasses																				
Cholesteryl esters in chylomicrons and extremely large VLDL	mmol/L	-0.00739703	-0.12031 (0.00454)	< 0.001	-0.12622 (0.00569)	< 0.001	-0.05993 (0.00454)	< 0.001	-0.04683 (0.0045)	< 0.001	-0.0504 (0.00656)	< 0.001	-0.04863 (0.00483)	< 0.001	-0.12733 (0.0052)	< 0.001	-0.06586 (0.00169)	< 0.001	0.15317 (0.05003)	0.249
Free cholesterol in chylomicrons and extremely large VLDL	mmol/L	0.24435793	-0.11432 (0.00457)	< 0.001	-0.12597 (0.00572)	< 0.001	-0.07457 (0.00456)	< 0.001	-0.06167 (0.00452)	< 0.001	-0.05099 (0.00658)	< 0.001	-0.04529 (0.00485)	< 0.001	-0.11509 (0.00523)	< 0.001	-0.06749 (0.0017)	< 0.001	0.14343 (0.04894)	0.382
Triglycerides in chylomicrons and extremely large VLDL	mmol/L	-0.01002038	-0.12546 (0.00458)	< 0.001	-0.13198 (0.00574)	< 0.001	-0.07752 (0.00458)	< 0.001	-0.06019 (0.00454)	< 0.001	-0.04759 (0.00661)	< 0.001	-0.04448 (0.00487)	< 0.001	-0.10403 (0.00525)	< 0.001	-0.06827 (0.0017)	< 0.001	0.14296 (0.04891)	0.392
Total lipids in very large VLDL	mmol/L	-0.0652873	-0.11264 (0.00447)	< 0.001	-0.12642 (0.00559)	< 0.001	-0.06809 (0.00446)	< 0.001	-0.03162 (0.00443)	< 0.001	-0.04752 (0.00644)	< 0.001	-0.04312 (0.00474)	< 0.001	-0.11292 (0.00511)	< 0.001	-0.06139 (0.00166)	< 0.001	0.12823 (0.05261)	1
Phospholipids in very large VLDL	mmol/L	-0.02840217	-0.1075 (0.0045)	< 0.001	-0.12809 (0.00564)	< 0.001	-0.0639 (0.0045)	< 0.001	-0.02834 (0.00446)	< 0.001	-0.05272 (0.00649)	< 0.001	-0.04352 (0.00478)	< 0.001	-0.11603 (0.00515)	< 0.001	-0.0605 (0.00168)	< 0.001	0.13738 (0.05122)	0.826
Triglycerides in very large VLDL	mmol/L	0.05361357	-0.11495 (0.00449)	< 0.001	-0.1253 (0.00562)	< 0.001	-0.07833 (0.00448)	< 0.001	-0.04421 (0.00445)	< 0.001	-0.04412 (0.00648)	< 0.001	-0.04342 (0.00477)	< 0.001	-0.10615 (0.00514)	< 0.001	-0.06392 (0.00167)	< 0.001	0.12721 (0.05131)	1
Total lipids in large VLDL	mmol/L	-0.01468282	-0.09896 (0.00447)	< 0.001	-0.11288 (0.0056)	< 0.001	-0.05549 (0.00446)	< 0.001	-0.01033 (0.00443)	1	-0.04158 (0.00645)	< 0.001	-0.03777 (0.00475)	< 0.001	-0.10618 (0.00511)	< 0.001	-0.05173 (0.00166)	< 0.001	0.09376 (0.05391)	1
Cholesteryl esters in large VLDL	mmol/L	-0.17788313	-0.06771 (0.00453)	< 0.001	-0.09435 (0.00566)	< 0.001	-0.02316 (0.00452)	< 0.001	0.01971 (0.00448)	0.001	-0.04635 (0.00652)	< 0.001	-0.03037 (0.0048)	< 0.001	-0.10612 (0.00517)	< 0.001	-0.03636 (0.00169)	< 0.001	0.08664 (0.05397)	1
Triglycerides in large VLDL	mmol/L	0.03703655	-0.09908 (0.00452)	< 0.001	-0.10768 (0.00565)	< 0.001	-0.06173 (0.00451)	< 0.001	-0.01858 (0.00447)	0.004	-0.03385 (0.00651)	< 0.001	-0.03675 (0.00479)	< 0.001	-0.0958 (0.00517)	< 0.001	-0.05158 (0.00168)	< 0.001	0.08126 (0.05271)	1
Cholesterol in medium VLDL	mmol/L	-0.22420044	-0.0109 (0.00442)	1	-0.01963 (0.00553)	0.044	0.02295 (0.00441)	< 0.001	0.03647 (0.00437)	< 0.001	-0.01809 (0.00636)	0.504	-0.00929 (0.00468)	1	-0.04809 (0.00505)	< 0.001	-0.00234 (0.00165)	1	-0.00776 (0.05674)	1
Cholesteryl esters in medium VLDL	mmol/L	0.07371362	0.00804 (0.00436)	1	0.00475 (0.00546)	1	0.04135 (0.00435)	< 0.001	0.04616 (0.00432)	< 0.001	-0.01006 (0.00628)	1	-0.00085 (0.00463)	1	-0.02703 (0.00499)	< 0.001	0.01022 (0.00163)	< 0.001	-0.04034 (0.0578)	1
Total lipids in small VLDL	mmol/L	0.05888054	-0.0538 (0.00459)	< 0.001	-0.08991 (0.00575)	< 0.001	-0.0429 (0.00458)	< 0.001	-0.00582 (0.00454)	1	-0.04481 (0.00661)	< 0.001	-0.03128 (0.00487)	< 0.001	-0.10079 (0.00525)	< 0.001	-0.03985 (0.00171)	< 0.001	0.1029 (0.05297)	1
Triglycerides in small VLDL	mmol/L	-0.18090251	-0.06322 (0.0046)	< 0.001	-0.10197 (0.00576)	< 0.001	-0.07063 (0.00459)	< 0.001	-0.02977 (0.00455)	< 0.001	-0.04233 (0.00663)	< 0.001	-0.03346 (0.00488)	< 0.001	-0.09846 (0.00526)	< 0.001	-0.04936 (0.00171)	< 0.001	0.1273 (0.05108)	1
Free cholesterol in IDL	mmol/L	0.01722062	0.01244 (0.00425)	0.385	0.03299 (0.00532)	< 0.001	0.01417 (0.00424)	0.093	-0.01849 (0.0042)	0.001	0.01027 (0.00612)	1	0.00221 (0.0045)	1	-0.00167 (0.00486)	1	0.00479 (0.00158)	0.28	-0.04811 (0.05903)	1
Triglycerides in large LDL	mmol/L	0.03058474	-0.07182 (0.00477)	< 0.001	-0.10683 (0.00597)	< 0.001	-0.12532 (0.00476)	< 0.001	-0.11065 (0.00472)	< 0.001	-0.06138 (0.00688)	< 0.001	-0.0471 (0.00506)	< 0.001	-0.1021 (0.00546)	< 0.001	-0.07347 (0.00177)	< 0.001	0.18014 (0.04594)	0.01
Phospholipids in medium LDL	mmol/L	0.02543848	-0.05139 (0.00462)	< 0.001	-0.03248 (0.00578)	< 0.001	-0.02741 (0.00461)	< 0.001	-0.01457 (0.00457)	0.162	-0.01539 (0.00665)	1	-0.02998 (0.0049)	< 0.001	-0.06438 (0.00529)	< 0.001	-0.0275 (0.00172)	< 0.001	-0.0125 (0.05413)	1
Free cholesterol in medium LDL	mmol/L	0.05025915	-0.01601 (0.00457)	0.052	0.01279 (0.00572)	1	0.00559 (0.00456)	1	0.00679 (0.00452)	1	0.00532 (0.00658)	1	-0.01403 (0.00485)	0.431	-0.02802 (0.00523)	< 0.001	-0.0037 (0.0017)	1	-0.06922 (0.05505)	1
Triglycerides in medium LDL	mmol/L	0.02228101	-0.08106 (0.00474)	< 0.001	-0.11605 (0.00593)	< 0.001	-0.11291 (0.00472)	< 0.001	-0.08773 (0.00469)	< 0.001	-0.06233 (0.00683)	< 0.001	-0.04962 (0.00503)	< 0.001	-0.11024 (0.00542)	< 0.001	-0.07182 (0.00176)	< 0.001	0.17652 (0.0454)	0.011
Concentration of small LDL particles	mmol/L	0.02829835	-0.03623 (0.00454)	< 0.001	-0.05743 (0.00569)	< 0.001	-0.00573 (0.00453)	1	0.02353 (0.0045)	< 0.001	-0.03474 (0.00654)	< 0.001	-0.02341 (0.00482)	< 0.001	-0.06979 (0.0052)	< 0.001	-0.02023 (0.00169)	< 0.001	0.06714 (0.05393)	1
Phospholipids in very large HDL	mmol/L	0.0008266	0.10359 (0.00404)	< 0.001	0.08911 (0.00506)	< 0.001	0.0291 (0.00404)	< 0.001	-0.00817 (0.004)	1	0.02962 (0.00583)	< 0.001	0.04165 (0.00429)	< 0.001	0.10449 (0.00462)	< 0.001	0.04345 (0.00151)	< 0.001	0.01992 (0.06225)	1
Cholesteryl esters in very large HDL	mmol/L	0.03123849	0.10871 (0.00397)	< 0.001	0.10352 (0.00497)	< 0.001	0.03511 (0.00397)	< 0.001	-0.00371 (0.00393)	1	0.03721 (0.00573)	< 0.001	0.04037 (0.00422)	< 0.001	0.10705 (0.00454)	< 0.001	0.04753 (0.00148)	< 0.001	-0.02584 (0.06453)	1
Free cholesterol in very large HDL	mmol/L	0.11582895	0.09811 (0.00411)	< 0.001	0.0574 (0.00516)	< 0.001	0.04112 (0.00411)	< 0.001	0.03008 (0.00408)	< 0.001	0.0076 (0.00593)	1	0.0366 (0.00437)	< 0.001	0.08704 (0.00471)	< 0.001	0.04303 (0.00153)	< 0.001	0.07936 (0.05958)	1
Triglycerides in large HDL	mmol/L	-0.02460892	-0.03563 (0.00471)	< 0.001	0.00341 (0.0059)	1	-0.05747 (0.0047)	< 0.001	-0.10537 (0.00465)	< 0.001	-0.00227 (0.00678)	1	0.0039 (0.005)	1	-0.00014 (0.00539)	1	-0.02733 (0.00176)	< 0.001	0.11318 (0.04924)	1
Concentration of medium HDL particles	mmol/L	0.13266188	-0.01983 (0.00441)	< 0.001	0.12055 (0.00551)	< 0.001	-0.07855 (0.0044)	< 0.001	-0.20435 (0.00433)	< 0.001	0.07832 (0.00635)	< 0.001	0.00496 (0.00468)	1	0.0611 (0.00504)	< 0.001	-0.0197 (0.00164)	< 0.001	-0.17364 (0.05886)	0.359
Phospholipids in medium HDL	mmol/L	0.00549817	-0.04386 (0.00453)	< 0.001	0.09776 (0.00567)	< 0.001	-0.10378 (0.00452)	< 0.001	-0.22934 (0.00445)	< 0.001	0.07347 (0.00652)	< 0.001	-0.00236 (0.00481)	1	0.044 (0.00518)	< 0.001	-0.03518 (0.00169)	< 0.001	-0.14685 (0.05589)	0.972
Triglycerides in medium HDL	mmol/L	-0.06497412	-0.11263 (0.00473)	< 0.001	-0.06432 (0.00593)	< 0.001	-0.10357 (0.00473)	< 0.001	-0.13878 (0.00468)	< 0.001	-0.02037 (0.00683)	0.322	-0.02894 (0.00503)	< 0.001	-0.07259 (0.00542)	< 0.001	-0.0682 (0.00176)	< 0.001	0.09762 (0.04967)	1
Concentration of small HDL particles	mmol/L	0.01579286	-0.10378 (0.00482)	< 0.001	0.04804 (0.00604)	< 0.001	-0.09807 (0.00481)	< 0.001	-0.18363 (0.00475)	< 0.001	0.05072 (0.00695)	< 0.001	-0.03566 (0.00512)	< 0.001	-0.03615 (0.00552)	< 0.001	-0.05479 (0.00179)	< 0.001	-0.17084 (0.05285)	0.139
Cholesteryl esters in small HDL	mmol/L	0.000063	-0.09055 (0.00483)	< 0.001	0.06884 (0.00605)	< 0.001	-0.08153 (0.00482)	< 0.001	-0.17053 (0.00477)	< 0.001	0.06294 (0.00696)	< 0.001	-0.03048 (0.00513)	< 0.001	-0.02034 (0.00553)	0.027	-0.0438 (0.0018)	< 0.001	-0.21405 (0.05194)	0.004
Other lipids																				
Ratio of triglycerides to phosphoglycerides	ratio	-0.23344092	-0.09862 (0.00427)	< 0.001	-0.15156 (0.00534)	< 0.001	-0.05182 (0.00427)	< 0.001	0.02068 (0.00423)	< 0.001	-0.06532 (0.00616)	< 0.001	-0.0385 (0.00453)	< 0.001	-0.11398 (0.00489)	< 0.001	-0.05271 (0.00159)	< 0.001	0.17301 (0.05615)	0.233
Total cholines	mmol/L	-0.11400209	-0.03186 (0.00444)	< 0.001	0.0582 (0.00556)	< 0.001	-0.07504 (0.00443)	< 0.001	-0.15409 (0.00438)	< 0.001	0.04019 (0.00639)	< 0.001	-0.00796 (0.00471)	1	0.00703 (0.00508)	1	-0.02917 (0.00165)	< 0.001	-0.0666 (0.05544)	1
Phosphatidylcholines	mmol/L	0.197826	-0.02956 (0.00445)	< 0.001	0.06691 (0.00557)	< 0.001	-0.08307 (0.00444)	< 0.001	-0.16175 (0.00439)	< 0.001	0.04708 (0.00641)	< 0.001	-0.0028 (0.00472)	1	0.02206 (0.00509)	0.002	-0.02759 (0.00166)	< 0.001	-0.07728 (0.05531)	1
Sphingomyelins	mmol/L	0.0263485	-0.00227 (0.00431)	1	0.0414 (0.0054)	< 0.001	-0.0474 (0.0043)	< 0.001	-0.10362 (0.00426)	< 0.001	0.02447 (0.00621)	0.009	-0.00919 (0.00457)	1	-0.00644 (0.00493)	1	-0.01816 (0.00161)	< 0.001	-0.03929 (0.05781)	1
Phospholipids																				
Phospholipids in VLDL	mmol/L	0.01920709	-0.08339 (0.00453)	< 0.001	-0.10707 (0.00567)	< 0.001	-0.04963 (0.00452)	< 0.001	-0.01702 (0.00448)	0.017	-0.04928 (0.00652)	< 0.001	-0.03642 (0.0048)	< 0.001	-0.1084 (0.00518)	< 0.001	-0.04976 (0.00169)	< 0.001	0.11597 (0.05335)	1
Relative lipoprotein lipid concentrations																				
Phospholipids to total lipids ratio in chylomicrons and extremely large VLDL	%	0.01583807	-0.03622 (0.00471)	< 0.001	-0.04115 (0.0059)	< 0.001	-0.01722 (0.0047)	0.028	0.01123 (0.00466)	1	-0.03232 (0.00679)	< 0.001	-0.00365 (0.005)	1	-0.05327 (0.00539)	< 0.001	-0.01777 (0.00176)	< 0.001	0.07564 (0.06027)	1
Cholesterol to total lipids ratio in chylomicrons and extremely large VLDL	%	-0.07323796	0.0572 (0.00491)	< 0.001	0.07002 (0.00614)	< 0.001	0.03078 (0.0049)	< 0.001	0.02345 (0.00486)	< 0.001	0.01384 (0.00707)	1	0.01443 (0.00521)	0.629	0.02353 (0.00562)	0.003	0.02701 (0.00183)	< 0.001	-0.04175 (0.05102)	1
Free cholesterol to total lipids ratio in chylomicrons and extremely large VLDL	%	0.00806868	0.0919 (0.00487)	< 0.001	0.08889 (0.0061)	< 0.001	0.01861 (0.00487)	0.015	0.00655 (0.00482)	1	0.01865 (0.00702)	0.895	0.02716 (0.00517)	< 0.001	0.05181 (0.00558)	< 0.001	0.03431 (0.00182)	< 0.001	-0.05039 (0.05077)	1
Triglycerides to total lipids ratio in chylomicrons and extremely large VLDL	%	0.10876325	-0.03655 (0.00503)	< 0.001	-0.04799 (0.00629)	< 0.001	0.00066 (0.00502)	1	0.0055 (0.00497)	1	-0.0027 (0.00724)	1	-0.00619 (0.00533)	1	-0.0217 (0.00575)	0.018	-0.01172 (0.00187)	< 0.001	-0.00128 (0.04765)	1
Phospholipids to total lipids ratio in very large VLDL	%	0.00515977	-0.03635 (0.0045)	< 0.001	-0.04871 (0.00563)	< 0.001	0.01646 (0.00449)	0.028	0.04412 (0.00445)	< 0.001	-0.03258 (0.00647)	< 0.001	-0.0015 (0.00477)	1	-0.04795 (0.00514)	< 0.001	-0.00827 (0.00168)	< 0.001	0.00317 (0.05467)	1
Cholesteryl esters to total lipids ratio in very large VLDL	%	-0.0189965	0.09884 (0.00417)	< 0.001	0.0901 (0.00522)	< 0.001	0.08174 (0.00416)	< 0.001	0.05864 (0.00413)	< 0.001	0.02429 (0.00601)	0.006	0.0235 (0.00443)	< 0.001	0.05267 (0.00478)	< 0.001	0.05161 (0.00155)	< 0.001	-0.13385 (0.05924)	1
Triglycerides to total lipids ratio in very large VLDL	%	0.01600851	-0.07232 (0.00355)	< 0.001	-0.04129 (0.00444)	< 0.001	-0.05171 (0.00354)	< 0.001	-0.03643 (0.00351)	< 0.001	0.00398 (0.00511)	1	-0.01402 (0.00377)	0.022	-0.02257 (0.00406)	< 0.001	-0.02998 (0.00132)	< 0.001	0.08435 (0.07662)	1
Phospholipids to total lipids ratio in large VLDL	%	-0.03470017	-0.07278 (0.00364)	< 0.001	-0.06093 (0.00456)	< 0.001	-0.01716 (0.00364)	< 0.001	0.01731 (0.0036)	< 0.001	-0.03192 (0.00525)	< 0.001	-0.00928 (0.00386)	1	-0.06175 (0.00417)	< 0.001	-0.02531 (0.00136)	< 0.001	0.03245 (0.07208)	1
Free cholesterol to total lipids ratio in large VLDL	%	-0.01266285	-0.01728 (0.00433)	0.007	-0.05272 (0.00542)	< 0.001	0.00753 (0.00432)	1	0.02655 (0.00428)	< 0.001	-0.05256 (0.00623)	< 0.001	-0.01729 (0.00459)	0.019	-0.0736 (0.00495)	< 0.001	-0.01574 (0.00161)	< 0.001	0.06627 (0.0612)	1
Phospholipids to total lipids ratio in medium VLDL	%	0.00282138	0.04037 (0.00408)	< 0.001	0.03772 (0.00511)	< 0.001	0.04707 (0.00408)	< 0.001	0.02511 (0.00404)	< 0.001	-0.00438 (0.00588)	1	0.0104 (0.00433)	1	0.01858 (0.00467)	0.008	0.02206 (0.00152)	< 0.001	-0.0012 (0.05736)	1
Cholesteryl esters to total lipids ratio in medium VLDL	%	0.10319431	0.07533 (0.00418)	< 0.001	0.0856 (0.00524)	< 0.001	0.08083 (0.00418)	< 0.001	0.04566 (0.00414)	< 0.001	0.0282 (0.00603)	< 0.001	0.02619 (0.00444)	< 0.001	0.06125 (0.00479)	< 0.001	0.04747 (0.00156)	< 0.001	-0.07489 (0.05089)	1
Free cholesterol to total lipids ratio in medium VLDL	%	0.14489926	0.07869 (0.00418)	< 0.001	0.06427 (0.00524)	< 0.001	0.07194 (0.00417)	< 0.001	0.04277 (0.00414)	< 0.001	0.0112 (0.00603)	1	0.02044 (0.00444)	< 0.001	0.04022 (0.00479)	< 0.001	0.04031 (0.00156)	< 0.001	-0.02305 (0.05632)	1
Free cholesterol to total lipids ratio in small VLDL	%	0.00681397	0.05515 (0.00422)	< 0.001	0.08294 (0.00528)	< 0.001	0.07338 (0.00421)	< 0.001	0.03975 (0.00417)	< 0.001	0.02947 (0.00607)	< 0.001	0.01815 (0.00447)	0.006	0.05797 (0.00482)	< 0.001	0.0413 (0.00157)	< 0.001	-0.13718 (0.05456)	1
Triglycerides to total lipids ratio in small VLDL	%	0.09156276	-0.04485 (0.00437)	< 0.001	-0.05351 (0.00546)	< 0.001	-0.07112 (0.00435)	< 0.001	-0.04196 (0.00432)	< 0.001	-0.00291 (0.00629)	1	-0.00963 (0.00463)	1	-0.02877 (0.005)	< 0.001	-0.03132 (0.00163)	< 0.001	0.09987 (0.05782)	1
Phospholipids to total lipids ratio in very small VLDL	%	-0.07630893	-0.00771 (0.00449)	1	-0.14582 (0.0056)	< 0.001	-0.02759 (0.00448)	< 0.001	0.05898 (0.00444)	< 0.001	-0.09656 (0.00646)	< 0.001	-0.02653 (0.00476)	< 0.001	-0.10089 (0.00513)	< 0.001	-0.02996 (0.00167)	< 0.001	0.22795 (0.05495)	0.004
Cholesteryl esters to total lipids ratio in very small VLDL	%	0.01534104	0.04708 (0.00408)	< 0.001	0.11453 (0.0051)	< 0.001	0.08517 (0.00406)	< 0.001	0.02977 (0.00403)	< 0.001	0.05549 (0.00587)	< 0.001	0.03464 (0.00432)	< 0.001	0.08936 (0.00466)	< 0.001	0.05032 (0.00152)	< 0.001	-0.2041 (0.05259)	0.012
Free cholesterol to total lipids ratio in very small VLDL	%	0.06520831	0.06136 (0.0046)	< 0.001	0.03594 (0.00576)	< 0.001	0.09367 (0.00459)	< 0.001	0.09604 (0.00455)	< 0.001	0.00563 (0.00663)	1	0.0147 (0.00488)	0.292	0.00609 (0.00526)	1	0.04128 (0.00171)	< 0.001	-0.06347 (0.0501)	1
Phospholipids to total lipids ratio in IDL	%	0.01821094	0.07142 (0.00481)	< 0.001	-0.05249 (0.00602)	< 0.001	0.0375 (0.0048)	< 0.001	0.09976 (0.00475)	< 0.001	-0.05513 (0.00692)	< 0.001	0.01267 (0.0051)	1	-0.0317 (0.0055)	< 0.001	0.01918 (0.00179)	< 0.001	0.11187 (0.05215)	1
Free cholesterol to total lipids ratio in IDL	%	-0.18375276	0.1021 (0.0047)	< 0.001	0.07222 (0.00589)	< 0.001	0.10929 (0.00469)	< 0.001	0.08734 (0.00465)	< 0.001	0.02859 (0.00678)	0.003	0.02455 (0.00499)	< 0.001	0.03005 (0.00539)	< 0.001	0.05642 (0.00175)	< 0.001	-0.07706 (0.04822)	1
Phospholipids to total lipids ratio in large LDL	%	-0.0309856	-0.04008 (0.00487)	< 0.001	-0.01245 (0.0061)	1	0.0504 (0.00486)	< 0.001	0.02292 (0.00482)	< 0.001	-0.00037 (0.00702)	1	0.00164 (0.00517)	1	-0.01546 (0.00558)	0.627	0.00215 (0.00182)	1	-0.03647 (0.0507)	1
Cholesteryl esters to total lipids ratio in large LDL	%	-0.10219356	0.02228 (0.00489)	< 0.001	0.03384 (0.00612)	< 0.001	0.01826 (0.00488)	0.021	0.03367 (0.00484)	< 0.001	0.03032 (0.00704)	0.002	0.0055 (0.00518)	1	0.02143 (0.00559)	0.014	0.01842 (0.00182)	< 0.001	-0.01973 (0.01977)	1
Free cholesterol to total lipids ratio in large LDL	%	0.01904536	0.04902 (0.00436)	< 0.001	0.1086 (0.00545)	< 0.001	0.08427 (0.00435)	< 0.001	0.04755 (0.00431)	< 0.001	0.05 (0.00628)	< 0.001	0.03276 (0.00462)	< 0.001	0.08431 (0.00498)	< 0.001	0.05133 (0.00162)	< 0.001	-0.06843 (0.02873)	1
Triglycerides to total lipids ratio in large LDL	%	-0.0268792	-0.05451 (0.00442)	< 0.001	-0.12192 (0.00553)	< 0.001	-0.11826 (0.0044)	< 0.001	-0.0861 (0.00437)	< 0.001	-0.06633 (0.00637)	< 0.001	-0.03298 (0.00469)	< 0.001	-0.07918 (0.00506)	< 0.001	-0.06408 (0.00164)	< 0.001	0.22012 (0.04799)	<0.001
Phospholipids to total lipids ratio in medium LDL	%	-0.00456825	0.04262 (0.00477)	< 0.001	0.01507 (0.00597)	1	0.02195 (0.00476)	< 0.001	0.04545 (0.00472)	< 0.001	-0.00687 (0.00687)	1	-0.00285 (0.00506)	1	0.03851 (0.00546)	< 0.001	0.02006 (0.00178)	< 0.001	-0.0104 (0.05197)	1
Cholesterol to total lipids ratio in medium LDL	%	-0.03081566	0.01136 (0.00499)	1	0.0588 (0.00624)	< 0.001	0.06329 (0.00498)	< 0.001	0.04252 (0.00493)	< 0.001	0.04412 (0.00718)	< 0.001	0.01982 (0.00529)	0.02	0.02395 (0.00571)	0.003	0.02963 (0.00186)	< 0.001	-0.02287 (0.01366)	1
Triglycerides to total lipids ratio in medium LDL	%	-0.00026666	-0.03948 (0.00449)	< 0.001	-0.0999 (0.00562)	< 0.001	-0.10741 (0.00447)	< 0.001	-0.08275 (0.00444)	< 0.001	-0.05564 (0.00647)	< 0.001	-0.02528 (0.00476)	< 0.001	-0.05868 (0.00514)	< 0.001	-0.05419 (0.00167)	< 0.001	0.21317 (0.04776)	<0.001
Phospholipids to total lipids ratio in small LDL	%	-0.00762658	0.1126 (0.0047)	< 0.001	0.02044 (0.00589)	0.059	0.01062 (0.0047)	1	0.07214 (0.00465)	< 0.001	-0.01512 (0.00678)	1	0.02901 (0.00499)	< 0.001	0.08238 (0.00538)	< 0.001	0.04052 (0.00175)	< 0.001	0.04834 (0.0527)	1
Cholesteryl esters to total lipids ratio in small LDL	%	-0.03749522	-0.11468 (0.00489)	< 0.001	-0.03176 (0.00614)	< 0.001	-0.016 (0.00489)	0.121	-0.06623 (0.00485)	< 0.001	0.00691 (0.00706)	1	-0.02523 (0.0052)	< 0.001	-0.08808 (0.00561)	< 0.001	-0.04242 (0.00183)	< 0.001	-0.02781 (0.02589)	1
Free cholesterol to total lipids ratio in small LDL	%	-0.02011366	0.08125 (0.00464)	< 0.001	0.101 (0.00581)	< 0.001	0.08585 (0.00463)	< 0.001	0.08294 (0.00459)	< 0.001	0.05114 (0.00669)	< 0.001	0.03299 (0.00492)	< 0.001	0.08527 (0.00531)	< 0.001	0.06059 (0.00173)	< 0.001	-0.08293 (0.03665)	1
Phospholipids to total lipids ratio in very large HDL	%	-0.01248039	0.05 (0.00473)	< 0.001	0.02517 (0.00592)	0.002	0.01467 (0.00472)	0.211	0.01071 (0.00468)	1	-0.00129 (0.00681)	1	0.02223 (0.00501)	0.001	0.05174 (0.00541)	< 0.001	0.02091 (0.00176)	< 0.001	-0.05743 (0.0359)	1
Cholesterol to total lipids ratio in very large HDL	%	0.01237351	-0.03714 (0.00465)	< 0.001	0.00084 (0.00582)	1	0.02654 (0.00464)	< 0.001	0.04579 (0.0046)	< 0.001	0.01945 (0.00669)	0.413	-0.01984 (0.00493)	0.006	-0.03649 (0.00532)	< 0.001	0.0001 (0.00173)	1	-0.05836 (0.04315)	1
Cholesteryl esters to total lipids ratio in very large HDL	%	0.01603374	0.02549 (0.00483)	< 0.001	0.07817 (0.00605)	< 0.001	0.03954 (0.00482)	< 0.001	0.0174 (0.00478)	0.031	0.05357 (0.00696)	< 0.001	0.00163 (0.00513)	1	0.03333 (0.00553)	< 0.001	0.02581 (0.0018)	< 0.001	-0.06578 (0.02546)	1
Free cholesterol to total lipids ratio in very large HDL	%	-0.00233934	-0.08947 (0.00418)	< 0.001	-0.1085 (0.00523)	< 0.001	-0.0029 (0.00417)	1	0.0553 (0.00414)	< 0.001	-0.04407 (0.00602)	< 0.001	-0.03664 (0.00443)	< 0.001	-0.09787 (0.00478)	< 0.001	-0.0325 (0.00156)	< 0.001	0.09359 (0.05455)	1
Triglycerides to total lipids ratio in very large HDL	%	-0.0093877	-0.11424 (0.00435)	< 0.001	-0.11174 (0.00545)	< 0.001	-0.0692 (0.00435)	< 0.001	-0.04253 (0.00432)	< 0.001	-0.04008 (0.00628)	< 0.001	-0.04344 (0.00463)	< 0.001	-0.11242 (0.00498)	< 0.001	-0.0615 (0.00162)	< 0.001	0.14136 (0.05268)	0.824
Cholesteryl esters to total lipids ratio in large HDL	%	-0.05112521	0.08936 (0.00435)	< 0.001	0.12108 (0.00544)	< 0.001	0.08115 (0.00434)	< 0.001	0.05615 (0.00431)	< 0.001	0.05008 (0.00627)	< 0.001	0.03715 (0.00462)	< 0.001	0.09385 (0.00498)	< 0.001	0.06039 (0.00162)	< 0.001	-0.10239 (0.03606)	0.51
Triglycerides to total lipids ratio in large HDL	%	-0.12158141	-0.10478 (0.00443)	< 0.001	-0.12691 (0.00555)	< 0.001	-0.04717 (0.00443)	< 0.001	-0.00163 (0.00439)	1	-0.05707 (0.00639)	< 0.001	-0.0334 (0.00471)	< 0.001	-0.11005 (0.00507)	< 0.001	-0.05229 (0.00165)	< 0.001	0.16254 (0.05365)	0.276
Cholesterol to total lipids ratio in medium HDL	%	-0.03958127	0.08964 (0.0045)	< 0.001	0.15534 (0.00563)	< 0.001	0.06176 (0.0045)	< 0.001	0.01012 (0.00446)	1	0.07485 (0.00649)	< 0.001	0.03366 (0.00478)	< 0.001	0.11019 (0.00515)	< 0.001	0.05719 (0.00168)	< 0.001	-0.06966 (0.01765)	0.009
Triglycerides to total lipids ratio in medium HDL	%	0.00099714	-0.10139 (0.00446)	< 0.001	-0.13349 (0.00559)	< 0.001	-0.0515 (0.00446)	< 0.001	-0.00351 (0.00442)	1	-0.06187 (0.00644)	< 0.001	-0.02635 (0.00474)	< 0.001	-0.10549 (0.00511)	< 0.001	-0.05224 (0.00166)	< 0.001	0.19933 (0.05668)	0.049
Phospholipids to total lipids ratio in small HDL	%	-0.03228249	-0.05096 (0.00468)	< 0.001	0.01478 (0.00586)	1	-0.07855 (0.00467)	< 0.001	-0.14695 (0.00462)	< 0.001	0.02537 (0.00674)	0.019	0.0105 (0.00497)	1	0.03353 (0.00536)	< 0.001	-0.03106 (0.00174)	< 0.001	-0.01228 (0.05286)	1
Cholesterol to total lipids ratio in small HDL	%	0.0309348	0.08967 (0.00472)	< 0.001	0.09728 (0.00591)	< 0.001	0.09217 (0.00471)	< 0.001	0.09321 (0.00467)	< 0.001	0.04111 (0.0068)	< 0.001	0.01811 (0.00501)	0.034	0.05782 (0.00541)	< 0.001	0.05834 (0.00176)	< 0.001	-0.14361 (0.0476)	0.289
Total lipids																				
Total lipids in VLDL	mmol/L	0.02155125	-0.09391 (0.00446)	< 0.001	-0.11034 (0.00559)	< 0.001	-0.05401 (0.00446)	< 0.001	-0.01874 (0.00442)	0.003	-0.04386 (0.00643)	< 0.001	-0.03596 (0.00474)	< 0.001	-0.10673 (0.00511)	< 0.001	-0.05178 (0.00166)	< 0.001	0.10399 (0.05467)	1
Triglycerides																				
Total triglycerides	mmol/L	0.01738011	-0.1065 (0.00452)	< 0.001	-0.11728 (0.00566)	< 0.001	-0.08142 (0.00451)	< 0.001	-0.04874 (0.00448)	< 0.001	-0.04273 (0.00652)	< 0.001	-0.03966 (0.0048)	< 0.001	-0.10547 (0.00517)	< 0.001	-0.06248 (0.00168)	< 0.001	0.12329 (0.05293)	1
